# Site‐Specific Features Guiding Effective Reversible Photocontrol of Tryptophan Synthase With the Unnatural Amino Acid AzoF

**DOI:** 10.1002/cbic.70546

**Published:** 2026-09-23

**Authors:** Caroline Hiefinger, Sabine Laberer, Lukas Drexler, Cristina Duran, Michael Bartl, Sílvia Osuna, Andrea Hupfeld

**Affiliations:** ^1^ Institute of Biophysics and Physical Biochemistry and Regensburg Center for Biochemistry, University of Regensburg Regensburg Germany; ^2^ Institut de Química Computacional i Catàlisi and Departament de Química, Universitat de Girona Girona Spain; ^3^ Institució Catalana de Recerca i Estudis Avançats Barcelona Spain

**Keywords:** diazo compounds, enzyme catalysis, photocontrol, protein engineering, unnatural amino acids

## Abstract

This study delivers initial guidelines for the selection of effective incorporation positions for the azobenzene‐based unnatural amino acid AzoF to facilitate the successful engineering of light‐sensitive enzymes, dubbed photoxenases. Although photoxenases gain interest for the photocontrol of enzymatic activity across various fields their design has so far remained challenging. To ease particularly the selection of AzoF incorporation sites, we have systematically evaluated a total of 85 positions that differ in their biochemical and biophysical features in the bi‐enzyme model system tryptophan synthase. Medium‐throughput production of AzoF‐containing variants, activity‐based screening and evaluation of photocontrol, and subsequent validation of the screening results allowed us to identify three site‐specific features that promote photocontrol with a success rate of up to 83% when combined. These features include positions in domains with catalytically important conformational transitions, positions determined via molecular dynamics based shortest path map analysis, and positions that originally contain a hydrophilic side chain. Ultimately, our results underscore the complexity of predicting suitable incorporation sites but simultaneously provide a fundamental framework for the improved rational design of future photoxenases with AzoF.

## Graphical abstract

Reversible photocontrol of enzymes can be achieved with the azobenzene‐based photoswitchable unnatural amino acid AzoF. Screening of 85 AzoF incorporation positions in tryptophan synthase identified three features that appear to promote effective photocontrol with a success rate of up to 83%, which provides a simple guide towards the design of photocontrol in enzymes. SPM: computationally determined shortest path map.

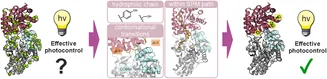

## 1 Introduction

In the past decades, genetic code expansion using photoswitchable unnatural amino acids (psUAAs) has emerged as a means for the reversible photocontrol of enzymatic activity. Upon irradiation, psUAAs typically isomerize between two configurations that facilitate different interactions with the surrounding protein environment when incorporated at a specific site [1, 2]. Despite various examples of photoxenoproteins [3] and specifically photoxenases [4], i.e., proteins and enzymes that were rendered light‐sensitive by a psUAA, the engineering approach has remained largely challenging. This particularly includes the design step of photoxenases, in which positions for the incorporation of the psUAA via amber suppression and an orthogonal aminoacyl‐tRNA synthetase (aaRS)/tRNA pair [5, 6, 7] are selected.

**FIGURE 1:**
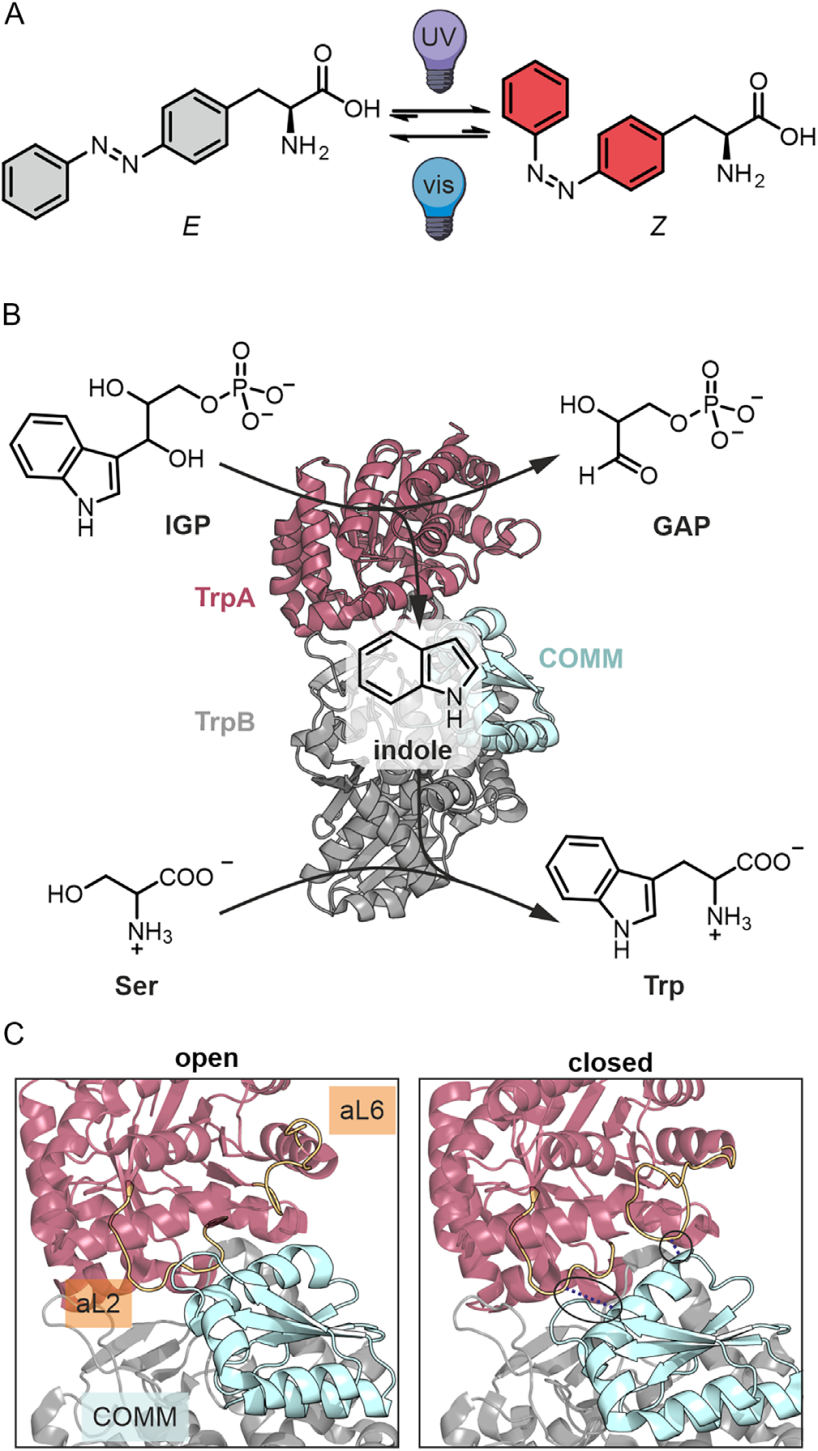
Tools for photoxenase engineering. (A) Isomerization of the azobenzene‐based psUAA AzoF upon irradiation with UV (365 nm) and visible light (420 nm). (B) The targeted model enzyme TS and its catalyzed partial reactions. The functional unit consists of a TrpA (red) and a TrpB (grey) subunit (PDB: 1WDW). (C) The conformational change of loop2 and loop6 in TrpA (aL2, aL6; orange) as well as the COMM domain (cyan) in TrpB from an inactive open (PDB: 1WDW) to an active closed conformation (PDB: 3CEP), which establishes two hydrogen bonds between the loops and the COMM domain (circled). These transitions are essential for the catalytic activity of TS. IGP: indole‐3‐glycerol phosphate; GAP: glyceraldehyde‐3‐phosphate; Ser: l‐serine; Trp: l‐tryptophan.

Most engineering studies have hitherto applied the psUAA phenylalanine‐4′‐azobenzene [1] (AzoF, Figure 1A). While the *E* isomer of AzoF predominates in the thermal equilibrium (TEQ), photocontrol is achieved by irradiation with 365 nm, which induces *E* → *Z* isomerization and results in the establishment of a *Z*‐enriched equilibrium called photostationary state (PSS^365^). Reversibility is accomplished by the re‐establishment of an *E*‐enriched PSS, facilitated by irradiation with 420 nm (PSS^420^). Hitherto, incorporation positions were selected based on certain assumptions, for example, that AzoF is most effective if it replaces large hydrophobic side chains [1, 8, 9, 10, 11]. Even advanced computational approaches have been used to predict incorporation sites [10, 11]. However, the successful identification of photoxenases from all incorporation sites selected in either rational or computational designs remained below 28%. As a result, numerous sites must still be experimentally tested to identify photoxenases, making the process time‐consuming and resource‐intensive. Predictive patterns obtained from a knowledge‐based approach might, hence, be highly beneficial to ease the selection of only few incorporation sites for effective photocontrol.

Our recent engineering achievements provided deeper insights into factors that might be connected to AzoF‐mediated photocontrol. First, enzyme targets with multiple catalytically relevant conformational populations indicative of conformational heterogeneity appear to be more susceptible to photocontrol with AzoF than those with one predominant population [4]. In fact, our two previously engineered enzyme targets, the allosteric imidazole glycerol phosphate synthase (ImGPS) and the nonallosteric asparaginase type II from *Escherichia coli* (EcAII), in the presence of the secondary substrate glutamine, demonstrate high conformational heterogeneity [12, 13]. Incorporation of AzoF adjacent or in domains with a catalytically relevant transition thereby obtained photoxenases with a success rate of 20% in both enzyme systems. Second, we have observed that the photocontrol efficiency may depend on environmental factors such as pH and salt concentration, which allowed us to increase the light regulation factor (LRF) from 1.5 to 2 in the initial screenings to 2–12 in both photoxenase systems [4, 14, 15]. Third, our results showed that photocontrol originates from a light‐induced conformational shift at the targeted active site and connected regions in ImGPS‐based photoxenases [16]. Notably, customization of this shift by semirational design yielded up to 10‐fold improved LRFs. Although the success of these engineering efforts suggests that location of AzoF close to catalytically relevant conformational transitions might benefit photocontrol, this hypothesis remains biased and uncorroborated. Moreover, our most recent studies revealed that psUAAs containing different, in part, heteroaromatic photoswitches can strengthen, decrease, or completely abolish photocontrol in the same position as AzoF [16, 17]. Hence, the direct interaction between a psUAA and the enzyme environment may be decisive for the occurrence of photocontrol (Figure S1), implying that various features of the incorporation site might benefit photocontrol and that these might be specific to the chosen psUAA.

Based on these insights, we here set out to determine general features of an effective incorporation site for photocontrol with AzoF. To this end, we chose a third model enzyme system, which exhibits conformational heterogeneity [18, 19, 20, 21, 22]. Tryptophan synthase (TS) is a bienzyme complex that catalyzes the last two steps in the biosynthesis of l‐tryptophan in bacteria, fungi, and plants [23, 24, 25]. Its two subunits, TrpA (a) and TrpB (b), are arranged in an abba architecture. In TrpA, indole‐3‐glycerol phosphate (IGP) is cleaved to glyceraldehyde‐3‐phosphate (GAP) and indole (Figure 1B). The latter is then transferred through an intermolecular channel to the active site of TrpB [25, 26, 27], where its condensation with l‐serine is mediated in a pyridoxal phosphate (PLP)‐dependent manner via formation of an aminoacrylate (AA) intermediate to finally yield l‐tryptophan [28, 29, 30]. Importantly, TrpA and TrpB mutually activate each other through allosteric communication, which is mediated by structural rearrangement from an inactive open to an active closed conformation (Figure 1C) [19, 31, 32, 33]. Associated conformational transitions primarily occur in loop2 and loop6 of TrpA (aL2 and aL6) as well as the communication (COMM) domain of TrpB, which establishes new interactions between both subunits [19, 31]. Notably, these motifs influence all three partial reactions: the TrpA (IGP → indole + GAP), the TrpB (indole + l‐serine → l‐tryptophan), and the TS (IGP → GAP + l‐tryptophan) reaction.

In this study, we investigated the site‐specificity of photocontrol with AzoF to identify general features of effective incorporation positions that achieve photocontrol. For this, we selected 85 equally distributed AzoF positions based on structural and computational insights in TS and screened the resulting variants for their light‐responsiveness of catalysis. We then searched for common features of the incorporation positions in the obtained photoxenases to determine a potential predictive pattern. Comparative analysis of the incorporation positions of TS, ImGPS, and EcAII confirmed the importance of the identified features.

## 2 Results and Discussion

### 2.1 Selection of AzoF Incorporation Positions

**FIGURE 2:**
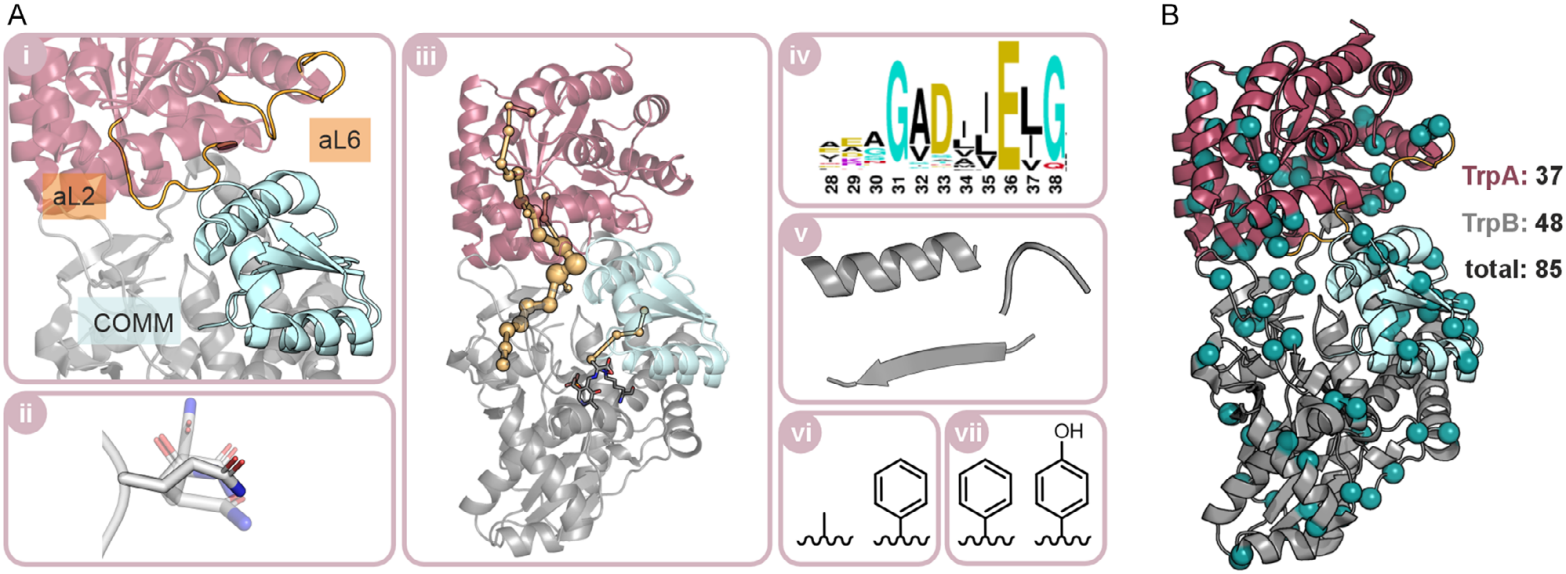
Site‐specific properties for the selection of 85 AzoF incorporation sites in TS. (A) Properties include: (i) proximity to the catalytically relevant conformational transitions of aL2, aL6, and the COMM domain (categories: in or outside each domain), (ii) side chain flexibility (categories: flexible or rigid determined by an RMSF analysis), (iii) proximity to an SPM residue (spheres; categories: Cα at/near or more than 5 Å distant), (iv) residue conservation (categories: conserved or not conserved according to an MSA), (v) secondary structure (categories: positioned in an α‐helix/β‐strand or a loop), (vi) side chain size (categories: small/medium‐sized or large); and (vii) side chain hydrophobicity (categories: hydrophobic or hydrophilic). Note: in (iii) only the SPM path connecting the TrpA and TrpB subunit is shown representatively; for SPM paths within TrpA and TrpB see Figure S4. (B) The positions (teal spheres) are evenly distributed throughout both subunits. PDB: 1WDW.

To identify features of effective incorporation positions for AzoF‐mediated photocontrol, we took a systematic approach. For this, we first selected seven properties for our analysis (Figure 2A), which were then grouped into two categories each to guide the subsequent analysis of correlations between site‐specific features and photocontrol. We then selected 85 positions in TS from *Pyrococcus furiosus* that are representative of these properties (Figure S2) and evenly distributed throughout the enzyme complex (Figure 2B).

AzoF mediates photocontrol by inducing a conformational shift [16]. Thus, comparable to our previous studies [4, 14, 15], the first grouping (i) included 23 positions within and 62 outside the aL2, aL6, and COMM domain that undergo conformational transitions. The dynamic character of such conformational transitions often requires high flexibility. To group the positions in TS according to this feature (ii), we determined the root mean square fluctuations (RMSFs) from MD simulations for each side chain. Considering a RMSF range throughout the full TS sequence of ≈0.2–3.0 Å and an average RMSF value of 0.6 Å (Figure S3), we annotated 22 positions with an RMSF value ≥0.7 Å as flexible (Table S1) and 63 with an RMSF value <0.7 Å as rigid. Furthermore, we included two features that enable the identification of positions critical for catalytically relevant transitions distant from the active site. The first is shortest path map (SPM) [34] analysis (iii), which is a correlation‐based tool that we have previously introduced to determine networks of residues linked to the catalytic site through conformational dynamics [31, 35, 36, 37]. In TS, we thus evaluated SPM networks within the isolated TrpA, monomeric TrpB, and dimeric TrpB subunits as well as within the heterodimeric TrpA‐TrpB functional unit. By this means, we then grouped 43 positions with the Cα atom either at or up to 5 Å near an SPM residue of either network and 42 positions with a distance of more than 5 Å (Figures 2A and S4; Table S2). The second is the evolutionary conservation of residue positions (iv). Using a multiple sequence alignment (MSA; Figures S5 and S6), we categorized 34 conserved and 51 not conserved positions. Another aspect to be considered is the secondary structure (v), which often defines the type of conformational transition. Hence, we further assorted 42 positions located in an α‐helix or β‐strand and 43 as part of a loop. Our previous data indicated that the direct interactions between the psUAA and the enzyme environment may be decisive for photocontrol (Figure S1). In this context, it has to be noted that most psUAAs are physicochemically different and bulkier in both configurations than natural amino acids. They also possess only a limited number of energetically preferred rotamers, which limits their potential for interactions. Especially, the commonly used AzoF is larger than any native amino acid and exclusively hydrophobic. For this reason, we included the size of the side chain (vi) grouped into small‐ to medium‐sized (34) or large side chains (51), and its hydrophobicity (vii) grouped into hydrophobic (40) or hydrophilic side chains (45).

In summary, the 85 selected positions for the incorporation of AzoF offer diverse and easily determinable properties and cover ~13% of the TS sequence with 37 positions in TrpA and 48 in TrpB.

### 2.2 Screening of 85 AzoF‐Containing TS Variants

Next, we set out to determine the photocontrol efficiencies for each of the 85 AzoF‐containing TS variants. To this end, we initially established a medium‐throughput screening procedure following three criteria, which we tested using a variant with generally high yields and wild‐type‐like activity, but no photocontrol upon irradiation, that was identified in preliminary studies. First, midi‐scale production of each TrpA or TrpB variant should obtain sufficient purity (>70%) and a high yield (Figure S7A,B). Incorporation of AzoF was thereby facilitated by amber suppression and a highly specific orthogonal aminoacyl‐tRNA synthetase/tRNA pair [5, 7, 38, 39]. We confirmed the successful incorporation of AzoF by UV/vis spectroscopy (Figure S7C) since our previous studies demonstrated that the detection of the AzoF‐specific UV/Vis signal is as reliable as mass spectrometry and because it is suited for medium‐throughput. Second, photocontrol efficiencies should be assessed in a real‐time photocontrol assay, in which the reaction progress of one sample is recorded first in the as‐isolated state (TEQ), then after 365 nm irradiation (PSS^365^; Table S3), and finally after 420 nm irradiation (PSS^420^; Table S3). Since potential deviations due to sample variations can be excluded in this setup, only single measurements for each variant were performed in this initial screening procedure. For this, irradiation durations to fully establish PSS^365^ (2 s) and PSS^420^ (7 s) were determined (Figure S8A), and the reaction temperature was optimized (Figure S8B) to 50 °C, which facilitates high activity of the thermostable TS from *P. furiosus* whilst preventing thermal relaxation of the metastable *Z* isomer of AzoF. Third, wild‐type (wt‐) TS should not show elevated deactivation upon irradiation, an effect that has been observed in PLP‐dependent enzymes [40] and specifically in TS from *Salmonella typhimurium* due to the degradation of PLP [41, 42]. Using the real‐time photocontrol setup, we followed the reaction progress of wt‐TS. Irradiation thereby induced a tolerable minor deactivation of up to 1.1‐fold for both the TrpA and the TrpB reaction (Figure S9).

With the optimized production conditions in midi‐scale, we obtained 77 soluble TrpA and TrpB variants with moderate to excellent expression yields of 0.8–65 mg per liter expression culture and moderate (>70%) to good (>85%) purities (Figures S2 and S10). Wt‐TrpA and wt‐TrpB were produced in liter scale with yields of 7.9 mg and 34 mg per liter expression culture and a purity of >95% (Figure S11A) to cover the demand for TS complex formation from all variants. To confirm the incorporation and switching of AzoF, UV/Vis spectra of all 77 variants were recorded in TEQ, PSS^365^, and PSS^420^ (Figures S12 and S13). The majority of all TrpA and TrpB variants exhibited absorbance signals at ~330 nm, which were decreased and reestablished in the PSS^365^ and PSS^420^, respectively, indicative of AzoF incorporation and switching. Two TrpA and seven TrpB variants displayed no detectable AzoF absorbance. However, the corresponding incorporation positions showed no consistent trends in physicochemical properties and were well distributed within the TS complex (Figure S14), which contradicts the possibility of a bundled site‐specific low incorporation efficiency. Since the lack of absorbance might also result from quenching by the enzyme environment or adjacent PLP cofactor rather than from the absence of AzoF, we preferred to include these nine variants in the subsequent investigation.

**FIGURE 3:**
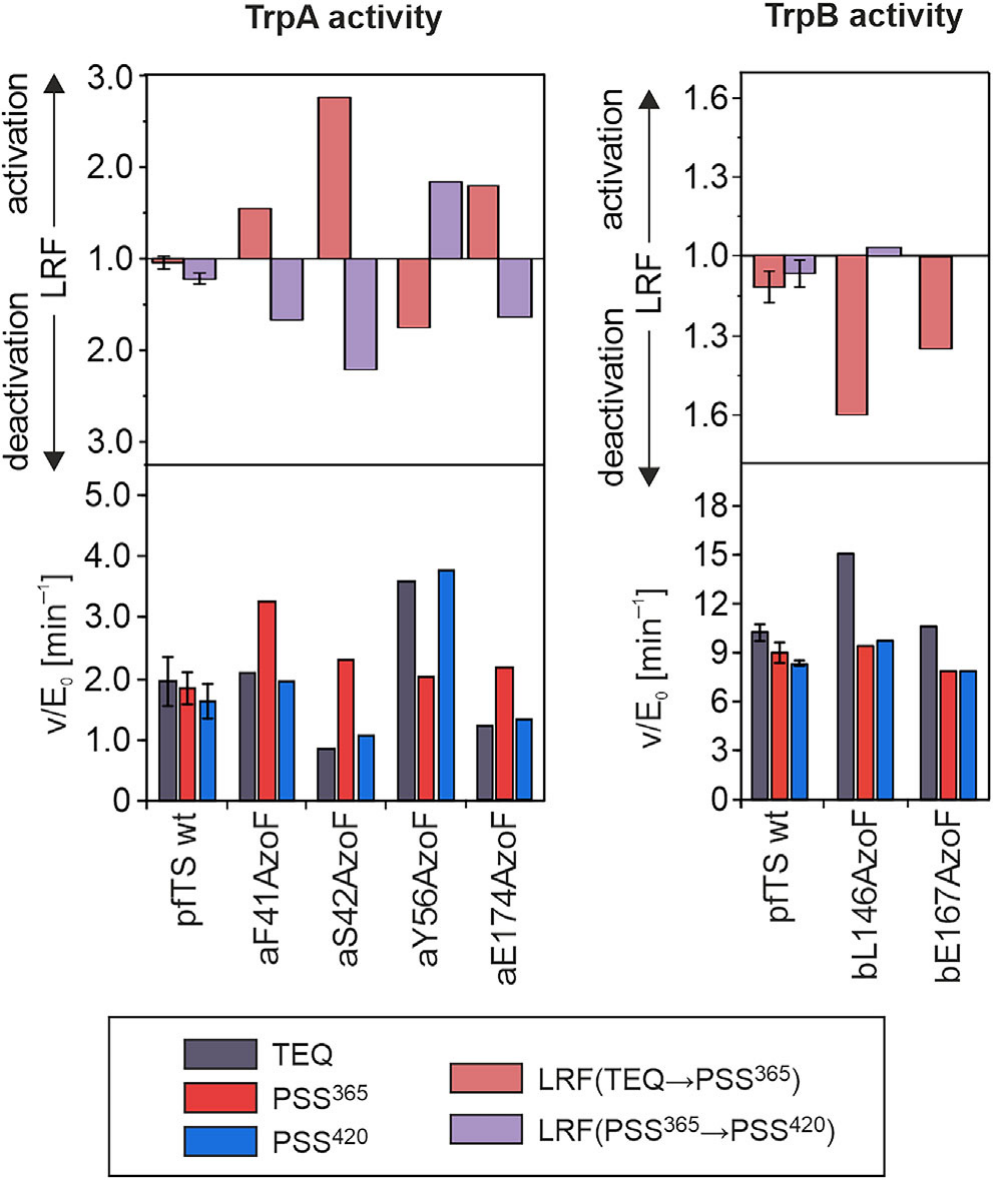
Screening results of the six highest ranked TrpA and TrpB variants. Bottom columns show the fitted catalytic rate normalized to the enzyme concentration (*v/E*_0_) in TEQ, PSS^365^ and PSS^420^ obtained from real‐time photocontrol measurements of TrpA activity, for all TrpA variants, and TrpB activity, for all TrpB variants. Top columns show LRF(TEQ → PSS^365^) and LRF(PSS^365^ → PSS^420^) calculated from the ratio of activities (v_1_/v_2_ with v_1_ > v_2_) between TEQ and PSS^365^ or PSS^365^ and PSS^420^, respectively. For reaction conditions see Figure S15.

In the next step, we screened the 77 variants for their photocontrol potential by retrieving the activity values for TEQ, PSS^365,^ and PSS^420^ from real‐time photocontrol measurements (Figures S15 and 3). To this end, we followed the TrpA reaction for all TrpA variants, and the TrpB reaction for all TrpB variants, for which our established continuous spectrophotometric detection assays were compatible with the reaction temperature of 50 °C. The photocontrol efficiency is primarily defined by the LRF, which designates the ratio of activities *v* before and after irradiation ($\frac{v1}{v2}$ with *v*_1_ > *v*_2_). To evaluate the LRFs of all variants, we used the respective ratios of wt‐TS (“apparent LRFs”) as a reference. Given that upon irradiation with 365 nm or 420 nm, wt‐TS was deactivated with apparent LRFs of up to 1.1 in both reactions (Figure S9), an LRF of ≥1.5 was regarded as threshold for effective photocontrol. Although 16 TrpA variants and four TrpB variants exceeded this threshold in at least the first of the two irradiation steps, some of them demonstrated very high LRFs >3 that appeared to be an artifact of overall extremely low activity close to the detection limit (Figure S15). Moreover, effective photocontrol is specified by the reversibility of photocontrol, meaning opposite regulatory effects upon 365 and 420 nm irradiation. Reversibility is thereby defined as the ratio of LRF(PSS^365^ → PSS^420^) and LRF(TEQ → PSS^365^) in percent. Notably, reversibility values might have been over‐ or underestimated considering the slight deactivation after irradiation with both wavelengths and for both the TrpA and TrpB reaction in wt‐TS. For this reason, we set out to validate the screening results by selecting potential photoxenases for large‐scale production. To this end, all variants were classified according to their LRF, activity, and reversibility of photocontrol receiving a score of 1–3 with 1 as the lowest and 3 as the highest value each (Figure S16). For each variant, the three scores were then multiplied with each other to yield an overall score (Tables S4 and S5). Of all variants tested, the four best‐ranked TrpA variants, namely, aF41AzoF, aS42AzoF, aY56AzoF, and aE174AzoF, were chosen for further investigations (Figure 3). For the TrpB variants, the scores were generally lower, and thus, the threshold for selecting potential photoxenases was adjusted accordingly, providing the two best‐ranked TrpB variants, bL146AzoF and bE167AzoF. All six variants demonstrated clear AzoF signals in the UV/Vis analysis (Figures S12 and S13). For the sake of clarity, we use the term a/bposAzoF from now on to refer to the TS complex containing AzoF at position pos in TrpA (a) or TrpB (b), e.g., aF41AzoF designates TrpA with AzoF in position 41 and in complex with wt‐TrpB.

In total, out of 85 selected variants, 77 could be obtained in a soluble state, for 68 AzoF incorporation was confirmed, and 20 exhibited an LRF of ≥1.5, of which the six highest ranked were selected for subsequent validation of the screening results.

### 2.3 Validation of the Screening Results

**FIGURE 4:**
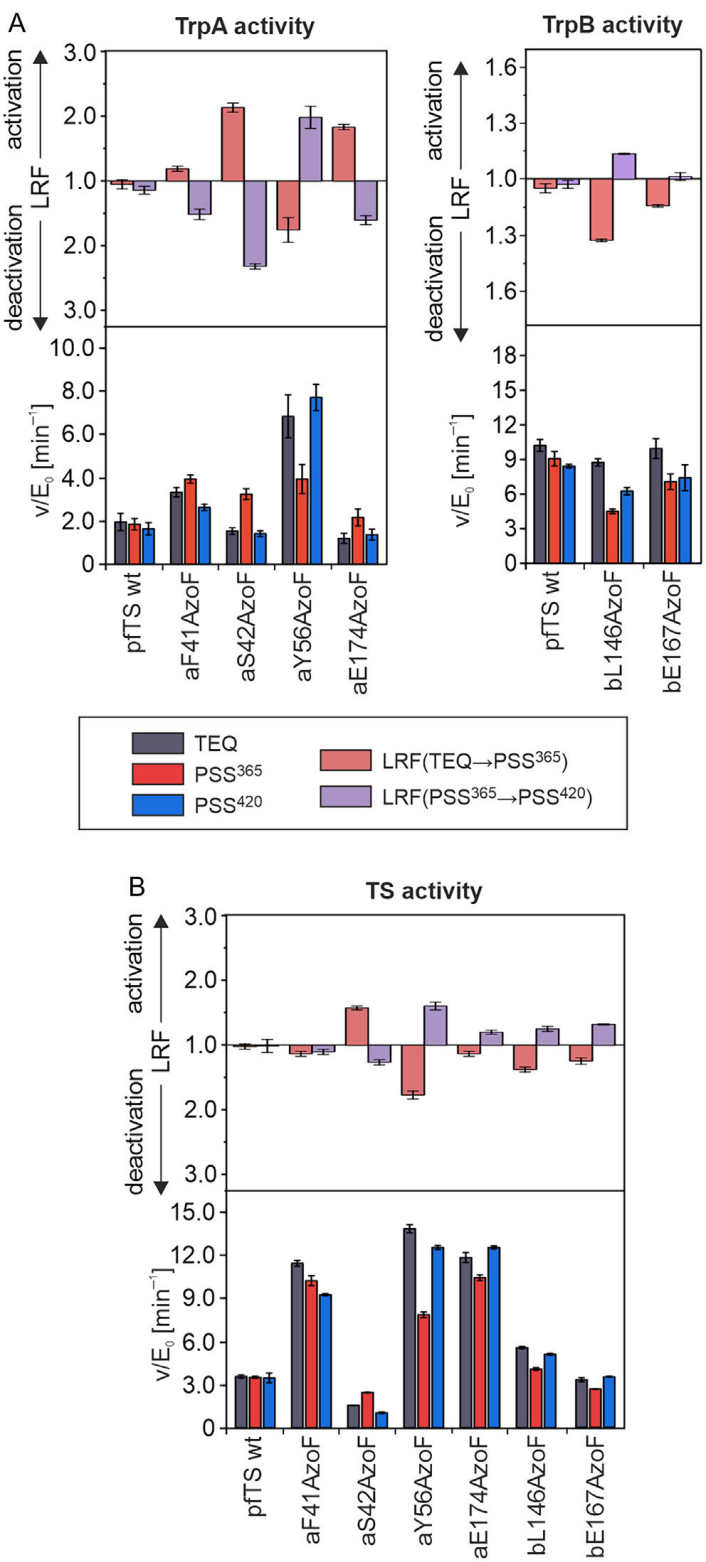
Partial (A) and coupled (B) reaction activities of the highest ranked TrpA and TrpB variants. Bottom columns show the catalytic rate normalized to the enzyme concentration (*v/E*_0_) in TEQ, PSS^365^ and PSS^420^ obtained from real‐time photocontrol measurements of: (i) TrpA activity (A), for all TrpA variants, (ii) TrpB activity (A), for all TrpB variants, and (iii) TS activity (B), for all variants. Values are provided either as mean ± standard deviation (S.D.; A) or mean ± standard error (S.E.; B) from technical triplicates. Top columns show LRF(TEQ → PSS^365^) and LRF(PSS^365^ → PSS^420^) calculated from the ratio of activities (v_1_/v_2_ with v_1_ > v_2_) between TEQ and PSS^365^ or PSS^365^ and PSS^420^, respectively. The values were obtained either from each single measurement and are given as mean ± S.D (A) or from the ratio of the respective mean activities and the error was calculated using quadratic error propagation (B). For reaction conditions see Figures S17 and S18.

We produced the six highest ranked TrpA and TrpB variants in large scale to validate i) their structural integrity, ii) the incorporation and switching behavior of AzoF, and iii) the effectiveness of photocontrol via technical triplicate measurements. The resulting proteins showed purities of >95% and an intact secondary structure comparable to wild type, as determined in circular dichroism measurements, confirming their structural integrity (Figure S11). Moreover, absorbance spectra of the respective variants were essentially identical to those obtained from midi‐scale‐produced proteins (Figures S17A and S18A). Irradiation with 365 nm for 2 s (Table S3) to establish the PSS^365^ thereby induced the formation of 64%–87% *Z* isomer, whereas subsequent irradiation with 420 nm for 7 s re‐established the *E*‐enriched PSS^420^ with 80%–94% *E* isomer. These isomer distributions are consistent with previously reported values of AzoF containing proteins [4, 14, 15]. Finally, we evaluated the photocontrol behavior of the selected TrpA and TrpB variants. Similar to the screening, we followed the TrpA reaction for each TrpA variant and the TrpB reaction for each TrpB variant in real‐time photocontrol setups using our continuous spectrometric assays for this purpose (Figures 4A, S17B and S18B). To capture the full impact of the light‐induced isomerization of AzoF on TS, we additionally analyzed the coupled TrpA and TrpB reaction (“TS reaction”; IGP → GAP + l‐tryptophan), which includes allosteric communication through conformational changes between TrpA and TrpB, for all TrpA and TrpB variants. For this, we followed tryptophan production via discontinuous chromatographic detection in a real‐time photocontrol setup (Figures 4B, S17C and S18C).

Overall, the real‐time photocontrol of TrpA and TrpB activity demonstrated similar behavior of each variant (Figure 4A) compared to the screening (Figure 3). Notably, wt‐TS was only minimally deactivated upon irradiation with an apparent LRF of ~1.1 in both the TrpA and TrpB reaction. In comparison, the variants were either activated (aF41AzoF, aS42AzoF, and aE174AzoF) or deactivated (aY56AzoF, bL146AzoF, and bE167AzoF) considerably with LRF (TEQ → PSS^365^) values of 1.2–2.1. Importantly, the photocontrol behavior of each variant, meaning whether it is activated or deactivated upon irradiation, was identical to the screening. Moreover, the actual activity values in TEQ (TrpA: 1.2–3.3 min^−1^; TrpB: 8.7–9.9 min^−1^) remained comparable to wt‐TS activity (TrpA: ~1.9 min^−1^; TrpB: ~10.2 min^−1^), although TrpA activities were slightly higher and TrpB activities marginally lower than in the screening. Finally, LRF(PSS^365^ → PSS^420^) values ranged between 1.0 and 2.3, resulting in reversibility values of <10% (bE167AzoF), 41% (bL146AzoF), 72% (aE174AzoF), and 100% (aF41AzoF, aS42AzoF, and aY56AzoF). Comparison of these properties identified aS42AzoF (activated) and aY56AzoF (deactivated) as best variants.

**FIGURE 5:**
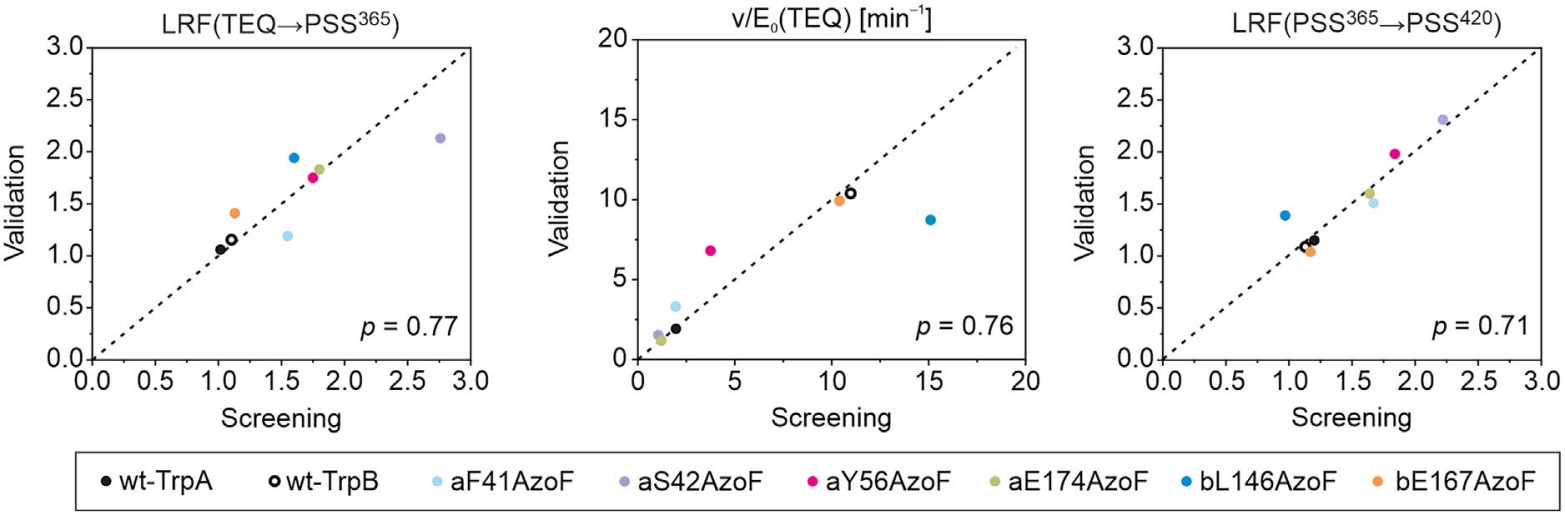
Comparison of the data sets obtained during screening (single measurements) and during validation (technical triplicates) with respect to LRF(TEQ → PSS^365^), activity in TEQ and LRF(PSS^365^ → PSS^420^) values. *p* values were determined using a paired *t*‐test. p ≥ 0.05 indicates no significant difference between the values obtained from screening and validation.

Next, we used the produced data to reassess the quality of our screening assay and, hence, confirm the reliability of activity and LRF values for the other 71 tested variants. Direct comparison of the LRF (TEQ → PSS^365^), activity, and LRF(PSS^365^ → PSS^420^) values from the screening (single measurements) and the validation (technical triplicates) showed high consistency between both data sets with *p* values of 0.71–0.77 (Figure 5). In terms of LRF(TEQ → PSS^365^), the greatest deviation was observed for aS42AzoF, yielding a value of 2.8 in the screening and a value of 2.1 in the validation. Regarding activity in TEQ, the largest deviation was observed for bL146AzoF (validation: 8.7 min^–1^; screening: 15.1 min^–1^), most likely due to inaccuracies in the determination of protein concentration during the screening procedure. Despite this deviation, the LRF is identical for this variant, confirming the assumption that the LRF is generally independent of the protein concentration. The LRF(PSS^365^ → PSS^420^) values of all variants determined during validation closely mirrored those observed in the initial screening.

We then set out to evaluate the photocontrol behavior of each variant during the TS reaction, for which we initially validated that chromatographic detection of the product tryptophan can be reliably employed for this purpose (Figure S19A–C). We then reconfirmed irradiation durations to reach both PSS in this setup and tested the chromatographic real‐time photocontrol on wt‐TS (Figure S19D–F), which demonstrated only a minimal decrease in activity upon both 365 and 420 nm irradiation with LRFs of ~1.0 (Figure 4B). Ultimately, the variants showed either decreased (aS42AzoF, aE174AzoF, bL146AzoF, and bE167AzoF) or similar (aF41AzoF and aY56AzoF) LRF(TEQ → PSS^365^) values of the TS reaction compared to the TrpA and TrpB reactions. Notably, the activity values in TEQ (1.6–5.6 min^−1^) were either comparable to wt‐TS (~3.6 min^−1^) or up to four‐fold higher (11.4–13.8 min^−1^) in aF41AzoF, aY56AzoF, and aE174AzoF. Furthermore, LRF (PSS^365^ → PSS^420^) ranged between 1.1 and 1.6, resulting in reversibility values of 0% (aF41AzoF), 46% (aS42AzoF), 68% (bL146AzoF), 79% (aY56AzoF), and 100% (aE174AzoF, bE167AzoF). Interestingly, the photocontrol behavior was reversed in the two variants aF41AzoF and aE174AzoF, indicating a deactivation upon irradiation instead of an activation as observed in the partial TrpA reaction. However, while aF41AzoF could not significantly activate l‐tryptophan production upon 420 nm irradiation, indicating rather a loss of photocontrol, aE174AzoF demonstrated full reversibility. Both effects, loss and inversion of photocontrol, are most likely associated with the allosteric network in TS. With this, aS42AzoF is the only remaining variant that is activated upon irradiation, and aY56AzoF is again the variant with the best photodeactivation.

Altogether, the strong correlation of photocontrol efficiency and activity in the TrpA and TrpB reactions suggested that the screening procedure provided highly reliable insights into, particularly the potential and effectiveness of photocontrol. Additionally, while the photocontrol behavior was in part reversed, photocontrol could be confirmed for all variants in the TS reaction except aF41AzoF. Hence, the screening served as a robust basis that now allows us to draw connections between biochemical and biophysical properties of the residue positions and effective photocontrol.

### 2.4 Identification of Site‐Specific Features Promoting Photocontrol

**FIGURE 6:**
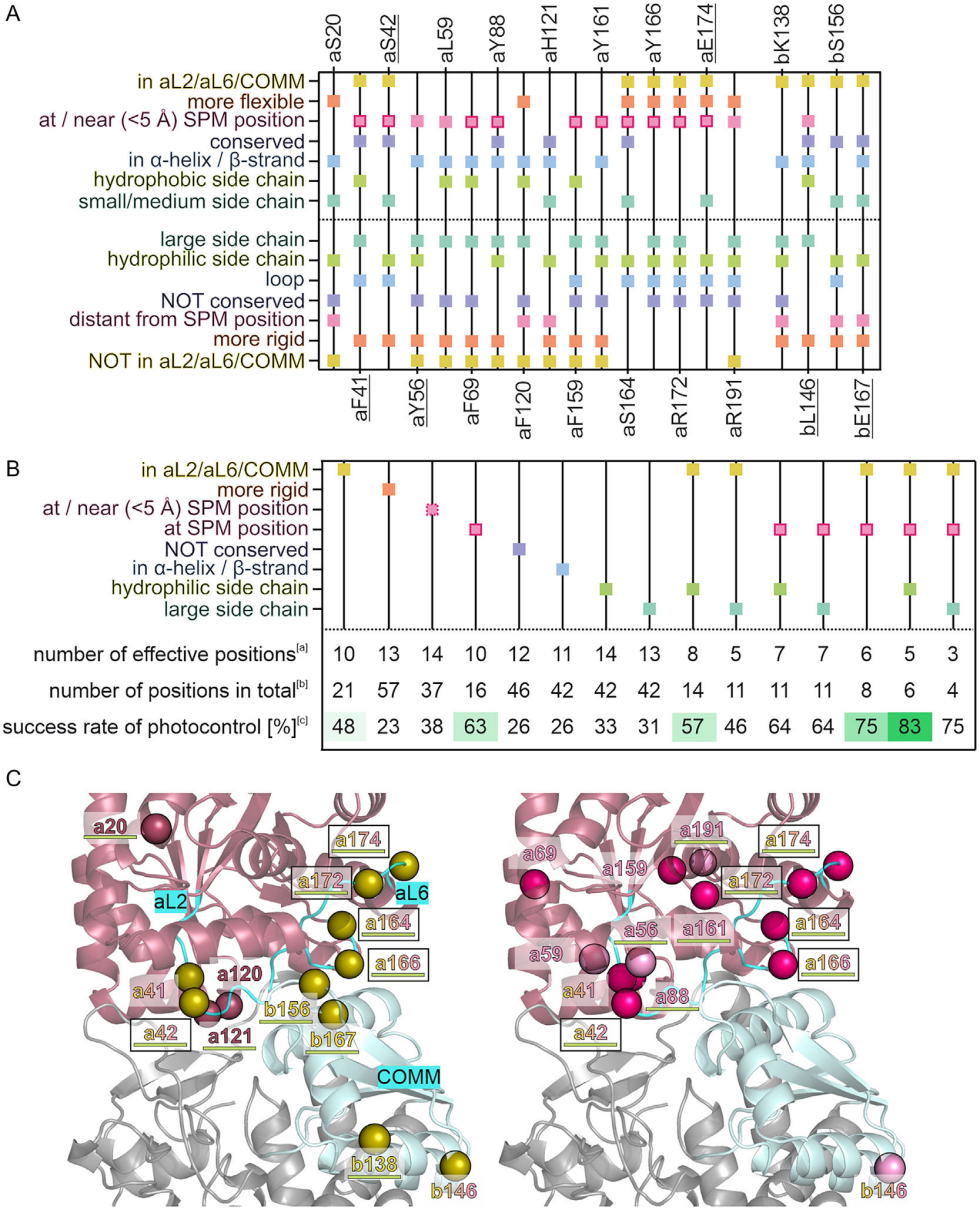
Identification of site‐specific features of AzoF incorporation sites that promote photocontrol. (A) Properties of AzoF incorporation sites in variants that generally showed an LRF ≥ 1.5 in the screening (Figure S15). The six validated TrpA and TrpB variants are underlined. (B) Determination of success rates for photocontrol promoted by different sets of site‐specific features. The most successful (combinations of) site‐specific features are highlighted in green with increasing intensity symbolizing higher chances of photocontrol. [a] Number of positions that possess the indicated site‐specific features, yielded soluble protein and facilitated an LRF ≥ 1.5 in the screening. [b] Total number of positions that possess the indicated site‐specific properties and yielded soluble protein. [c] Ratio of effective and total positions in percent. (C) AzoF incorporation sites in aL2/aL6/COMM (yellow spheres; left) and at (dark pink spheres) or near (pink spheres) SPM positions (pink spheres; right). Hydrophilic residue positions are underlined green and positions that are neither in aL2/aL6/COMM nor at/near SPM positions are indicated as red spheres (left). Sites that possess all three success criteria are boxed. PDB: 1WDW.

Finally, we set out to look for generalizable patterns for effective photocontrol. For this, we evaluated the biochemical and biophysical properties of all AzoF incorporation sites that facilitated an LRF ≥ 1.5 in the screening (Figure 6A), including the six validated variants (underlined). To ensure an unbiased comparison, we put the numbers of the effective positions in relation to the total number of AzoF incorporation sites that allowed for the production of soluble protein (marked white, Figure S2) for the more prevalent category of each site‐specific feature (Figure 6B). As a result, we obtained individual success rates for photocontrol. Interestingly, rigid positions, positions that are not conserved, and positions within an α‐helix or β‐strand were similarly or less effective (success rates of 23%–26%) than any previous rational or computational design criteria, which were successful in less than 28% [10]. Although this observation scrutinized the significance of said design approaches, it also provided a useful threshold of 28%, above which we choose properties for closer evaluation. Positions with hydrophilic or large side chains barely exceeded the threshold with success rates of 31%–33%. Impressively, positions in either aL2/aL6/COMM or at/near SPM positions (Figure 6C) demonstrated a clear tendency to facilitate photocontrol with success rates of 48% and 38%, respectively. Strikingly, a success rate of 63% was achieved by incorporating AzoFs directly into SPM positions.

Moreover, we determined whether the success rates might be further increased by combining the apparently most effective site‐specific features (Figure 6B). Hydrophilic or large side chains in aL2/aL6/COMM demonstrated either a slight increase in the success rate (57%) or no added value at all (46%). Comparably, combination of hydrophilic or large side chains with location at an SPM position showed no improvement of the success rate (45% and 42%). Next, we combined the two most effective site‐specific features, which identify SPM positions in dynamic domains, and obtained an already impressive success rate of 75%. This could be further improved to 83% if AzoF additionally replaces a hydrophilic side chain. Again, choosing large side chains had no additional beneficial effect. Importantly, the combination of features also decreased the number of positions that fulfil these criteria, thereby reducing the number of positions that need to be screened.

Thus, our analysis suggested three site‐specific features of AzoF incorporation positions that appear to be beneficial for effective photocontrol. First, localization in domains with a catalytically relevant conformational transition doubles the success rate of our previous criterion in ImGPS and EcAII photoxenase designs, in which sites close to such domains were also included. Second, positioning AzoF directly at an SPM residue appears to be highly effective. Similar to the first site‐specific feature, sites that are only close (<5 Å) to an SPM position are less expedient with only four AzoF incorporation sites that facilitated photocontrol with an LRF ≥ 1.5 (Figure S20). Both, location in a domain with conformational rearrangement as well as SPM positions, align well with the proposed mechanism of photocontrol via light‐induced conformational shifts [4, 14, 15]. In TS, aL2, aL6, COMM, as well as SPM positions have been associated with allosteric coupling essential for catalytic function and additionally have been used for designing standalone TrpA and TrpB variants in previous studies [26, 43]. Specifically, upon IGP binding to TrpA, aL2 and aL6 undergo order‐disorder transitions, stabilizing the subunit interface and the COMM domain [25, 32] and facilitating the transition from the open, inactive to the closed, catalytically active conformation (Figure 1C) [43]. Third, although AzoF is intuitively expected to be better accommodated in hydrophobic environments, the combination of the first two criteria with the replacement of hydrophilic side chains markedly increased the likelihood of achieving effective photocontrol. Remarkably, in three of the four ImGPS and EcAII photoxenases, AzoF also replaced hydrophilic side chains, further emphasizing this observation.

Ultimately, although additional studies will be needed to fully define general design principles across all photoxenases, we can already provide updated recommendations for photoxenase design with AzoF. In this context, it is crucial to note that our observations are based on enzyme targets with conformational heterogeneity. Furthermore, our recent studies indicated that the effectiveness of photocontrol differs if AzoF is irradiated with dichromatic light [44] or if it is replaced with another psUAA [16, 17]. Hence, the site‐specific features established here may provide a useful starting point for these systems, while their applicability should be validated experimentally. Importantly, despite the use of two allosteric model enzymes in our studies, the location of AzoF in dynamic domains or an SPM position is not exclusively connected to ligand‐induced allosteric activation. It is, however, generally applicable to enzymes, as both features describe the influence of distal sites on catalysis. Our data nicely demonstrate this as most effective positions were associated with an SPM path that was calculated only for the monomeric TrpA subunit or the dimeric TrpB subunit, which are independent of the allosteric communication between TrpA and TrpB (Figure S4). Moreover, both features provide independent predictive information since SPM paths pass through the enzyme without prevalence for catalytically relevant conformational domains (Figure S4). In conclusion, considering all three site‐specific features promises improved rational photocontrol designs with AzoF. In case of limited access to computational methods, future users may also resort to hydrophilic positions in dynamic domains as an easily accessible, but still auspicious, alternative. Moreover, the SPM tool might be valuable for the identification of effective AzoF incorporation positions in enzymes without a catalytically important conformational transition, but with conformational heterogeneity. Finally, as shown in previous studies, SPM proximity or localization within conformationally active regions corresponds to a successful approach for computational enzyme design workflows, including the design of photoxenases, as proposed in this study [31, 35]. Nevertheless, validation in other model enzymes and corroboration of the exact success rates are imperative, particularly to improve the input and, thus, the prediction quality of these possible future computational workflows.

While our study focuses solely on whether photocontrol was established, the selected TS‐based photoxenases, particularly aS42AzoF as the best variant and aF41AzoF and aE174AzoF that changed their photocontrol behavior in the TS reaction, are an excellent basis for more extensive engineering studies. The here reported limited LRFs of 1.5–2 might thereby increase with optimized reaction conditions [4, 15, 41] as well as through semirational design of the photoxenases [16] similar to the ImGPS and EcAII systems.

## 3 Conclusion

Site‐specific incorporation of psUAAs has become a highly promising method to design photocontrolled enzymes. However, predicting effective positions to establish significant photocontrol still remains a substantial challenge.

In this study, we selected 85 positions that are distributed throughout the well‐characterized model enzyme TS and that exhibit distinct biochemical and biophysical properties. Systematic screening of these positions allowed for a comprehensive analysis regarding AzoF incorporation and its photocontrol potential. Comparison of the features of effective positions revealed that photocontrol is frequently associated with localization either within structural regions involved in conformational rearrangements, such as aL2, aL6, and the COMM domain, achieving a success rate of 48%, or at SPM positions with a success rate of 63%. Albeit no generalizable pattern applies to all effective positions, combination of both criteria increased the success rate to 75% and even to 83% if only hydrophilic side chains are replaced by AzoF. These predictive patterns underscore the complexity of predicting suitable incorporation sites and provide markedly improved chances of identifying effective positions, while simultaneously reducing the number of candidates to be tested. Ultimately, combination of the three site‐specific features will guide the future design of photocontrol in various enzymatic systems.

## Funding

This work was supported by the Deutsche Forschungsgemeinschaft (KN 1462/1‐1), Fonds der Chemischen Industrie, Generalitat de Catalunya (SGR 2021 00487), Ministerio de Ciencia e Innovación (PID2021‐129034NB‐I00, PDC2022‐133950‐I00), HORIZON EUROPE European Research Council (ERC‐2022‐POC‐101112805, ERC‐2022‐CoG‐101088032, ERC‐2023‐POC‐101158166), and Spanish MINECO (PRE2019‐089147).

## Conflicts of Interest

The authors declare no conflicts of interest.

## Supporting information

The authors have cited additional references within the Supporting Information [45, 46, 47, 48, 49, 50, 51, 52, 53].

## Data Availability

The data that support the findings of this study are available from the corresponding author upon reasonable request.
