# Supplementary material for: Site‐Specific Features Guiding Effective Reversible Photocontrol of Tryptophan Synthase With the Unnatural Amino Acid AzoF
Source: Chembiochem. 2026 Sep 23;27(18):e70546. doi: 10.1002/cbic.70546 (PMC13599677; doi:10.1002/cbic.70546)
Supplement: Supplementary file 1 [file CBIC-27-e70546-s001.pdf]

Supporting Information

Site-Specific Features Guiding Effective Reversible Photocontrol of Tryptophan Synthase with the Unnatural Amino Acid AzoF

Caroline Hiefinger,[a] Sabine Laberer,[a] Lukas Drexler,[a] Cristina Duran,[b] Michael Bartl,[a] Sílvia Osuna,*[b,c] and Andrea Hupfeld*[a]

[a] Dr. C. Hiefinger, S. Laberer, L. Drexler, M. Bartl, PD Dr. A. Hupfeld (née Kneuttinger)
Institute of Biophysics and Physical Biochemistry and Regensburg Center for Biochemistry
University of Regensburg
Universitätsstraße 31, D-93053 Regensburg (Germany)
E-mail: andrea.hupfeld@ur.de

[b] Dr. C. Duran, Prof. S. Osuna
Institut de Química Computacional i Catàlisi and Departament de Química
Universitat de Girona
c/ Maria Aurèlia Capmany 69, 17003 Girona (Spain)
E-mail: silviaosu@gmail.com

[c] Prof. S. Osuna
ICREA, Pg. Lluís Companys 23, 08010 Barcelona (Spain)

Table of Contents

1. Supporting material 4

Figure S1. 4
Figure S2. 5
Figure S3. 6
Table S1. 7
Figure S4. 8
Table S2. 9
Figure S5. 10
Figure S6. 11
Figure S7. 12
Table S3. 12
Figure S8. 13
Figure S9. 14
Figure S10. 15
Figure S11. 17
Figure S12. 19
Figure S13. 21
Figure S14. 21
Table S4. 22
Table S5. 23
Figure S15. 16
Figure S16. 25
Figure S17. 26
Figure S18. 27
Figure S19. 28
Figure S20. 29
Figure S21. 30
Figure S22. 30
Figure S23. 31

2. Experimental section 32

2.1 Synthesis of phenylalanine-4'-azobenzene (AzoF) 32
Scheme S1. Synthesis of phenylalanine-4'-azobenzene (AzoF) 32

2.2 Synthesis of indole-3-glycerol phosphate (IGP) 33

2.3 Expression and Purification of glyceraldehyde 3-phosphate dehydrogenase (GAPDH) from Thermotoga maritima 33

2.4 Irradiation devices 34

2.5 Multiple sequence alignments 34

2.6 Molecular dynamics simulations-based analyses 34

2.7 Determination of biochemical and biophysical properties of residue positions 38

2.8 Subcloning and site-directed mutagenesis 38

2.9 Midi-scale production of TrpA and TrpB variants 39

2.10 Large scale production of TrpA and TrpB variants 39

2.11 Circular dichroism spectroscopy 40

2.12 UV/Vis spectroscopy 40

2.13 Estimation of isomer ratios from UV/Vis spectra 41

2.14 Real-time photocontrol of the TrpA reaction 42

Scheme S2. 42
2.15 Real-time photocontrol of the TrpB reaction *43*
2.16 Photocontrol of the TS reaction *43*
2.17 Classification of TrpA and TrpB variants *44*
References **46**

1. Supporting material

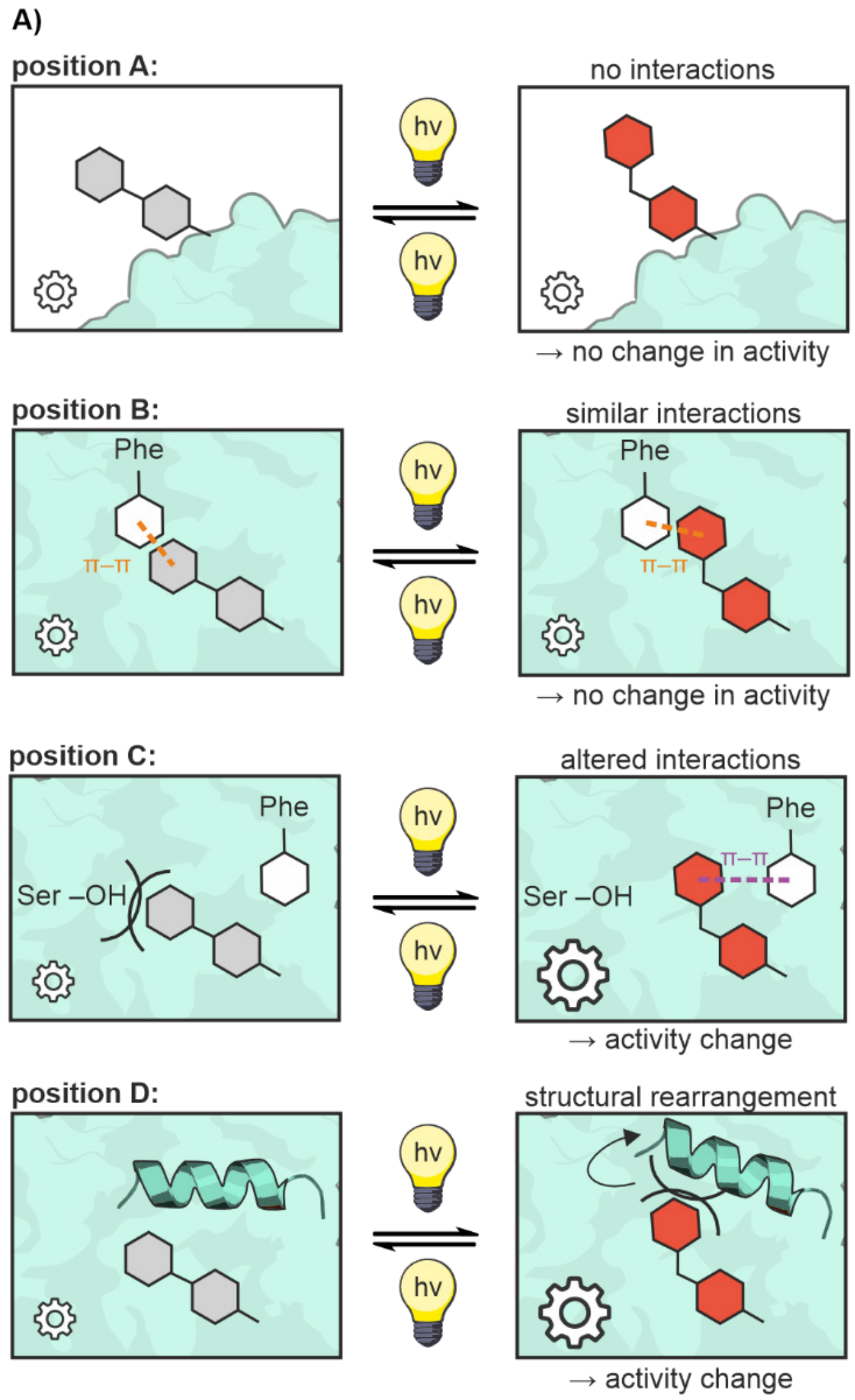

Figure S1. Hypothetical basis for the site-specificity of photocontrol with psUAAs. Direct interaction between the psUAA and the enzyme environment may be decisive for photocontrol. Only positions, in which the light-induced configurational switch of the psUAA results in altered interactions or induces structural rearrangements, may mediate a change in activity. Small gear: low activity; large gear: high activity.

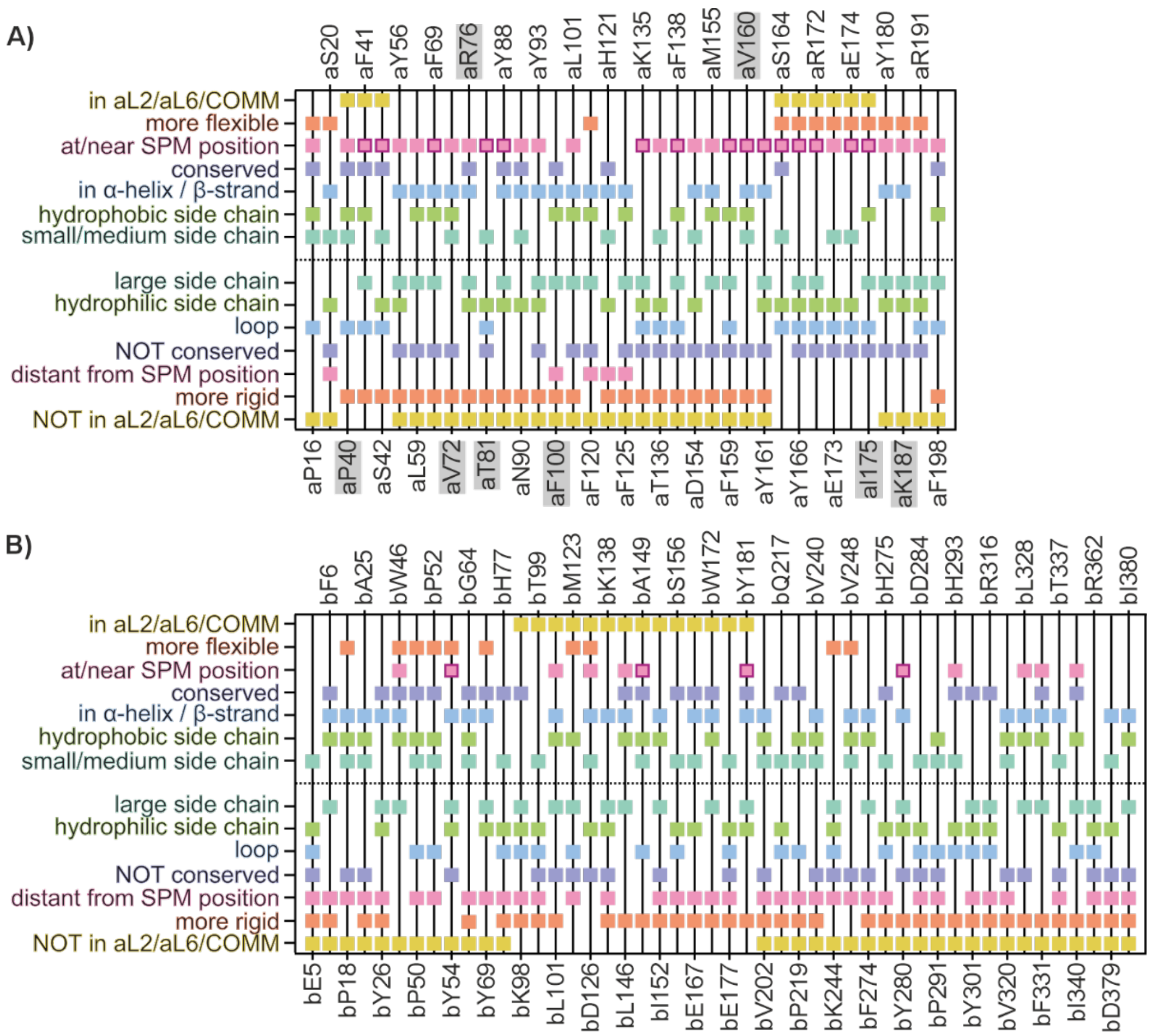

Figure S2. Selection of 85 positions in TrpA (A) and TrpB (B) for the incorporation of AzoF. The selected positions cover a wide range of site-specific features: 23 (62) are (not) located in the allosterically active aL2, aL6 and COMM domain; 22 are flexible (RMSF ≥ 0.7 Å; **Table S1**) and 63 rigid (RMSF < 0.7 Å); 43 are at (indicated by a blue border) or near (<5 Å) a position that is included in the shortest path map (SPM; **Table S2**) and 42 are more distant (>5 Å) to an SPM position; 34 (51) are (not) conserved; 42 are located in an α-helix or a β-strand and 43 in a loop; 34 have a small or medium and 51 a large side chain; 40 contain a hydrophobic and 45 a hydrophilic side chain. AzoF incorporation yielded 77 soluble protein variants (marked white; **Figure S10**), and 8 insoluble protein variants (marked grey).

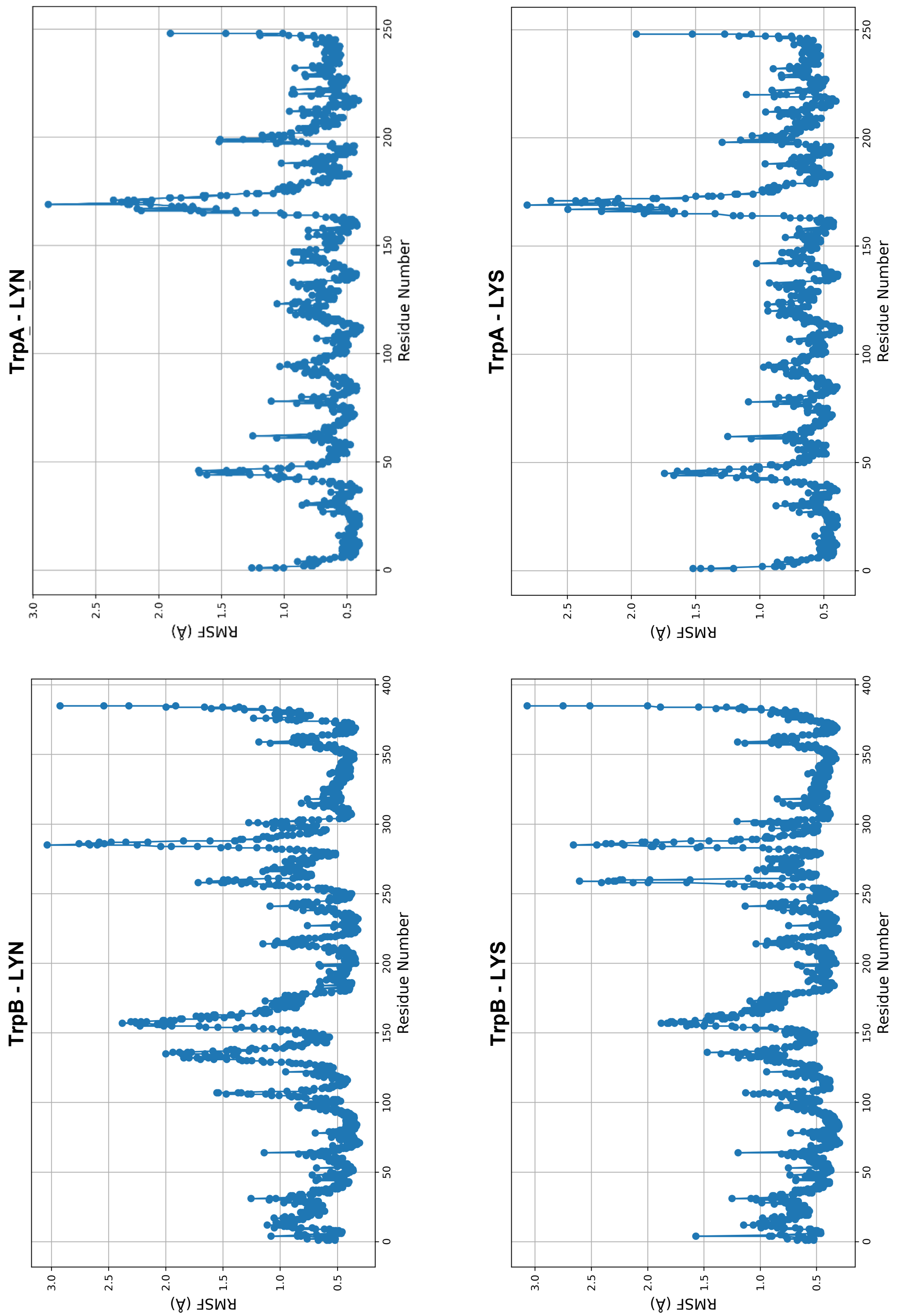

Figure S3. Root mean square fluctuation (RMSF, in Å) values throughout the full TrpA and TrpB sequences determined from molecular dynamics (MD) simulations with a sodium cation and the aminoacrylate intermediate bound in TrpB. The PLP-binding residue bK84 was used in two states: neutral (LYN) and protonated (LYS). The final RMSF values shown in **Table S1** are a combination of the values for LYN and LYS.

Table S1. Root mean square fluctuation (RMSF, in Å) values that were used to group the selected positions in TrpA and TrpB into more flexible (RMSF ≥ 0.7; orange) or more rigid (RMSF < 0.7; white). The average RMSF of all positions is 0.6 Å, therefore only positions with RMSF ≥ 0.7 Å were considered as flexible.

TrpA position	RMSF	TrpB postition	RMSF
**16**	0.879	**5**	0.514
**20**	0.792	**6**	0.532
**41**	0.450	**18**	0.859
**42**	0.489	**25**	0.486
**56**	0.502	**26**	0.496
**59**	0.448	**46**	0.905
**69**	0.475	**50**	0.933
**72**	0.490	**52**	0.943
**76**	0.492	**54**	0.971
**81**	0.427	**64**	0.694
**88**	0.457	**69**	0.713
**90**	0.437	**77**	0.590
**93**	0.398	**98**	0.606
**100**	0.485	**99**	0.479
**101**	0.434	**101**	0.564
**120**	0.866	**123**	0.979
**121**	0.537	**126**	0.802
**125**	0.531	**138**	0.555
**135**	0.468	**146**	0.685
**136**	0.536	**149**	0.577
**138**	0.485	**152**	0.512
**154**	0.452	**156**	0.478
**155**	0.474	**167**	0.408
**159**	0.582	**172**	0.470
**160**	0.658	**177**	0.455
**161**	0.636	**181**	0.452
**164**	0.783	**202**	0.410
**166**	0.885	**217**	0.449
**172**	1.126	**219**	0.412
**173**	1.033	**240**	0.576
**174**	1.129	**244**	0.717
**175**	1.285	**248**	0.774
**180**	1.707	**274**	0.377
**187**	0.984	**275**	0.385
**191**	0.826	**280**	0.421
**198**	0.697	**284**	0.426
		**291**	0.387
		**293**	0.376
		**301**	0.417
		**316**	0.509
		**320**	0.462
		**328**	0.425
		**331**	0.340

	**337**	0.350
	**340**	0.392
	**362**	0.389
	**379**	0.554
	**380**	0.656

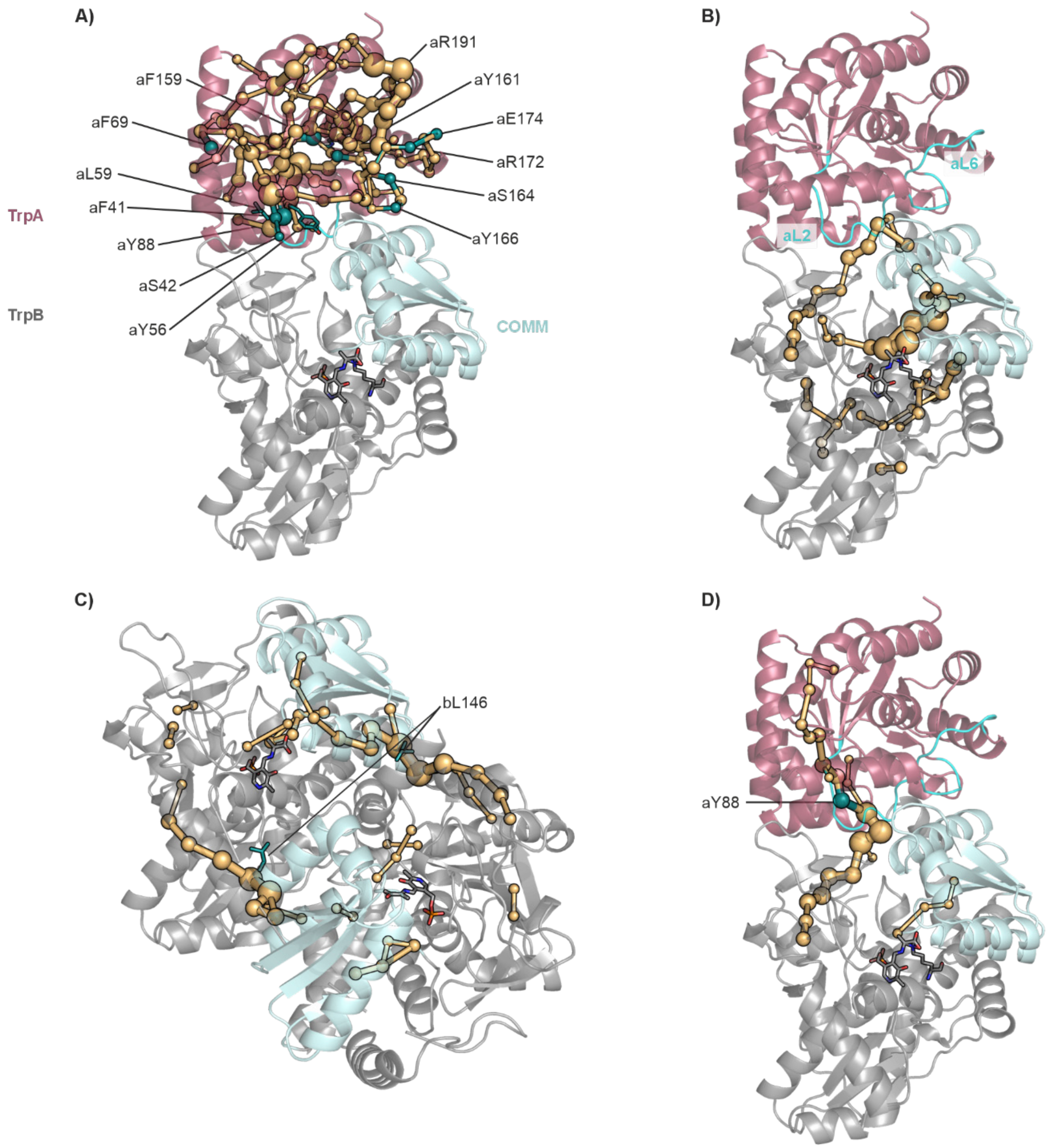

Figure S4. Shortest path map (SPM) analysis within the TrpA subunit (A), the monomeric TrpB subunit (B), the dimeric TrpB unit (C) and the heterodimeric TrpA-TrpB functional unit (D; cf. **Figure 2A**). Yellow spheres and lines indicate SPM conformationally relevant positions (see SPM description in methods for more details). SPM positions, in which incorporation of AzoF established photocontrol with an LRF ≥ 1.5 are highlighted in teal (spheres). AzoF incorporation sites near SPM positions that established photocontrol with an LRF ≥ 1.5 are highlighted as teal sticks.

Table S2. Mean distances to the nearest SPM residue computed along the MD trajectories that were used to group the selected positions in TrpA and TrpB into i) at (pink box) and near (pink shade) a SPM residue (<5 Å) or ii) distant thereof (white). Distances were computed taking as reference the starting AF3 model.

TrpA position	nearest SPM residue	distance [Å]	TrpB postition	nearest SPM residue	distance [Å]
**16**	17	3.873	**5**	279	8.68
**20**	17	5.341	**6**	279	5.514
**41**	41	0	**18**	181	10.163
**42**	42	0	**25**	181	14.067
**56**	55	3.843	**26**	182	13.491
**59**	58	3.867	**46**	45	3.859
**69**	69	0	**50**	45	9.935
**72**	71	3.861	**52**	430	5.291
**76**	79	4.979	**54**	54	0
**81**	81	0	**64**	333	14.71
**88**	88	0	**69**	54	5.536
**90**	89	3.885	**77**	45	10.082
**93**	92	3.847	**98**	181	10.442
**100**	103	5.257	**99**	125	9.122
**101**	104	4.906	**101**	125	4.907
**120**	118	5.591	**123**	125	6.461
**121**	118	5.62	**126**	125	3.853
**125**	128	5.299	**138**	149	14.04
**135**	135	0	**146**	147	3.864
**136**	135	3.846	**149**	149	0
**138**	138	0	**152**	149	9.729
**154**	153	3.87	**156**	181	19.126
**155**	156	3.878	**167**	181	11.31
**159**	159	0	**172**	181	6.485
**160**	160	0	**177**	181	11.447
**161**	161	0	**181**	181	0
**164**	164	0	**202**	186	14.553
**166**	166	0	**217**	54	15.549
**172**	172	0	**219**	54	13.802
**173**	172	3.837	**240**	186	15.789
**174**	174	0	**244**	54	22.331
**175**	175	0	**248**	264	13.934
**180**	181	3.862	**274**	279	7.816
**187**	186	3.878	**275**	279	9.485
**191**	192	3.838	**280**	280	0
**198**	197	3.851	**284**	281	9.658
			**291**	262	8.72
			**293**	262	4.668
			**301**	265	8.443
			**316**	280	16.756
			**320**	264	8.446

	**328**	329	3.869
	**331**	332	3.85
	**337**	335	5.739
	**340**	335	4.847
	**362**	54	16.948
	**379**	332	14.085
	**380**	332	13.174

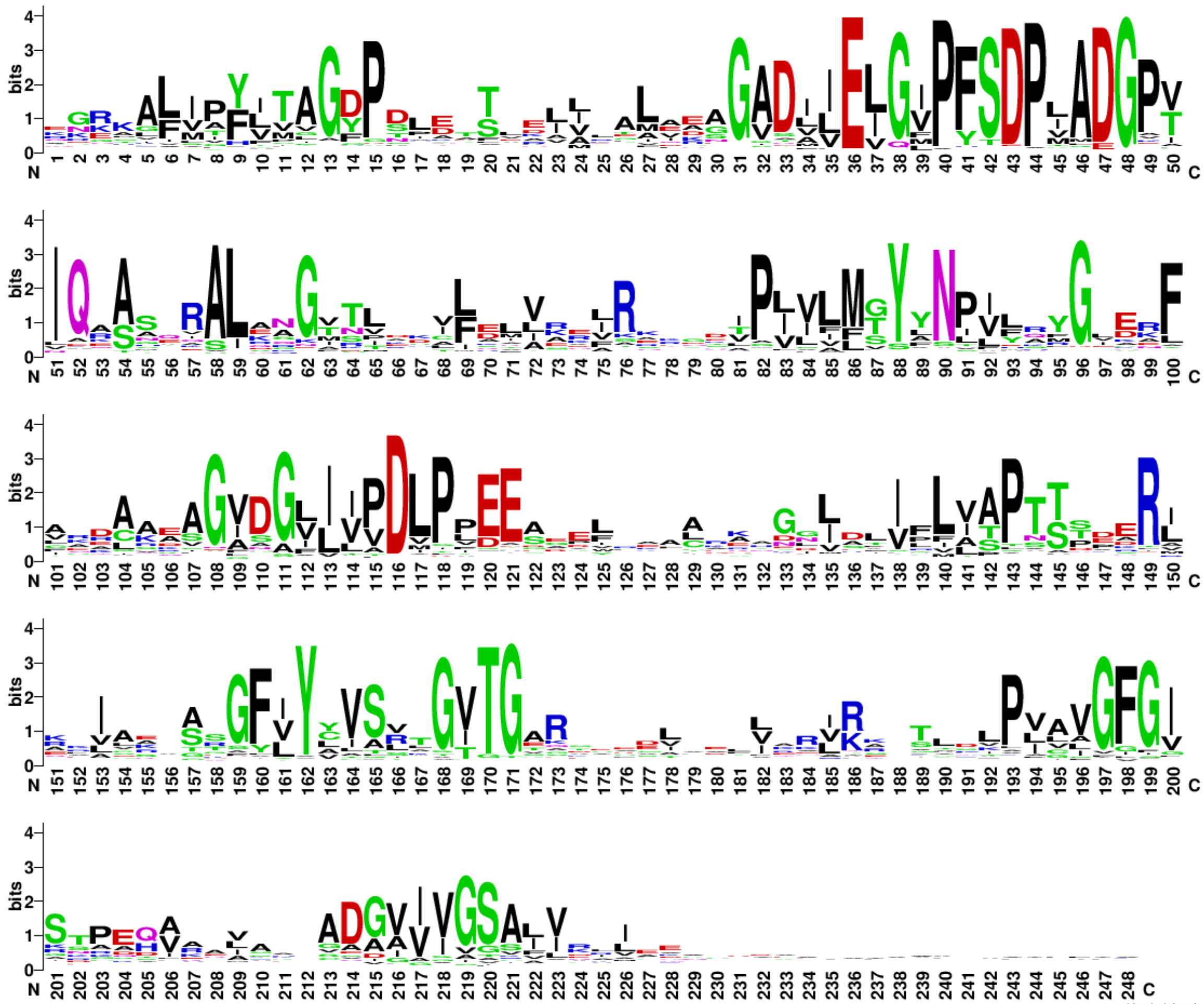

Figure S5. Sequence logo of TrpA generated from a multiple sequence alignment (MSA) of 1976 sequences of the respective InterPro family (IPR002028). The height of each letter indicates the relative frequency of the corresponding amino acid at that position. The sequence of TrpA from *Pyrococcus furiosus* served as reference for numbering. Residues with a score of at least 3 bits were considered as “conserved”.

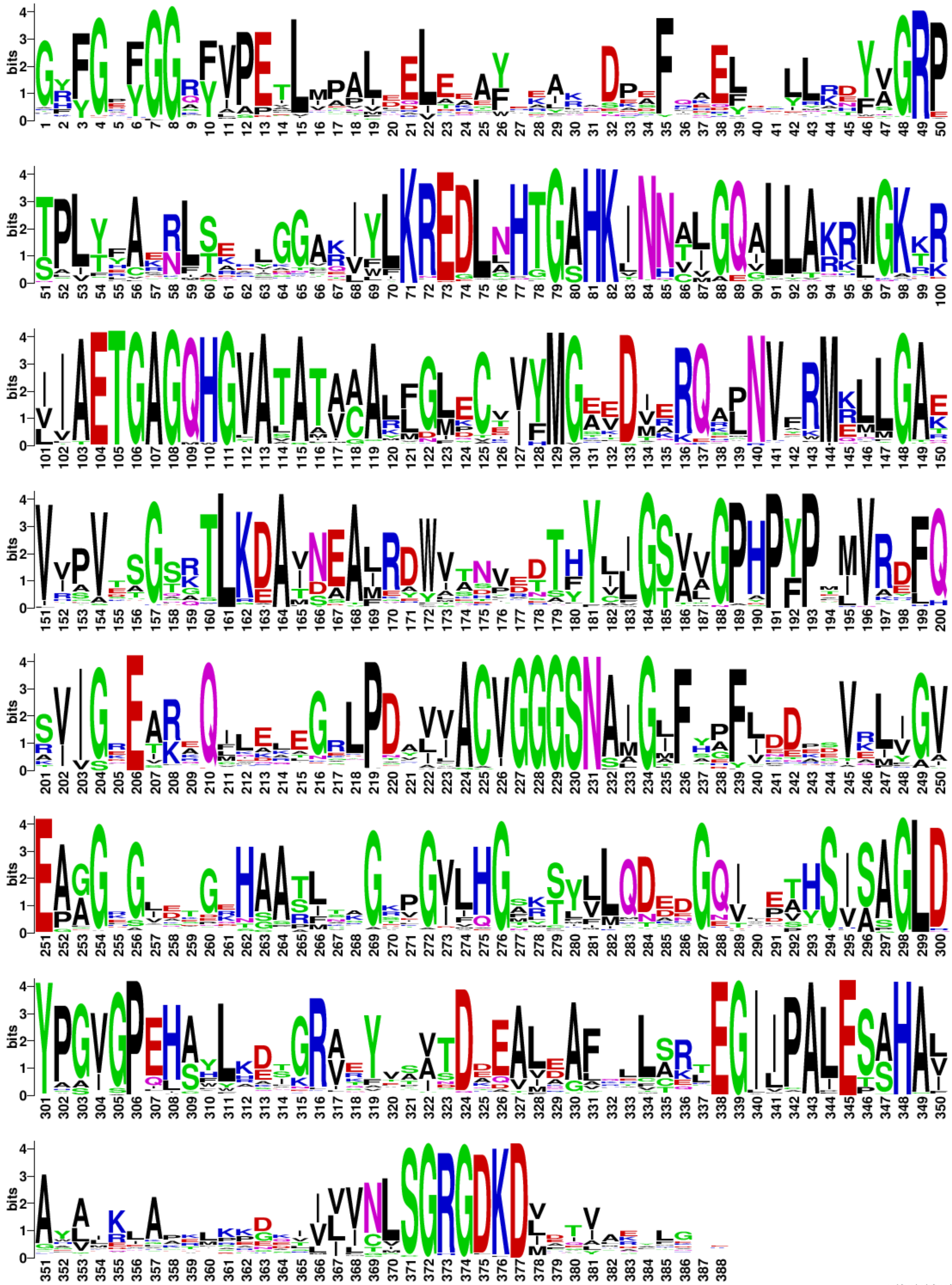

Figure S6. Sequence logo of TrpB generated from an MSA of 1731 sequences of the respective InterPro family (IPR006654). The height of each letter indicates the relative frequency of the corresponding amino acid at that position. The sequence of TrpB from *P. furiosus* served as reference for numbering. Residues with a score of at least 3 bits were considered as “conserved”.

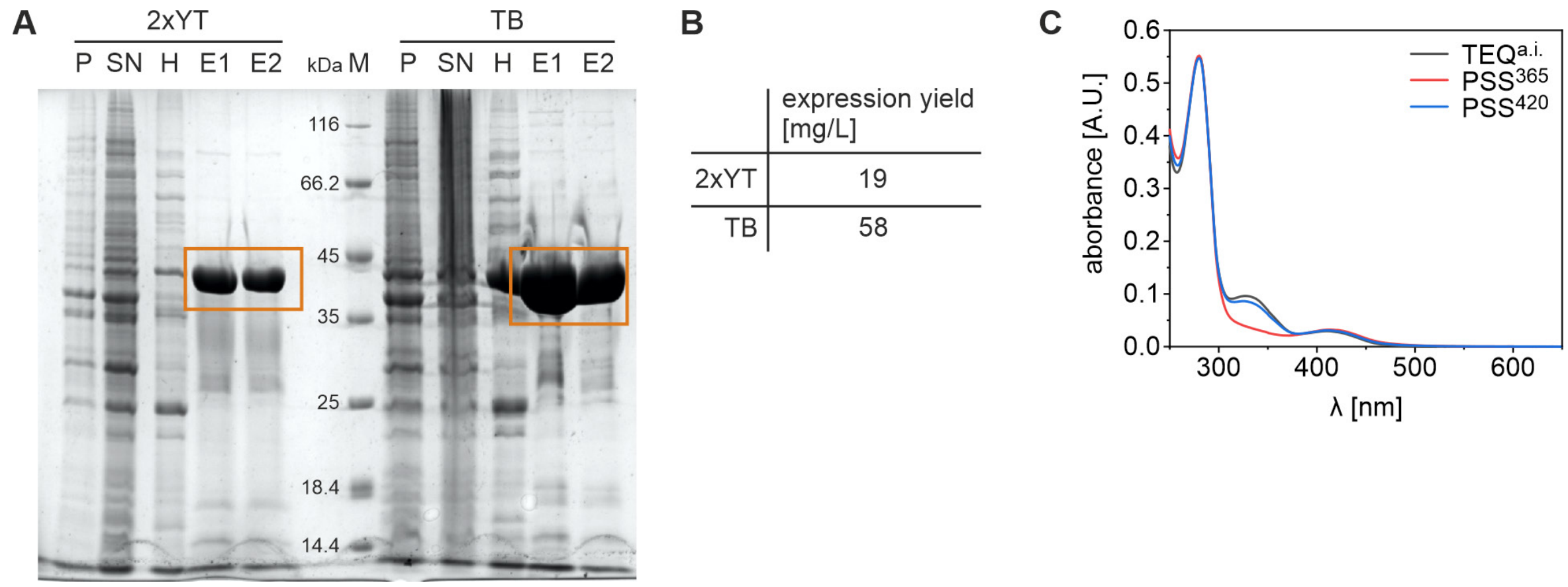

Figure S7. Establishment of midi-scale production of AzoF-containing enzyme variants using bY10AzoF, a variant with generally high yields and wild type-like activity, but no photocontrol upon irradiation, identified in preliminary, unpublished studies. A) 13.5% SDS-PAGE showing the influence of 2xYT or TB medium on the production of bY10AzoF via heterologous gene expression. Bacterial lysates were purified via heat treatment and Ni^{2+}-affinity chromatography in midi-scale. P: pellet; SN: supernatant; H: sample after heat-step; E1, E2: eluates after affinity chromatography. For visualization, the SDS-PAGE was stained with Coomassie Brilliant Blue G250 for 15 min and excess dye was removed by several washing steps in water. B) Quantification of protein yield in mg/L expression medium confirmed exceptionally high amounts of protein when the expression strain was cultivated in Terrific Broth (TB) medium. C) Validation of AzoF incorporation via UV/Vis spectroscopy evident from its intrinsic absorbance at ~330 nm (π–π* transition) and ~430 nm (n–π* transition).[1–3] *Reaction conditions*: 20 µM bY10AzoF [50 mM potassium phosphate (KP) pH 7.5, 100 mM KCl] in TEQ and after irradiation to obtain PSS^{365} (2 s 365 nm; **Table S3**) and PSS^{420} (7 s 420 nm; **Table S3**).

Table S3. LEDs used in this work.

High power LEDs	λ_{max} [nm]	I_{max} [mA][a]	P_{max} [mW][b]
LED Engin, LZ4-04UV00	365	700	250 mW/cm^2
Avonec 1W	420	350	60 mW/cm^2

[b] Intensity was determined with a UVA+ sensor (Opsytec, 0–2000 mW cm^{-2}, 300–450 nm) directly in front of the LED.

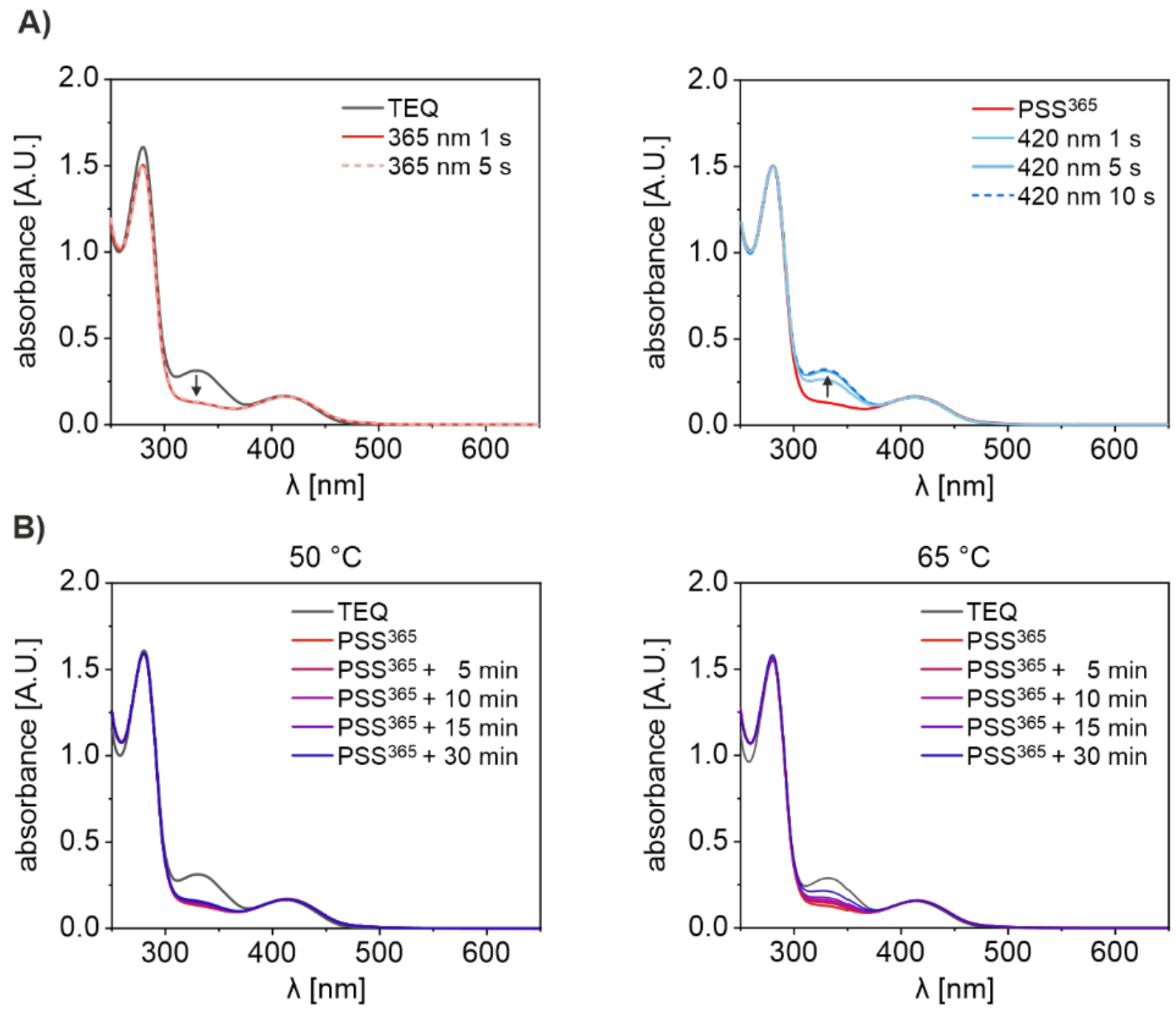

Figure S8. Requirements for a real-time photocontrol assay. A) Determination of irradiation durations via UV/Vis spectroscopy required to completely establish PSS^{365} (left) and PSS^{420} (right) in a quartz cuvette of 1 cm depth. PSS^{365} was reached after 1 s of 365 nm irradiation and PSS^{420} after 5 s of 420 nm irradiation. To ensure full PSS formation, 2 s 365 nm and 7 s 420 nm irradiation durations were chosen for subsequent real-time photocontrol experiments. *Reaction conditions*: 40 µM bY10AzoF (50 mM KP pH 7.5, 100 mM KCl) in TEQ and after 365 nm and 420 nm irradiation (**Table S3**). B) Assessment of thermal relaxation of the PSS^{365} in AzoF via UV/Vis spectroscopy necessary to choose the reaction temperature. While incubation at 65 °C led to significant thermal relaxation, 50 °C was deemed suitable for subsequent measurements as long as the duration of the experiment does not exceed 30 min. *Reaction conditions*: 40 µM bY10AzoF (50 mM KP pH 7.5, 100 mM KCl) at 50°C or 65°C.

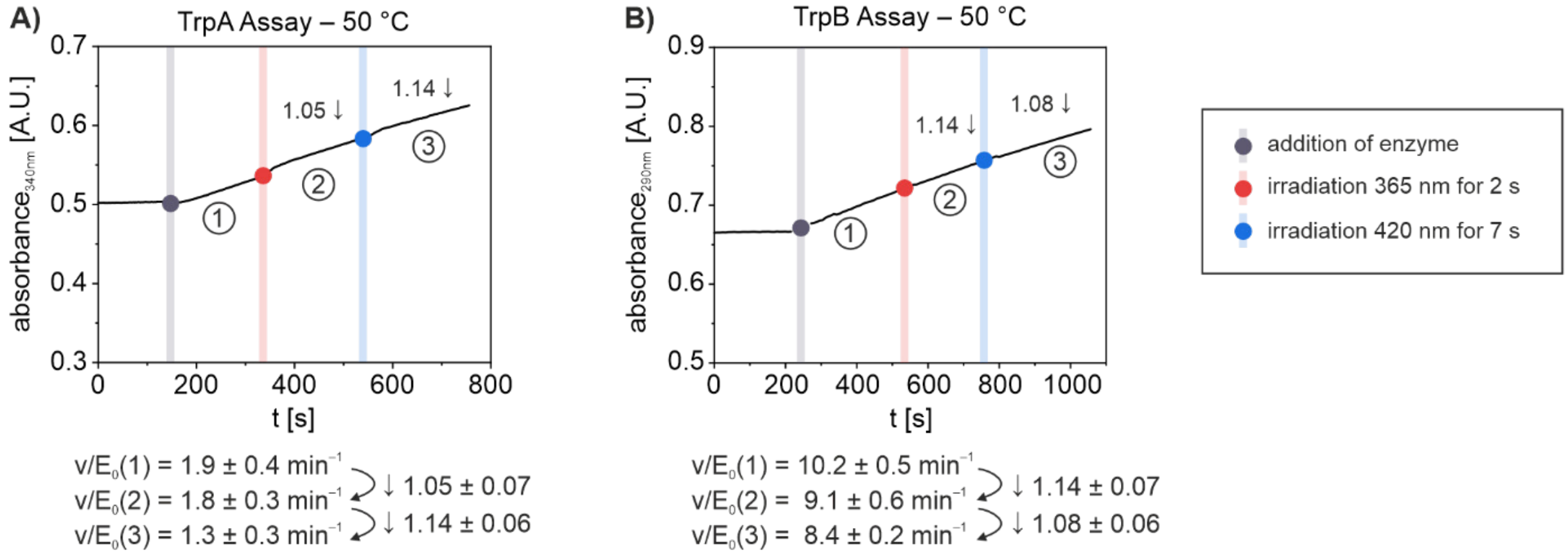

Figure S9. Evaluation of wt-TS photosensitivity in real-time photocontrol experiments in a quartz cuvette of 1 cm depth following the TrpA (A) and TrpB (B) activity. All assay components except the enzyme were pre-incubated, and a baseline was recorded. Subsequently, the reaction was started by addition of wt-TS (grey dot) and the reaction progress was recorded for 3 min (phase 1). The reaction was irradiated with 365 nm light for 2 s (red dot; **Table S3**) and its progress was recorded for another 3 min (phase 2). Finally, the reaction was irradiated with 420 nm light for 7 s (blue dot) and its progress was recorded for additional 3 min (phase 3). Activities were determined as reaction velocity *v* per initial enzyme concentration E_0 for each reaction phase. The ratios of activities v/E_0 between phase 1 and phase 2 or phase 2 and phase 3 (v_1/v_2 with $v_1 > v_2$), respectively, are given. *TrpA reaction conditions*: 80 µM pyridoxal phosphate (PLP), 6 mM NAD^+, 20 mM Na_3AsO_4, 5 µM glyceraldehyde-3'-phosphate dehydrogenase (GAPDH), >250 µM indole glycerol phosphate (IGP; saturated) and 1 µM wt-TS in 50 mM KP pH 7.5, 100 mM KCl at 50 °C. *TrpB reaction conditions*: 40 mM L-serine, 250 µM indole, 40 µM PLP and 1 µM wt-TS in 50 mM KP pH 7.5, 100 mM KCl at 50 °C.

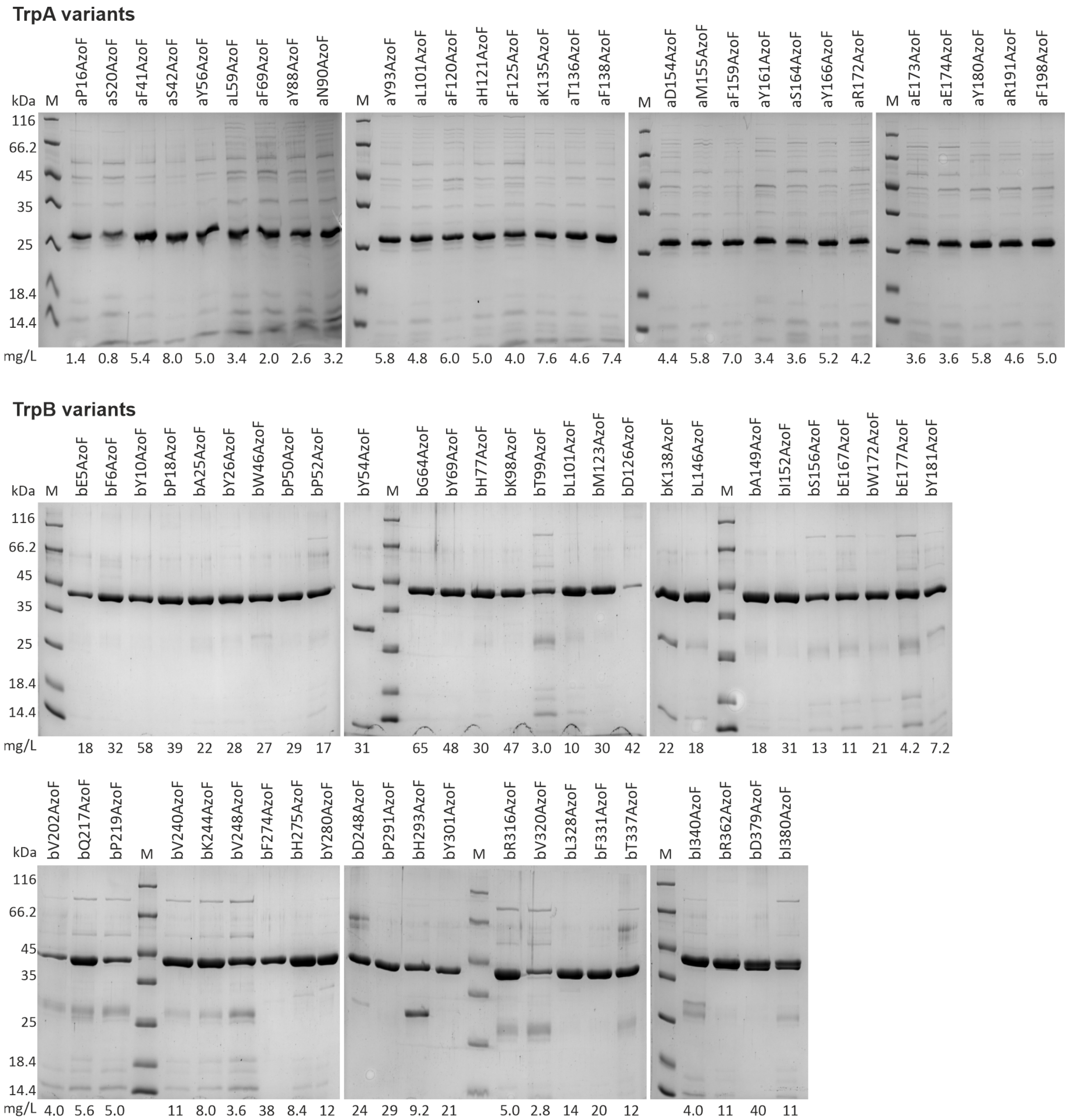

Figure S10. 13.5% SDS-PAGEs of the 77 soluble TrpA and TrpB variants produced in the screening. Most of the variants exhibited >85% purity, while some showed less purities of >70% (e.g. bK138AzoF). Yields are given in mg per liter expression culture below the pictures and were moderate for all TrpA variants, while TrpB variants could be obtained in moderate to excellent yields. For visualization, the SDS-PAGE was stained with Coomassie Brilliant Blue G250 for 15 min and excess dye was removed by several washing steps in water.

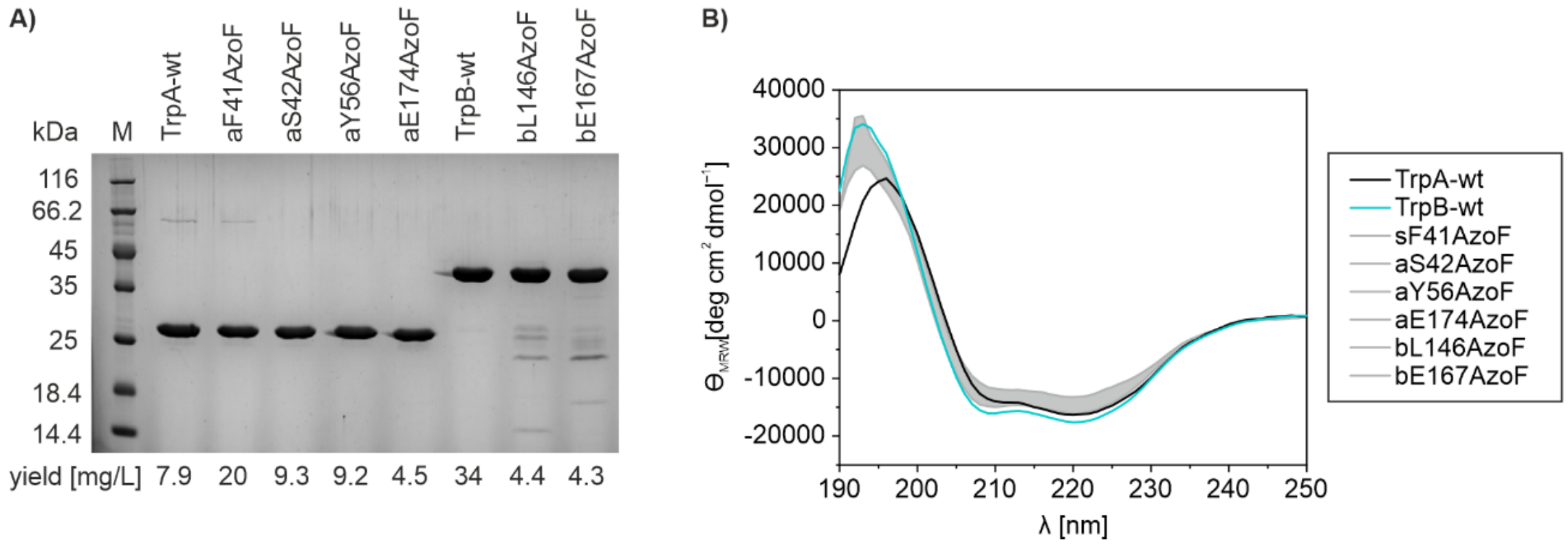

Figure S11. Confirmation of purity and structural integrity of TrpA and TrpB variants produced in large scale. (A) SDS-PAGE analysis of purified proteins. 7 µL low-molecular weight marker (M) and 3 µg of each protein were applied to the gel. All variants showed purities of >95%. Yields are given in mg per liter expression culture. (B) Circular dichroism spectra of 8-12 µM TrpA and TrpB variants in 50 mM KP pH 7.5, 100 mM KCl confirmed their structural integrity. For the sake of simplicity, essentially similar spectra were combined in the grey shaded area.

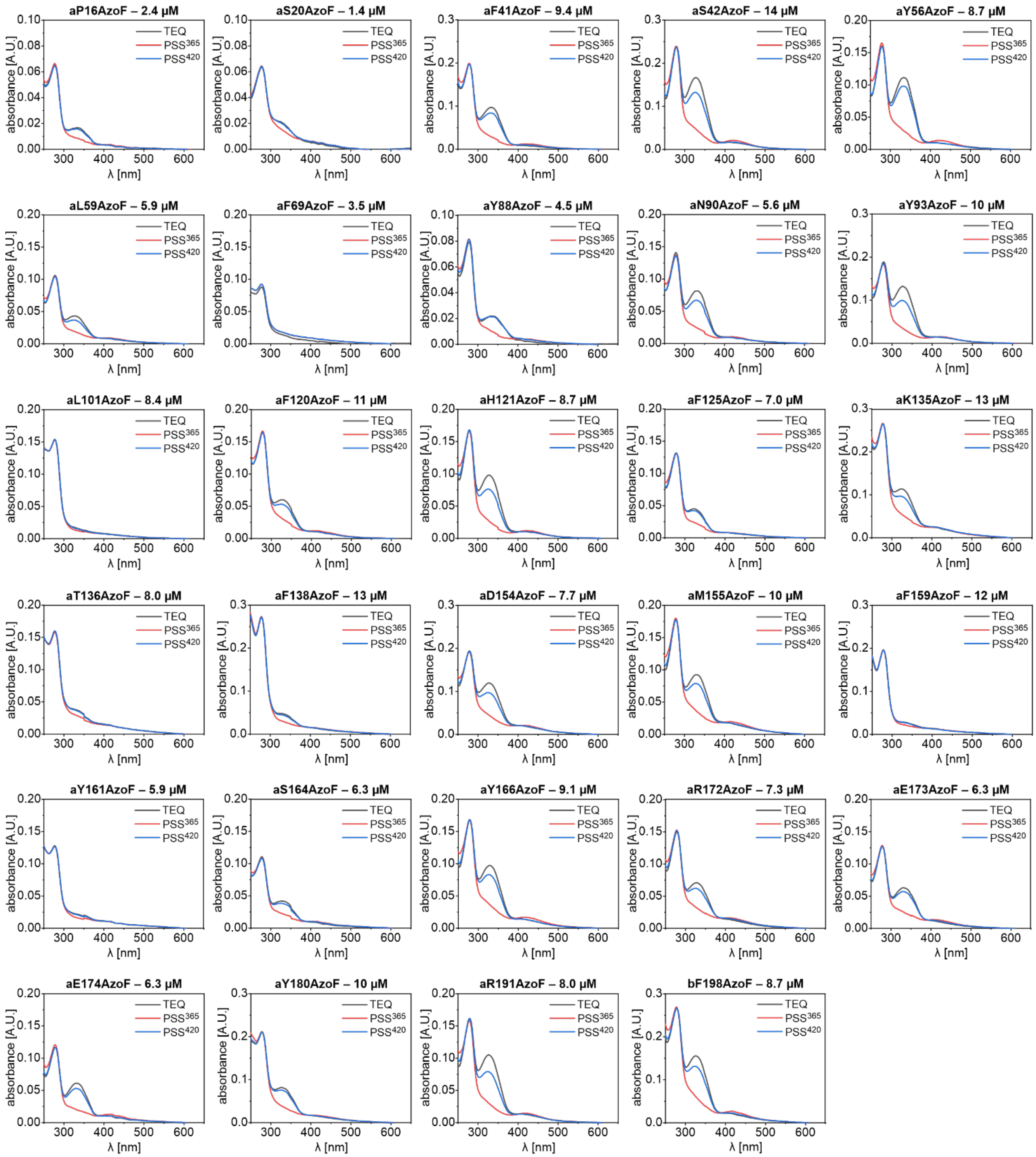

Figure S12. Absorbance spectra of purified AzoF-containing TrpA variants in TEQ, PSS^{365}, and PSS^{420}. The majority of variants showed more or less pronounced signals at ~330 nm (π–π* transition) and ~430 nm (n–π* transition). Intensity differences in AzoF absorbance result from different amounts of impurities as well as from quenching effects caused by the local enzyme environment. AzoF incorporation and switching could not be confirmed, possibly because of such effects, for aF69AzoF and aL101AzoF. *Reaction conditions*: protein in 50 mM KP pH 7.5, 100 mM KCl (concentrations given above each panel) in its TEQ and after 2 s 365 nm as well as 7 s 420 nm irradiation (**Table S3**).

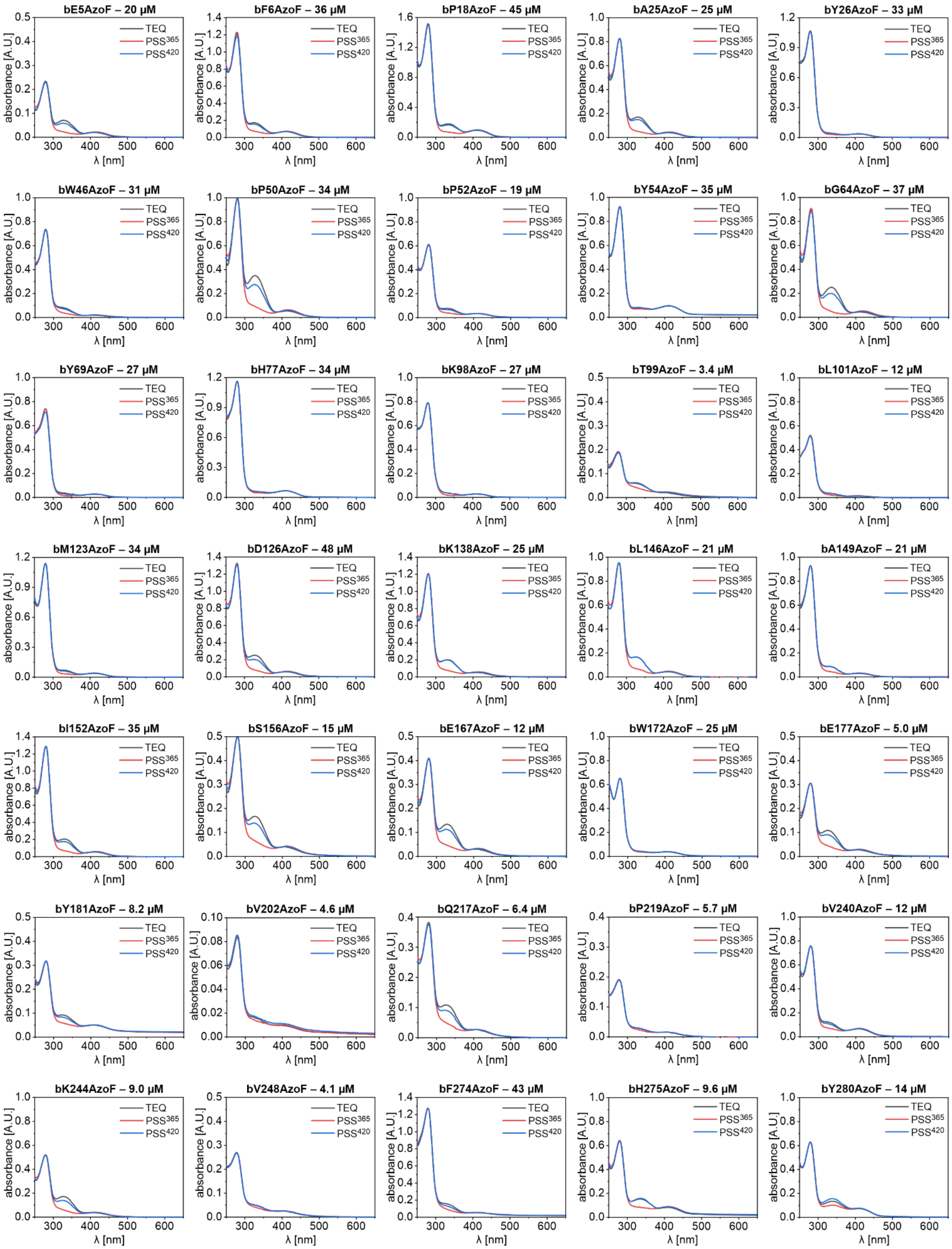
bE5AzoF – 20 µM
bF6AzoF – 36 µM
bP18AzoF – 45 µM
bA25AzoF – 25 µM
bY26AzoF – 33 µM
bW46AzoF – 31 µM
bP50AzoF – 34 µM
bP52AzoF – 19 µM
bY54AzoF – 35 µM
bG64AzoF – 37 µM
bY69AzoF – 27 µM
bH77AzoF – 34 µM
bK98AzoF – 27 µM
bT99AzoF – 3.4 µM
bL101AzoF – 12 µM
bM123AzoF – 34 µM
bD126AzoF – 48 µM
bK138AzoF – 25 µM
bL146AzoF – 21 µM
bA149AzoF – 21 µM
bI152AzoF – 35 µM
bS156AzoF – 15 µM
bE167AzoF – 12 µM
bW172AzoF – 25 µM
bE177AzoF – 5.0 µM
bY181AzoF – 8.2 µM
bV202AzoF – 4.6 µM
bQ217AzoF – 6.4 µM
bP219AzoF – 5.7 µM
bV240AzoF – 12 µM
bK244AzoF – 9.0 µM
bV248AzoF – 4.1 µM
bF274AzoF – 43 µM
bH275AzoF – 9.6 µM
bY280AzoF – 14 µM
TEQ
PSS365
PSS420
absorbance [A.U.]
λ [nm]

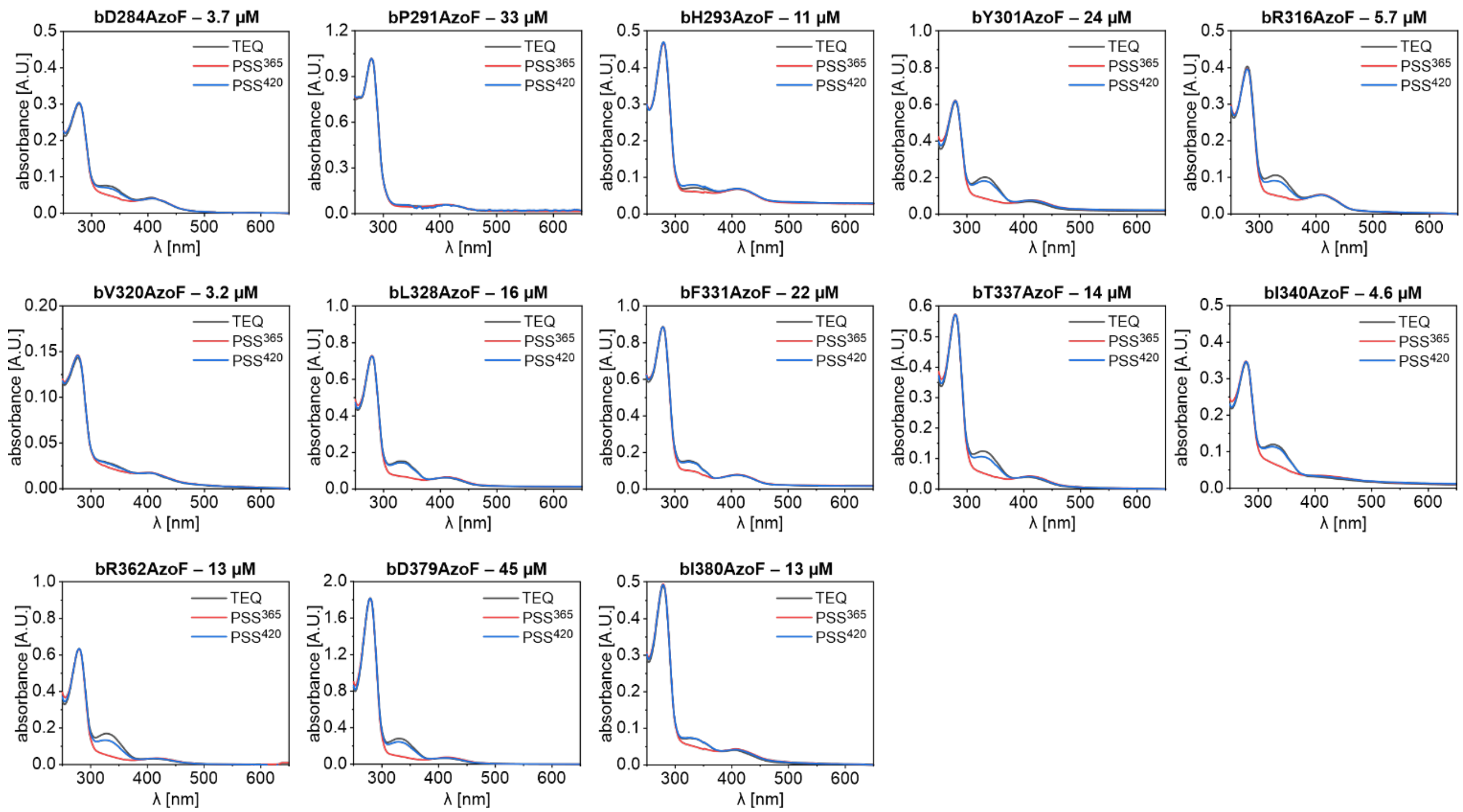

Figure S13. Absorbance spectra of purified AzoF-containing TrpB variants in TEQ, PSS^{365}, and PSS^{420}. Due to the higher extinction coefficient of TrpB itself, the relative intensity of the AzoF signal compared to the protein absorbance was generally lower. Additionally, the PLP cofactor contributes to the absorbance at ~420 nm in addition to AzoF.[4] The majority of variants showed more or less pronounced signals at ~330 nm (π–π* transition) and ~430 nm (n–π* transition). Intensity differences in AzoF absorbance result from different amounts of impurities as well as from quenching effects caused by the local enzyme environment. AzoF incorporation and switching could not be confirmed, possibly because of such effects, for bY26AzoF, bY69AzoF, bH77AzoF, bK98AzoF, bL101AzoF, bW172AzoF and bP291AzoF. *Reaction conditions*: protein in 50 mM KP pH 7.5, 100 mM KCl (concentrations given above each panel) in its TEQ and after 2 s 365 nm as well as 7 s 420 nm irradiation (**Table S3**).

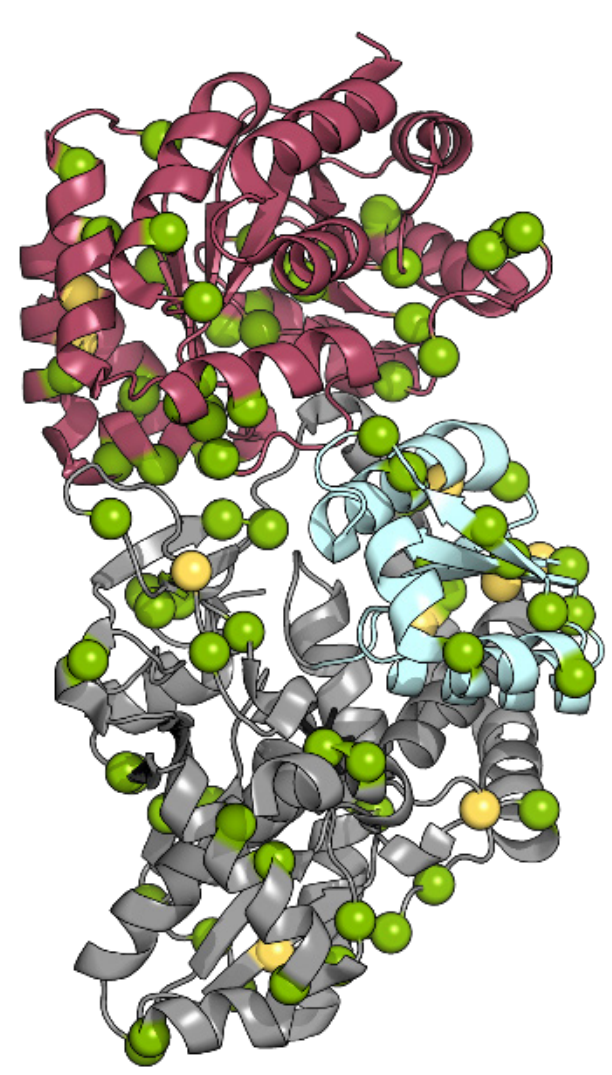

Figure S14. Distribution of incorporation positions (yellow spheres) for which AzoF incorporation in the respective variants could not be confirmed via UV/Vis (see **Figure S12** and **Figure S13**) in comparison to those, in which AzoF could be incorporated (green spheres). Red: TrpA; grey: TrpB; lightblue: COMM domain.

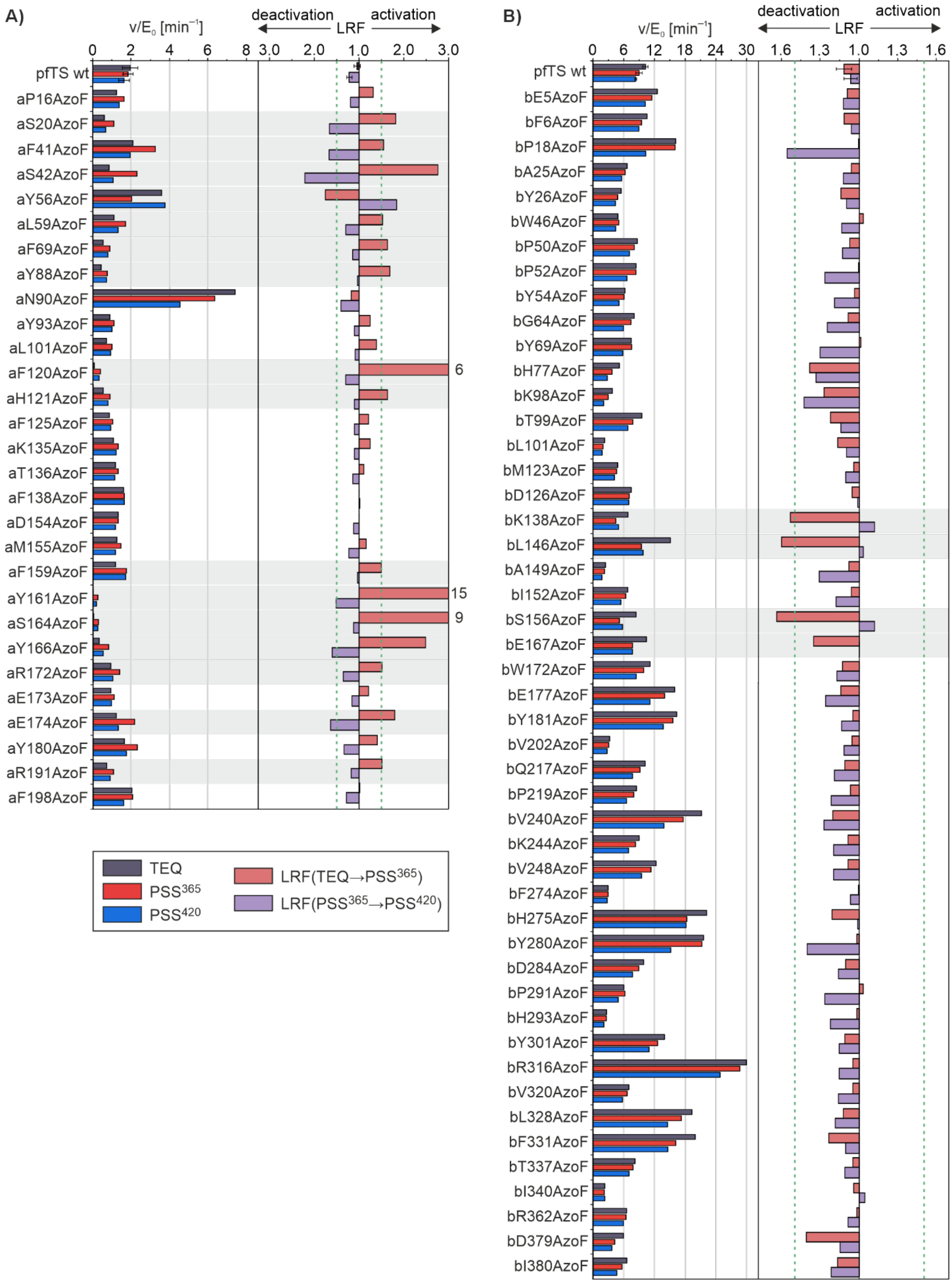
A)
v/E₀ [min⁻¹]
deactivation
activation
LRF
pfTS wt
aP16AzoF
aS20AzoF
aF41AzoF
aS42AzoF
aY56AzoF
aL59AzoF
aF69AzoF
aY88AzoF
aN90AzoF
aY93AzoF
aL101AzoF
aF120AzoF
aH121AzoF
aF125AzoF
aK135AzoF
aT136AzoF
aF138AzoF
aD154AzoF
aM155AzoF
aF159AzoF
aY161AzoF
aS164AzoF
aY166AzoF
aR172AzoF
aE173AzoF
aE174AzoF
aY180AzoF
aR191AzoF
aF198AzoF
6
15
9
TEQ
PSS365
PSS420
LRF(TEQ→PSS365)
LRF(PSS365→PSS420)
B)
v/E₀ [min⁻¹]
deactivation
activation
LRF
pfTS wt
bE5AzoF
bF6AzoF
bP18AzoF
bA25AzoF
bY26AzoF
bW46AzoF
bP50AzoF
bP52AzoF
bY54AzoF
bG64AzoF
bY69AzoF
bH77AzoF
bK98AzoF
bT99AzoF
bL101AzoF
bM123AzoF
bD126AzoF
bK138AzoF
bL146AzoF
bA149AzoF
bI152AzoF
bS156AzoF
bE167AzoF
bW172AzoF
bE177AzoF
bY181AzoF
bV202AzoF
bQ217AzoF
bP219AzoF
bV240AzoF
bK244AzoF
bV248AzoF
bF274AzoF
bH275AzoF
bY280AzoF
bD284AzoF
bP291AzoF
bH293AzoF
bY301AzoF
bR316AzoF
bV320AzoF
bL328AzoF
bF331AzoF
bT337AzoF
bI340AzoF
bR362AzoF
bD379AzoF
bI380AzoF

Figure S15. Screening of TrpA (A) and TrpB (B) variants for their photocontrol potential. Left Columns show the fitted catalytic rate normalized to the enzyme concentration (*v/E₀*) in TEQ (grey), PSS^{365} (red), and PSS^{420} (blue). Activity values were thereby obtained from real-time photocontrol measurements (cf. **Figure S9**). Right columns show LRF(TEQ→PSS^{365}) and LRF(PSS^{365}→PSS^{420}) calculated from the ratio of activities (v_1/v_2 with $v_1 > v_2$) between TEQ and PSS^{365} (salmon) or PSS^{365} and PSS^{420} (lavender), respectively. Numbers next to the bar graph indicate LRF values outside the given range. Green dotted lines designate a significant LRF of ≥ 1.5. Shaded areas indicate TrpA and TrpB variants with LRFs ≥ 1.5. *TrpA reaction conditions*: 80 µM PLP, 6 mM NAD$^+$, 20 mM Na_3AsO_4, 5 µM GAPDH, >250 µM IGP (saturated) and 1 µM TS in 50 mM KP pH 7.5, 100 mM KCl at 50 °C. *TrpB reaction conditions*: 40 mM L-serine, 250 µM indole, 40 µM PLP and 1 µM TS in 50 mM KP pH 7.5, 100 mM KCl at 50 °C. The reactions were followed first in TEQ and then after 2 s irradiation with 365 nm to obtain PSS^{365} and subsequently after 7 s irradiation with 420 nm to obtain PSS^{420} (**Table S3**).

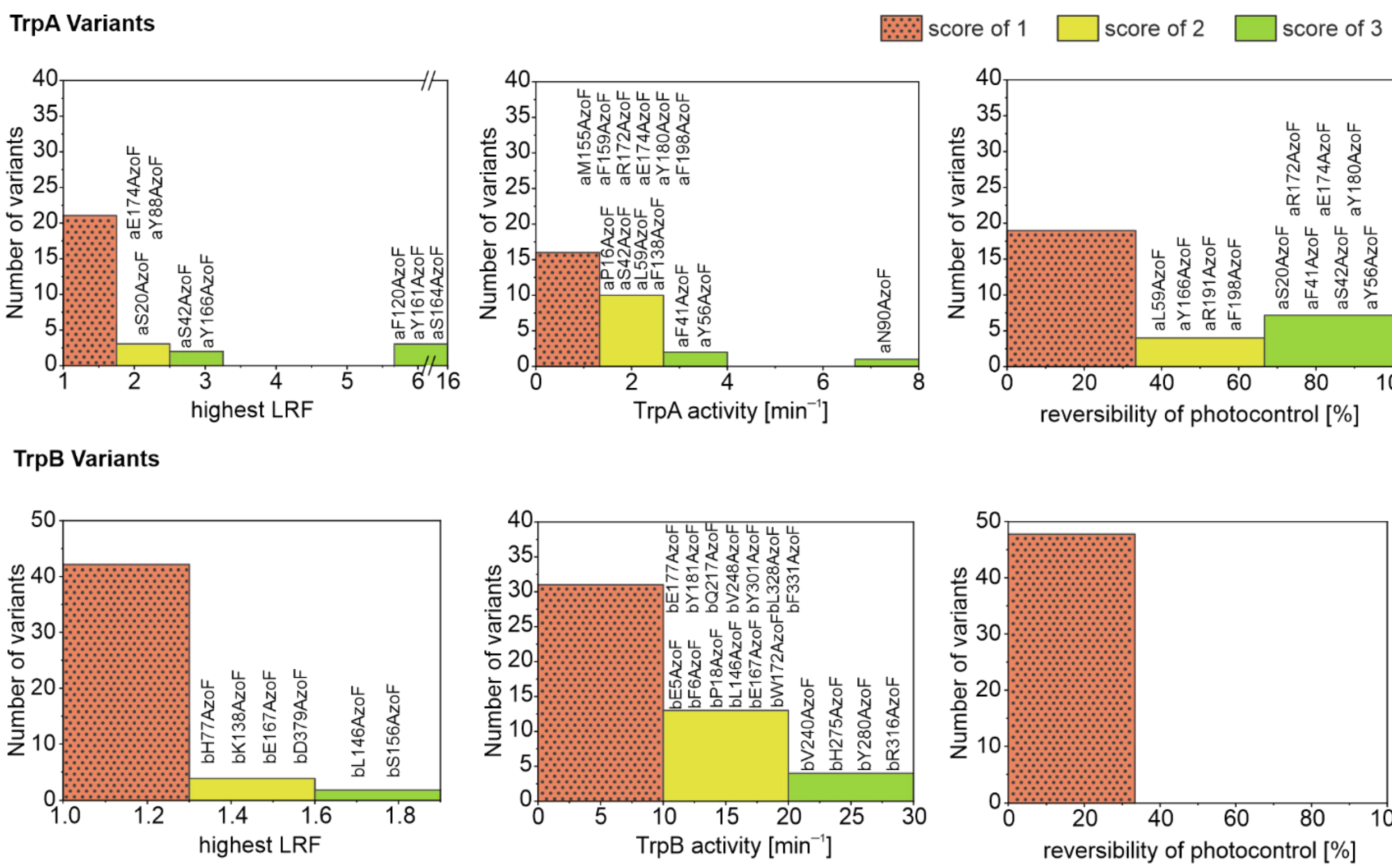

Figure S16. Classification of TrpA and TrpB variants according to their highest LRF (left panel), their highest activity, either in TEQ, PSS^{365} or PSS^{420} (middle panel), and their reversibility of photocontrol as calculated using **Equation 3** (right panel). The values for all variants were grouped into three categories that were assigned to scores from 1 to 3 (orange, yellow, green), with a score of 3 representing variants with the highest LRF, the most pronounced reversibility, or the highest activity.

Table S4. Classification of TrpA-AzoF variants according to their LRF, TrpA activity, and reversibility.

TrpA Variant	LRF score[a]	TrpA activity score[a]	reversibility score[a]	overall score[b]
aP16AzoF	1	2	2	4
aS20AzoF	2	1	3	6
aF41AzoF	1	3	3	9
aS42AzoF	3	2	3	18
aY56AzoF	1	3	3	9
aL59AzoF	1	2	2	4
aF69AzoF	1	1	1	1
aY88AzoF	2	1	1	2
aN90AzoF	1	3	1	3
aY93AzoF	1	1	1	1
aL101AzoF	1	1	1	1
aF120AzoF	3	1	1	3
aH121AzoF	1	1	1	1
aF125AzoF	1	1	1	1
aK135AzoF	1	1	1	1
aT136AzoF	1	1	1	1
aF138AzoF	1	2	1	2
aD154AzoF	1	1	1	1
aM155AzoF	1	2	1	2
aF159AzoF	1	2	1	2
aY161AzoF	3	1	1	3
aS164AzoF	3	1	1	3
aY166AzoF	3	1	2	6
aR172AzoF	1	2	3	6
aE173AzoF	1	1	1	1
aE174AzoF	2	2	3	12
aY180AzoF	1	2	3	6
aR191AzoF	1	1	1	1
aF198AzoF	1	2	1	2

[a]Scores of each variant were obtained from the categorization shown in **Figure S14**. [b]Overall scores were obtained by multiplying the single scores for LRF, activity and reversibility. Highlighted values designate potential photoxenases that were further investigated. Note that this scoring system filtered out variants, which excelled in only one category, such as aF120AzoF, which exhibited an exceptionally high LRF value despite low activity, or aN90AzoF, which showed significantly elevated activity.

Table S5. Classification of TrpB-AzoF variants according to their LRF, TrpB activity and reversibility.

TrpB Variant	LRF score[a]	TrpB activity score[a]	reversibility score[a]	overall score[b]
bE5AzoF	1	2	1	2
bF6AzoF	1	2	1	2
bP18AzoF	1	2	1	2
bA25AzoF	1	1	1	1
bY26AzoF	1	1	1	1
bW46AzoF	1	1	1	1
bP50AzoF	1	1	1	1
bP52AzoF	1	1	1	1
bY54AzoF	1	1	1	1
bG64AzoF	1	1	1	1
bY69AzoF	1	1	1	1
bH77AzoF	2	1	1	2
bK98AzoF	1	1	1	1
bT99AzoF	1	1	1	1
bL101AzoF	1	1	1	1
bM123AzoF	1	1	1	1
bD126AzoF	1	1	1	1
bK138AzoF	2	1	1	2
bL146AzoF	3	2	1	6
bA149AzoF	1	1	1	1
bI152AzoF	1	1	1	1
bS156AzoF	3	1	1	3
bE167AzoF	2	2	1	4
bW172AzoF	1	2	1	2
bE177AzoF	1	2	1	2
bY181AzoF	1	2	1	2
bV202AzoF	1	1	1	1
bQ217AzoF	1	2	1	2
bP219AzoF	1	1	1	1
bV240AzoF	1	3	1	3
bK244AzoF	1	1	1	1
bV248AzoF	1	2	1	2
bF274AzoF	1	1	1	1
bH275AzoF	1	3	1	3
bY280AzoF	1	3	1	3
bD284AzoF	1	1	1	1
bP291AzoF	1	1	1	1
bH293AzoF	1	1	1	1
bY301AzoF	1	2	1	2
bR316AzoF	1	3	1	3
bV320AzoF	1	1	1	1
bL328AzoF	1	2	1	2
bF331AzoF	1	2	1	2
bT337AzoF	1	1	1	1
bI340AzoF	1	1	1	1

bR362AzoF	1	1	1	1
bD379AzoF	2	1	1	2
bI380AzoF	1	1	1	1

[a]Scores of each variant were obtained from the categorization shown in **Figure S14**. [b]Overall scores were obtained by multiplying the single scores for LRF, activity and reversibility. Highlighted values designate potential photoxenases that were further investigated.

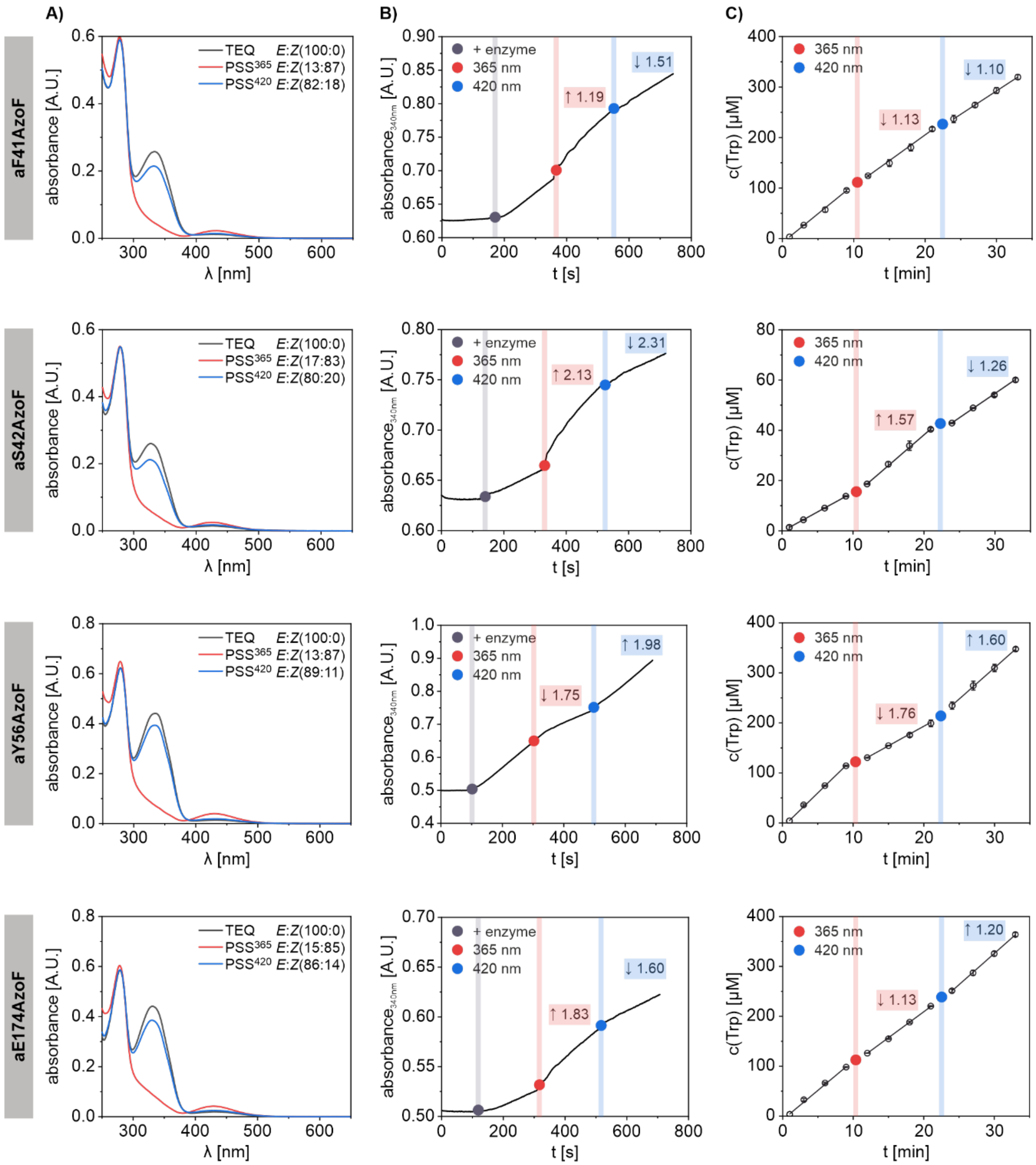

Figure S17. Characterization of the selected TrpA variants aF41AzoF, aS42AzoF, aY56AzoF, and aE174AzoF. A) Absorbance spectra of 30 µM protein in 50 mM KP pH 7.5, 100 mM KCl in its TEQ, PSS^{365} and PSS^{420} after irradiation with 2 s 365 nm and 7 s 420 nm (**Table S3**). The *E*:*Z* isomer distributions were estimated for each state as described previously.[5,6] B) Real-time photocontrol of TrpA activity monitored by continuous spectrophotometric detection of GAP emergence. Exemplary data of one replicate are shown and the mean LRF(TEQ→PSS^{365}) (red) and LRF(PSS^{365}→PSS^{420}) (blue) values of technical triplicates are given with arrows indicating a light-induced activation (↑) or deactivation (↓) (c.f. **Figure 4**). *TrpA Reaction conditions*: 80 µM PLP, 6 mM NAD^+, 20 mM Na_3AsO_4, 5 µM GAPDH, >250 µM IGP (saturated) and 1 µM TS in 50 mM KP pH 7.5, 100 mM KCl at 50 °C. C) Real-time photocontrol of TS activity by discontinuous detection of tryptophan via HPLC. Triplicate data points (black) with error bars indicating the standard deviation (S.D.) and the mean LRF(TEQ→PSS^{365}) (red) and LRF(PSS^{365}→PSS^{420}) (blue) values are shown. *TS Reaction conditions*: 40 mM L-serine, 250 µM indole, and 40 µM PLP in 50 mM KP pH 7.5, 100 mM KCl at 50 °C. B,C) Dots indicate specific time points where the enzyme complex was added (grey), and where irradiation was applied for 2 s at 365 nm (red) or 7 s at 420 nm (blue; **Table S3**). In the TS reaction, the enzyme was added at timepoint t = 0.

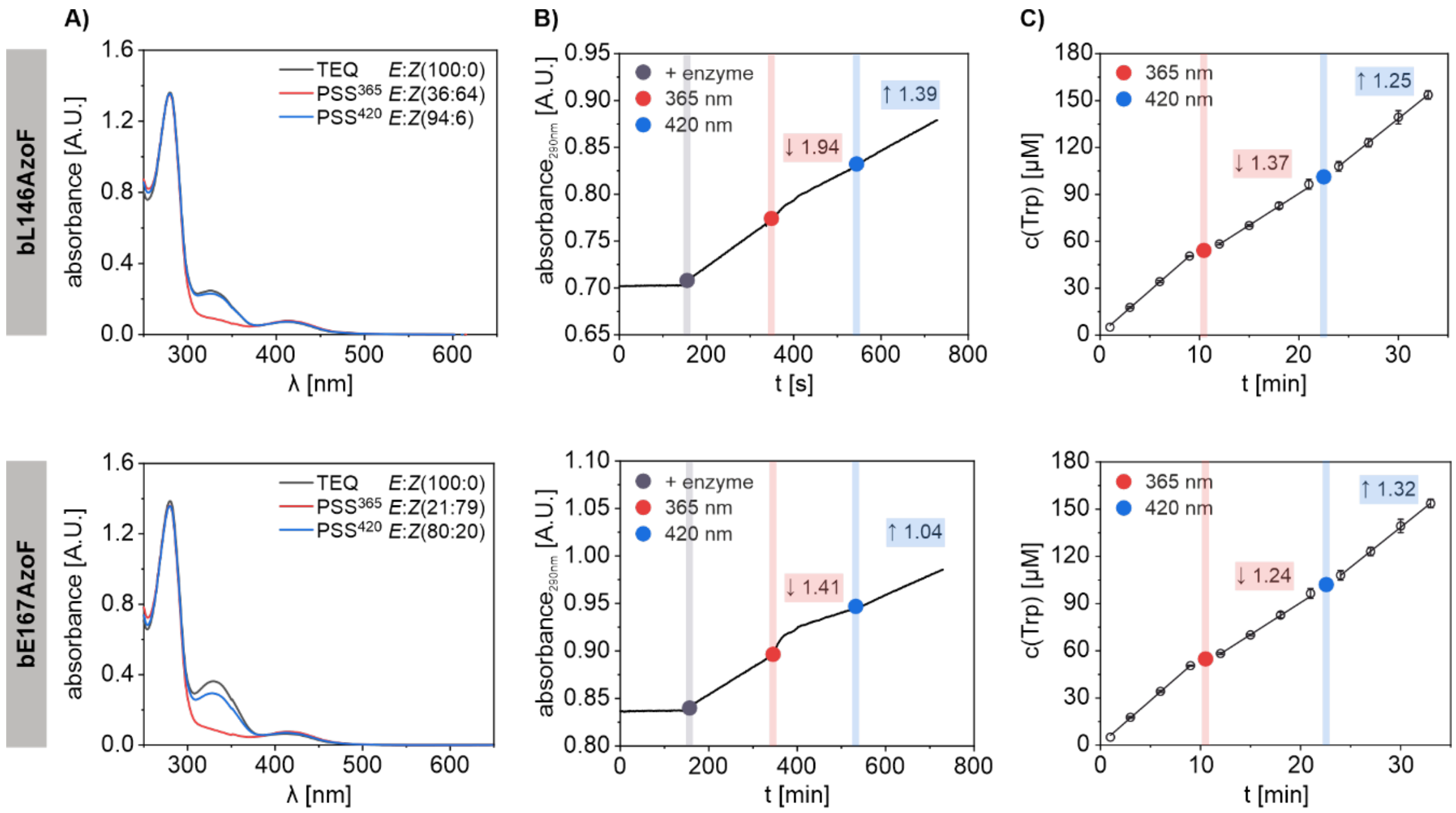

Figure S18. Characterization of the selected TrpB variants bL146AzoF and bE167AzoF. A) Absorbance spectra of 30 µM protein in 50 mM KP pH 7.5, 100 mM KCl in its TEQ, PSS^{365} and PSS^{420} after irradiation with 2 s 365 nm and 7 s 420 nm (**Table S3**). The *E*:*Z* isomer distributions were estimated for each state as described previously.[5,6] B) Real-time photocontrol of TrpB activity monitored by continuous spectrophotometric detection of L-tryptophan emergence. Exemplary data of one replicate are shown and the mean LRF(TEQ→PSS^{365}) (red) and LRF(PSS^{365}→PSS^{420}) (blue) values of technical triplicates are given with arrows indicating a light-induced activation (↑) or deactivation (↓) (c.f. **Figure 4**). *TrpB reaction conditions*: 40 mM L-serine, 250 µM indole, 40 µM PLP and 1 µM TS in 50 mM KP pH 7.5, 100 mM KCl at 50 °C. C) Real-time photocontrol of TS activity by discontinuous detection of tryptophan via HPLC. Triplicate data points (black) with error bars indicating the S.D. and the mean LRF(TEQ→PSS^{365}) (red) and LRF(PSS^{365}→PSS^{420}) (blue) values are shown. *TS Reaction conditions:* 40 mM L-serine, 250 µM indole, and 40 µM PLP in 50 mM KP pH 7.5, 100 mM KCl at 50 °C. B,C) Dots indicate specific time points where the enzyme complex was added (grey), and where irradiation was applied for 2 s at 365 nm (red) or 7 s at 420 nm (blue; **Table S3**). In the TS reaction, the enzyme was added at timepoint t = 0.

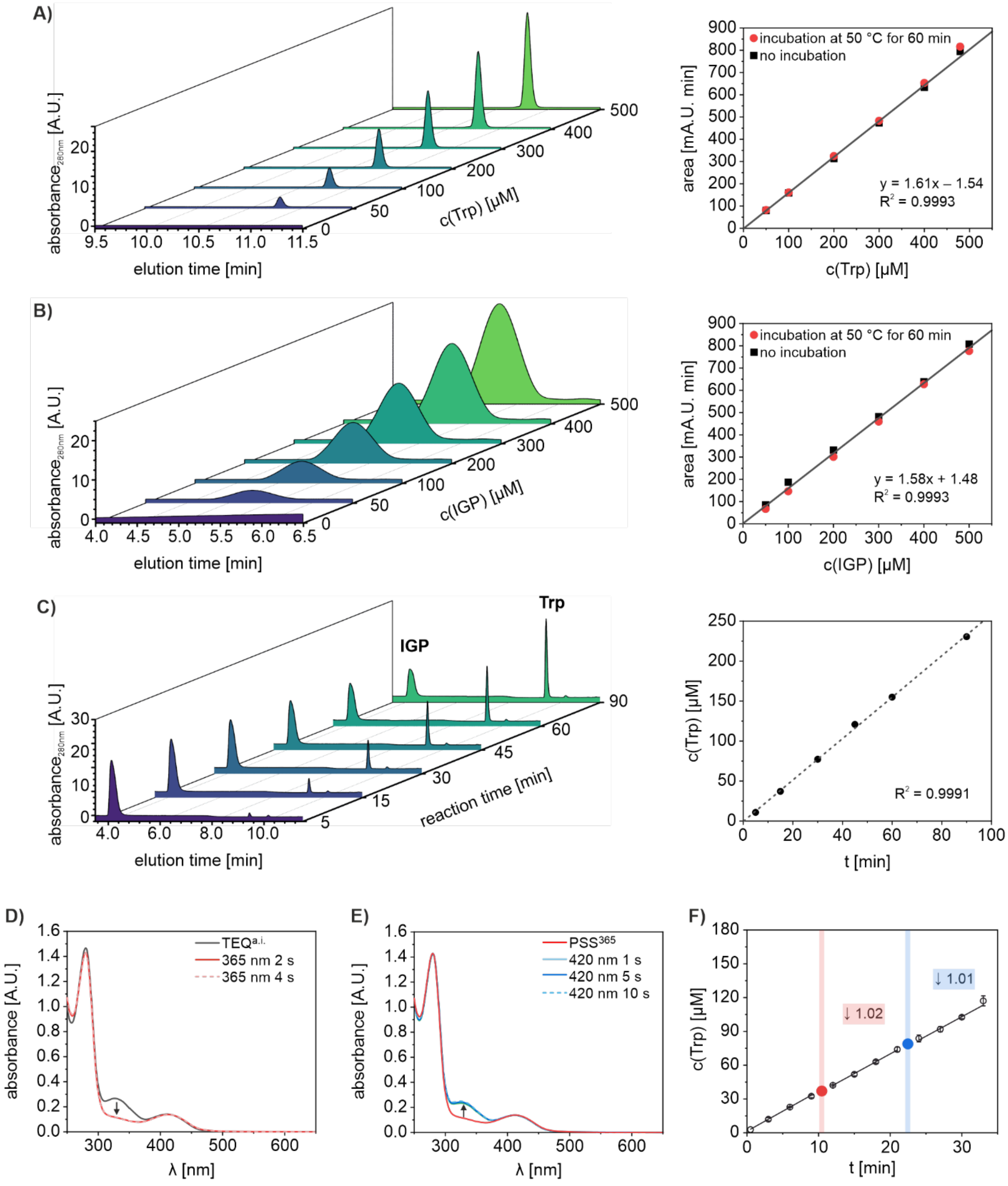

Figure S19. Pre-tests to examine the photocontrol of TS activity by a discontinuous chromatographic detection assay. A,B) Correlation between HPLC peak areas and L-tryptophan (Trp; A) or IGP (B) concentrations. Trp and IGP samples were applied to HPLC either directly without incubation (right subpanel, black) or after incubation at 50 °C for 60 min (left subpanel; right subpanel, red). The latter showed no deviations indicative of substrate or product degradation. C) Trp detection (left supanel) following the TS reaction of 2 µM wt-TS at 50 C° demonstrated a linearity of activity over the whole measurement range (right subpanel). Reaction conditions: 500 µM IGP, 10 mM L-serine, and 40 µM PLP in 50 mM KP pH 7.5, 100 mM CsCl. D,E) Determination of irradiation durations via UV/Vis spectroscopy required to completely establish PSS^{365} (left) and PSS^{420} (right) in a 1.5 mL reaction tube (LED positioned directly above the tube). PSS^{365} was reached after 2 s of 365 nm irradiation and PSS^{420} after 5 s of 420 nm irradiation. Similar to the previously determined irradiation durations in a quartz cuvette of 1 cm depth (**Figure S8**), 2 s 365 nm and 7 s 420 nm irradiation durations were chosen for subsequent real-time photocontrol experiments. *Reaction conditions*: 40 µM bY10AzoF

(50 mM KP pH 7.5, 100 mM KCl) in TEQ and after 365 nm and 420 nm irradiation (**Table S3**). F) Real-time photocontrol of TS activity of 1 µM wt-TS. Reaction conditions were the same as in (C). Dots indicate specific time points where irradiation was applied for 2 s at 365 nm (red) or 7 s at 420 nm (blue). The enzyme was added at timepoint t = 0. LRF(TEQ→PSS^{365}) and LRF(PSS^{365}→PSS^{420}) were calculated from the ratio of activities (v_1/v_2 with $v_1 > v_2$) between TEQ and PSS^{365} (red) or PSS^{365} and PSS^{420} (blue).

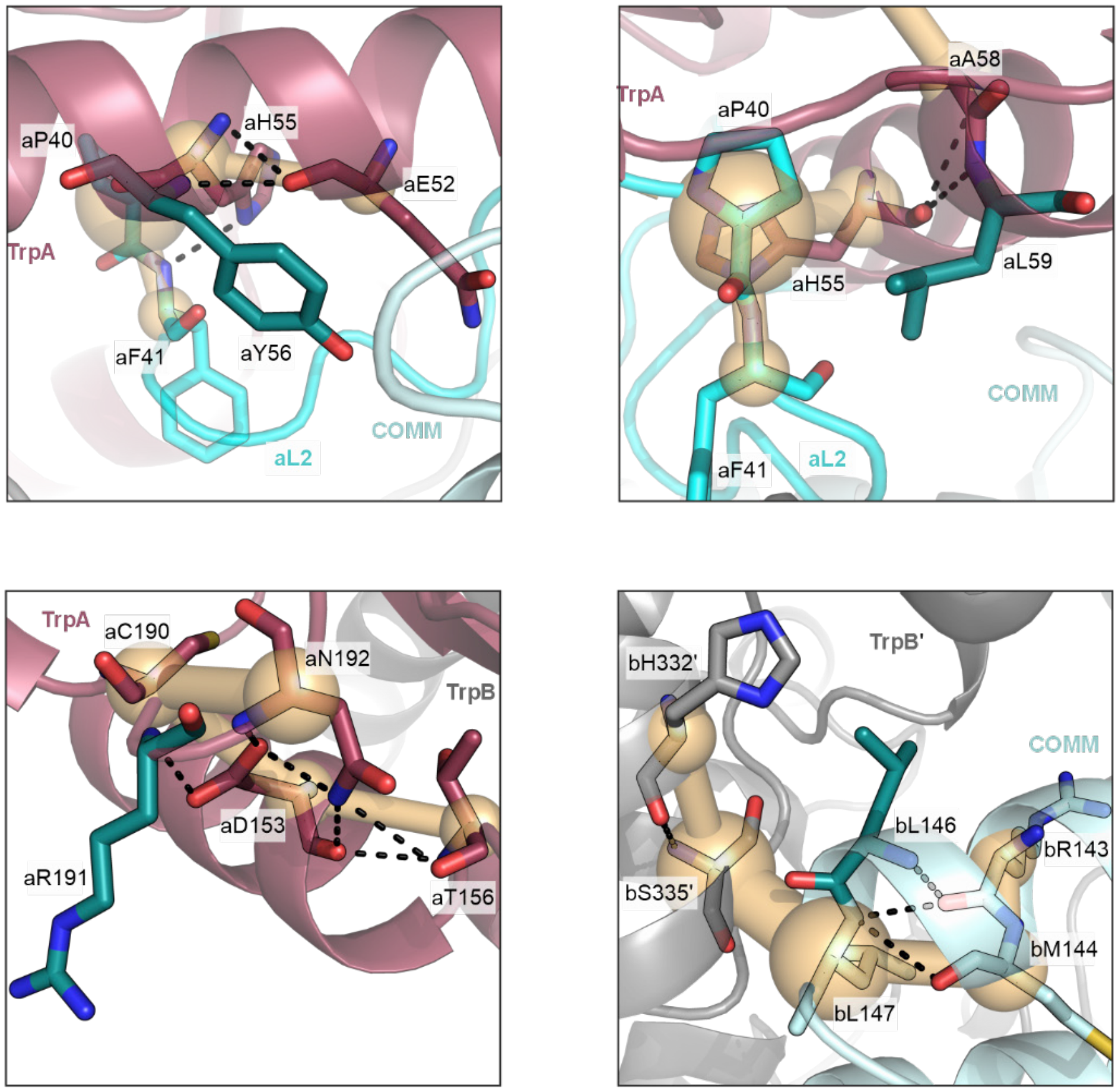

Figure S20. Interactions of the four effective AzoF incorporation sites aY56, aL59, aR191 and bL146 that are near (< 5 Å) an SPM position with the closest SPM residues and facilitate an LRF >1.5. In each case, only backbone interactions are established.

Chromatograms

Fragmentor Voltage 120 **Collision Energy** 0 **Ionization Mode** ESI

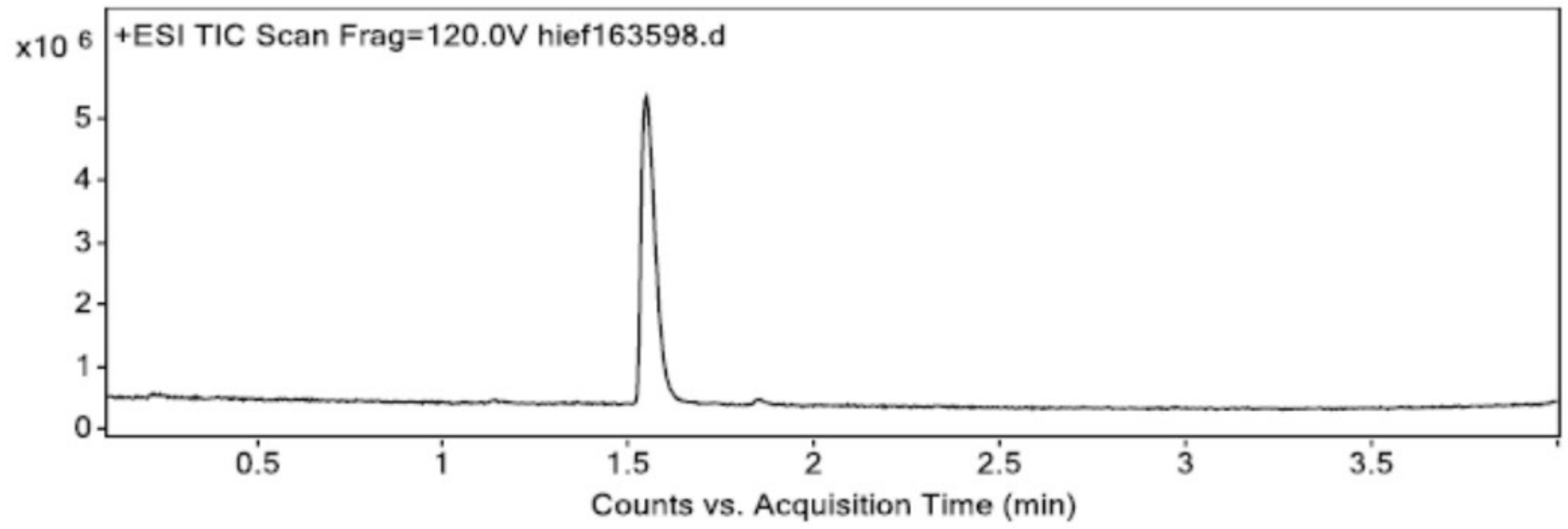

Fragmentor Voltage 120 **Collision Energy** 0 **Ionization Mode** ESI

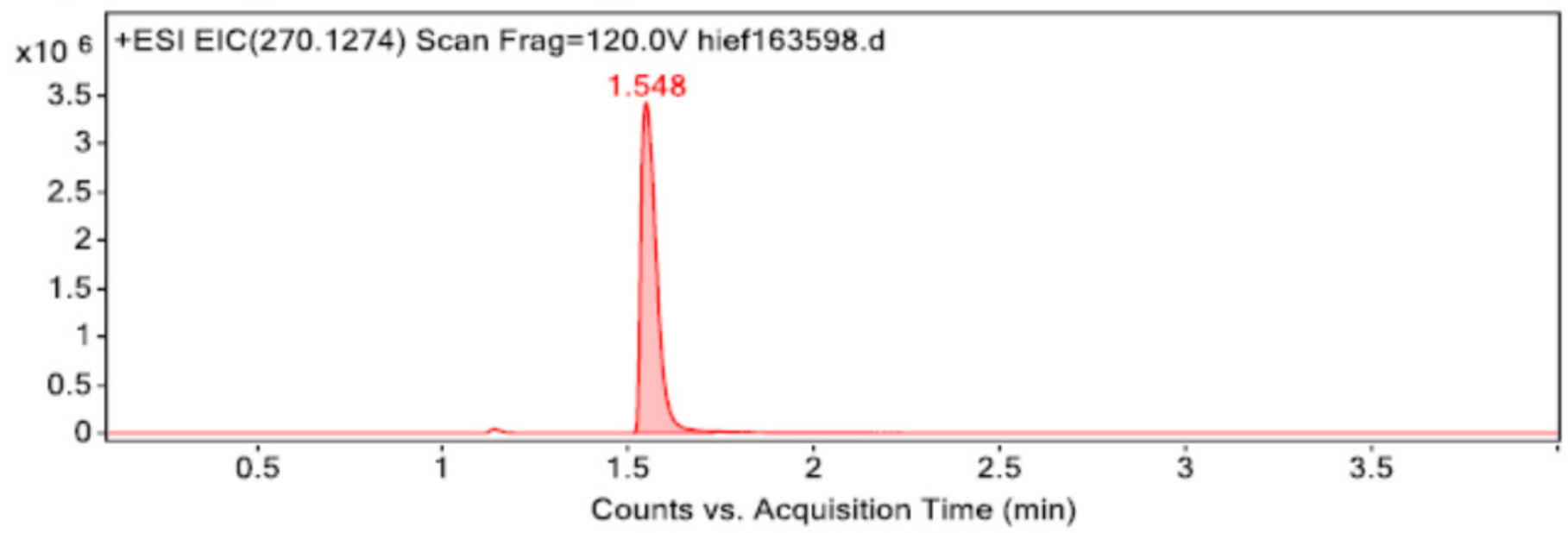

Integration Peak List

Peak	Start	RT	End	Height	Area	Area %
1	1.513	1.548	1.727	3419918.69	9935702.12	100

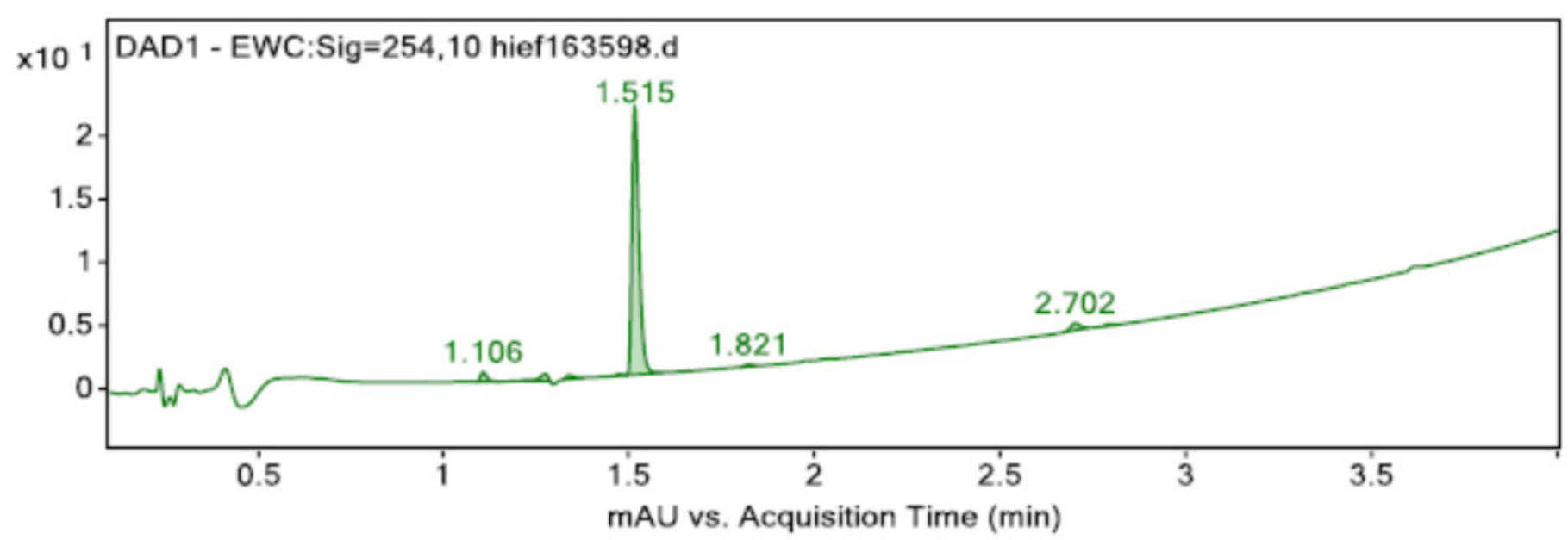

Figure S21. High resolution mass spectrometry of AzoF confirmed its identity and purity.

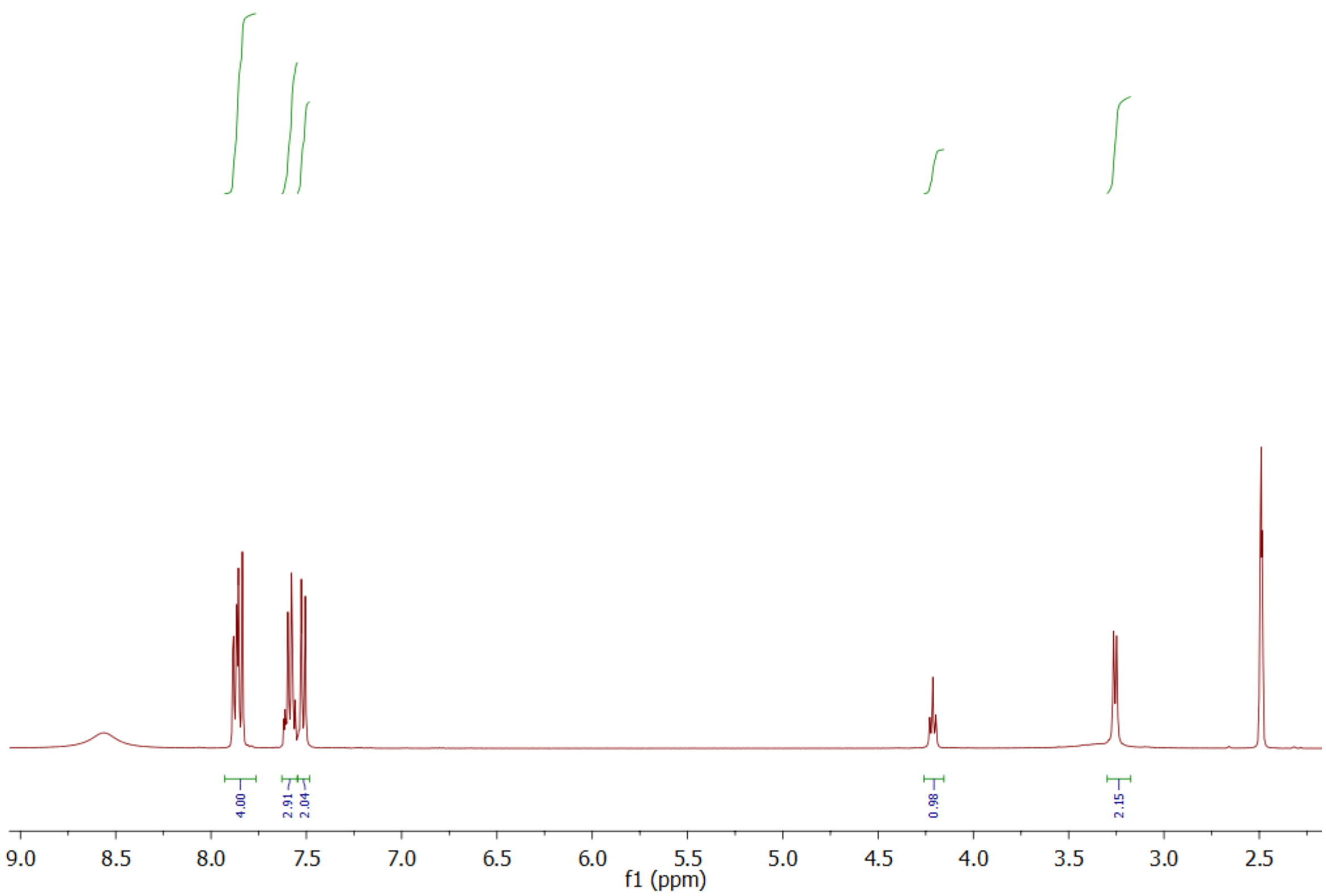

Figure S22.^{1}H-NMR spectrum of AzoF confirmed its identity and purity.

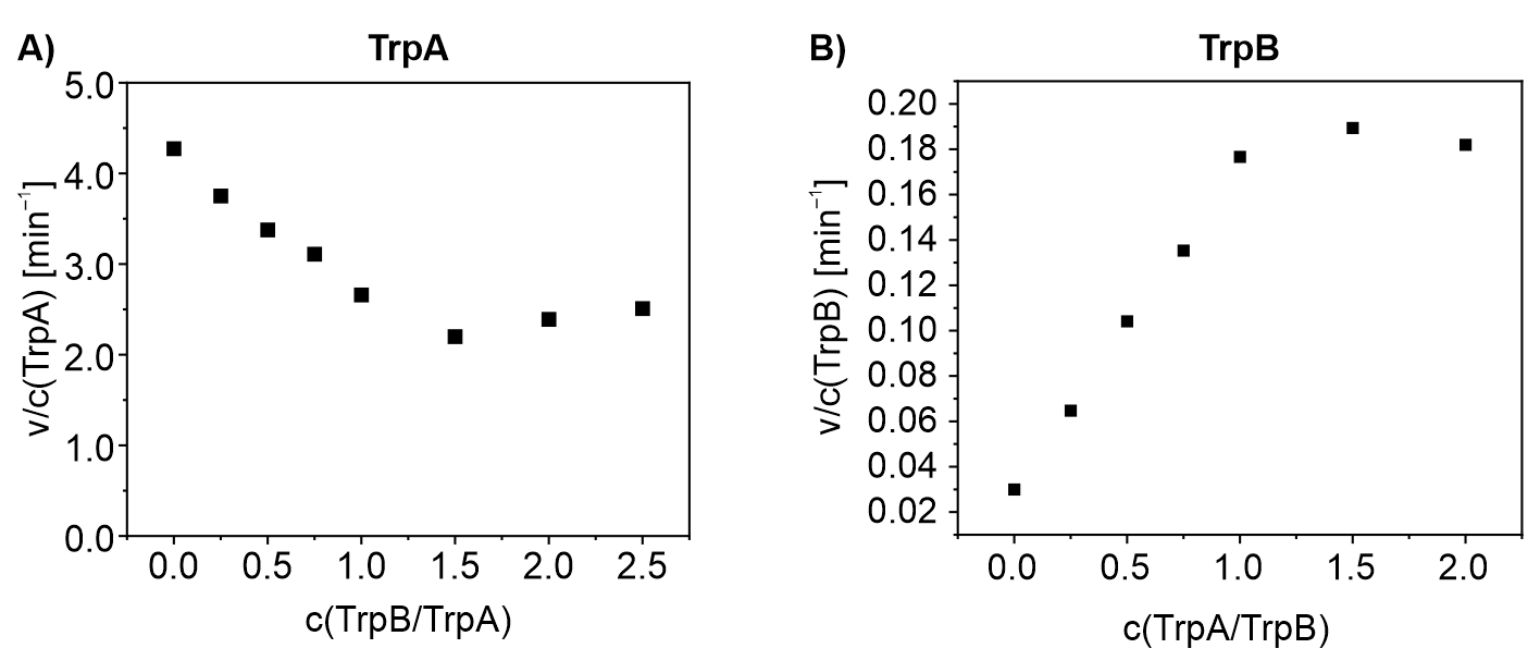

Figure S23. Activity titration experiments to determine the excess of wt-TrpA or wt-TrpB required to reach full complex formation. A) wt-TrpA (1 µM) was assayed with varying concentrations of wt-TrpB (0–2.5 µM). Reaction conditions included: 80 µM PLP, 6 mM NAD^+, 20 mM Na_3AsO_4, 5 µM GAPDH, and 250 µM IGP in 50 mM KP pH 7.5, 100 mM KCl at 50°C. B) wt-TrpB (1 µM) was assayed with varying concentrations of wt-TrpA (0–2 µM). Reaction conditions included: 40 mM L-serine, 250 µM indole, and 40 µM PLP in 50 mM KP pH 7.5, 100 mM KCl at 50 °C. All reactions were performed as single measurements.

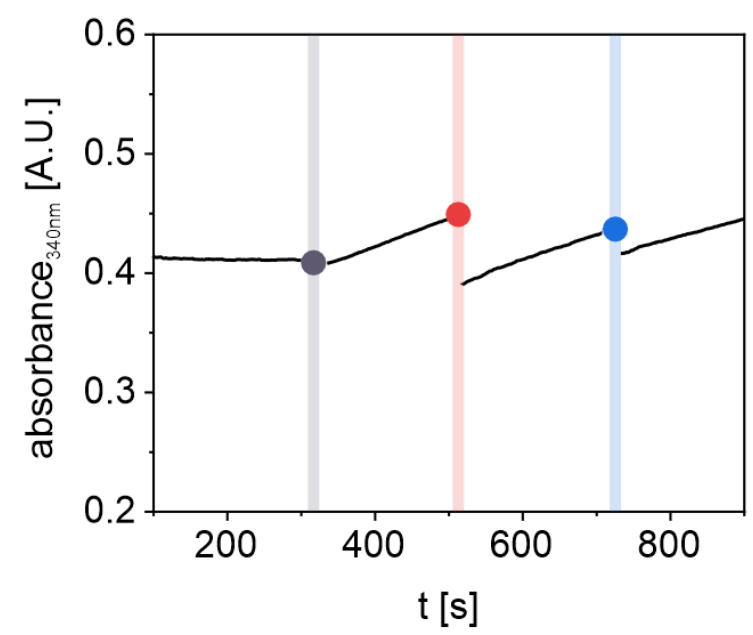

Figure S24. Real-time photocontrol of TrpA activity in wt-TS (1 µM) to validate the integrity of the enzyme itself and the assay conditions under irradiation. Dots indicate specific time points where the enzyme was added (grey), and where irradiation was applied for 2 s at 365 nm (red) or 7 s at 420 nm (blue; **Table S3**). After irradiation with both 365 nm and 420 nm, a drop in absorbance is noticeable, implying photodamage of NAD^+. Nevertheless, NAD^+ is still present in saturating concentrations. The drop in absorbance was corrected for all TrpA reactions.

2. Experimental section

2.1 Synthesis of phenylalanine-4'-azobenzene (AzoF)

AzoF was synthesized according to a previously published protocol (**Scheme S1**)[8] and its integrity and purity were confirmed via high resolution mass spectrometry and ^{1}H-NMR-spectroscopy (**Figure S21,S22**). In a first step, 4-amino-*N*-Boc-l-phenylalanine **1** (2.5 g, 8.95 mmol, 1.0 eq.) was dissolved in glacial acetic acid (50 mL). The flask was then covered with aluminum foil and nitrosobenzene **2** (955 mg, 8.93 mmol, 1.0 eq.) was added. After being stirred at room temperature for 24 h, the reaction mixture was quenched by being poured onto crushed ice/water (500 mL) and extracted with ethyl acetate (3 × 100 mL). The combined organic layers were washed with water (2 × 50 mL) and brine (1 × 50 mL) and were subsequently dried with Na_2SO_4. The volatiles were removed *in vacuo,* and the crude product was purified by an automated flash column chromatography (Biotage Selekt, Biotage) in DCM with a linear gradient of methanol (0–10%) yielding intermediate **3**, which was directly used in the next step.

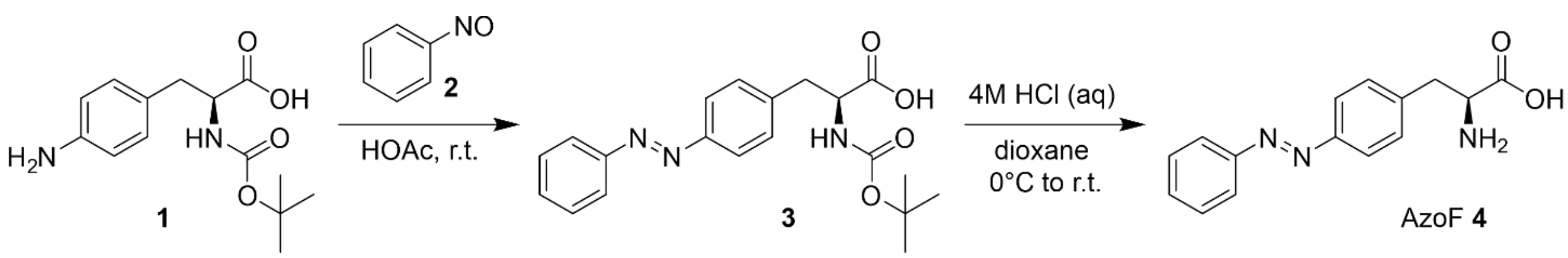

Scheme S1. Synthesis of phenylalanine-4'-azobenzene (AzoF).

To obtain AzoF (**4**), intermediate **3** (1.8 g, 6.44 mmol) was dissolved in dioxane (20 mL) at 0 °C. Then, 4 M HCl (6.6 mL) was added, and the mixture was stirred at room temperature for 24 h. Toluene (50 mL) was added to facilitate the removal of 1,4-dioxane, and the solvent was removed in vacuo. The product was triturated with diethyl ether (4 × 50 mL) and lyophilized overnight, yielding AzoF (overall yield 63%).^{1}H NMR (300 MHz, Chloroform-*d*): δ = 7.97–7.83 (m, 4H), 7.72–7.55 (m, 3H), 7.54–7.43 (m, 2H), 4.22 (t, J = 6.4 Hz, 1H), 3.25 (t, J = 6.8 Hz, 2H). HRMS (ESI-TOF) m/z calculated for $[C_{15}H_{15}N_3O_2]^+$: 270.12, found: 270.13.

2.2 Synthesis of indole-3-glycerol phosphate (IGP)

IGP was produced enzymatically from 1-(*o*-carboxyphenylamino)-1-deoxyribulose-5-phosphate (CdRP) using indole-3-glycerol phosphate synthase from *Thermotoga maritima* (*tm*TrpC, Uniprot ID: Q56319). To obtain CdRP, 27.4 mg (0.1 mmol) ribose-5-phosphate were dissolved in 0.1 mL H_2O and subsequently mixed with 13.7 mg (0.1 mmol) anthranilate in 0.1 mL ethanol. The mixture was gently stirred and incubated at room temperature for 16–20 h in the dark. Unreacted starting material and byproducts were hydrolyzed by addition of 1.8 mL H_2O and stirring for another hour at room temperature. The aqueous phase containing CdRP was washed ethyl acetate (5 x 5 mL) and remaining volatiles were removed under N_2 gas for 15 min. Subsequently, CdRP was converted into IGP using 10 µM *tm*TrpC in 50 mM HEPES, pH 7.5 at 50 °C. Samples were taken every 15 min and spectrophotometrically analyzed (240–340 nm) to monitor the amount of IGP. After approximately an hour, absorbance at 278 nm was not further increased, indicating complete turnover of CdRP. Subsequently, *tm*TrpC was removed using an Amicon Centrifugal Filter (MWCO: 10 kDa, 4 mL) before the sample (2 mL) was applied to ion exchange chromatography (MonoQ HR 16/10, CV = 20 mL). Equilibration was performed with buffer A: 50 mM $(NH_4)_2CO_3$ pH 8.0 and IGP was eluted using a gradient of 0→100 % buffer B: 1 M $(NH_4)_2CO_3$ pH 8.0. Absorbance spectroscopy (240–340 nm) identified the fractions containing the highest amount of IGP, which were then subsequently pooled and lyophilized for storage at –70 °C. The exact concentration of IGP was determined from the difference in absorbance after total turnover using the TrpA reaction assay (section 2.14) without irradiation.

2.3 Expression and Purification of glyceraldehyde 3-phosphate dehydrogenase (GAPDH) from *Thermotoga maritima*

The auxiliary enzyme glyceraldehyde 3-phosphate dehydrogenase from *Thermotoga maritima* (*tm*GAPDH, Uniprot ID: P17721) was expressed and purified as described in Kneuttinger *et al.*.[10] Briefly, *E. coli* BL21 Gold (DE3) cells were transformed with pET28a_tmGAPDH and grown in 100 mL lysogeny broth (LB) medium supplemented with 75 µg/mL kanamycin (LB_{Kan}) at 37 °C overnight. The next day, 4 L LB_{Kan} were inoculated to an OD_{600} of 0.1 and incubated at 37 °C while shaking until an OD_{600} of 0.6 was reached. Subsequently, gene expression was induced by addition 0.5 mM IPTG and the culture

was incubated at 37 °C overnight. The cells were harvested by centrifugation (4000 rpm, 45 min, 20 °C) and subsequently resuspended in 10 mM KP pH 7.5, 300 mM KCl, 10 mM imidazole. Cells were lysed by sonication (2 min, 30%, 2 s pulse, 2 pause), followed by a heat step of 20 min at 75 °C and another centrifugation step to remove *E. coli* proteins. The lysate was subjected to immobilized metal ion affinity chromatography (HisTrap™ FF Crude column, 5 mL, GE Healthcare) and eluted with a linear gradient of imidazole (10→500 mM). Fractions containing the protein were identified by SDS-PAGE analysis, pooled and dialyzed into 10 mM KP, pH 7.5. The protein was concentrated and dripped into liquid nitrogen for storage at −70 °C.

2.4 Irradiation devices

Irradiation of TS and respective AzoF variants was conducted using a programmable power supply (PeakTech 6225A). The 365 nm high power LED was driven with a voltage of 12 V and a maximum current of 700 mA, while for 420 nm high power LED 9 V and 350 mA were applied (**Table S3**). For sample irradiation, the LEDs were either held directly above the reaction vessel (TS reaction, HPLC measurements) or were attached to cuvette holders directly in front of the LED (spectrophotometric TrpA and TrpB assay). During spectrophotometric measurements, the cuvette was removed from the photometer at a certain timepoint, and irradiation was conducted for 2 s (365 nm) or 7 s (420 nm).

2.5 Multiple sequence alignments

To map the degree of conservation of selected positions in TS, sequence logos were generated from multiple sequence alignment (MSA, **Figure S5–6**). To this end, sequences were extracted from the respective families listed in the InterPro database[11]: IPR002028 for TrpA MSA and IPR006654 for TrpB MSA. Redundant sequences were removed to a final number of <2000 sequences and a MSA was generated using *Clustal O* tool embedded in Jalview.[12] The resulting dataset served as basis to create a sequence logo using WebLogo 3.[13]

2.6 Molecular dynamics simulations-based analyses

Molecular modeling system preparation. The heterocomplex abba starting structure of TS from *Pyrococcus furiosus* was generated with the AlphaFold3 (AF3)[14] neural network, as the available X-ray structure (PDB id.: 1WDW) is not fully resolved. The best ranked

model had the predicted TM score (pTM) of 0.95 and the predicted interface TM score (ipTM) of 0.93. For the heterocomplex MD simulations, the amino acrylate intermediate (A-A) was superposed to same intermediate present in *Salmonella typhimurium* TS X-ray structure (PDB code: 2J9X).

The water molecules added to each subunit were selected from the DBSCAN clusterization[15,16] algorithm implemented in the scikit-learn Python library.[17] For TrpA, the water molecules of different TrpA subunits were used, ordered by PDB accession number and chain: 1TJR_A, 1TJR_B, 1RD5_A, 1RD5_B, 5EY5_A, 5EY5_C, 2DZS_A, 2DZS_B, 2DZU_A, 2DZU_B, 2E09_A, 2E09_B, 2DZW_A, 2DZW_B, 1GEQ_A, 1GEQ_B, 5E0K_A, 5E0K_C, 5E0K_E, 5E0K_G, 5E0K_I, 5E0K_K, 1WDW_A, 1WDW_C, 1WDW_E, 1WDW_G, 1WDW_I, 1WDW_K, 2DZT_A, 2DZT_B, 2DZV_A, 2DZV_B, 2DZP_A, 2DZP_B, 2DZX_A, 2DZX_B. For the heterodimeric MD simulations, the water molecules of the following TrpB subunits were used, ordered by PDB accession number and chain: 5IXJ_A, 5IXJ_B, 5IXJ_C, 5IXJ_D, 5DW3_A, 5DW3_B, 5DW3_C, 5DW3_D, 5E0K_B, 5E0K_D, 5E0K_F, 5E0K_H, 5E0K_J, 5E0K_L, 5DW0_A, 5DW0_B, 5DW0_C, 5DW0_D, 1V8Z_A, 1V8Z_B, 1V8Z_C, 1V8Z_D, 5T6M_A, 5T6M_B, 5T6M_C, 5T6M_D, 1WDW_B, 1WDW_D, 1WDW_F, 1WDW_H, 1WDW_J, 1WDW_L, 5DVZ_A, 5DVZ_B, 5DVZ_C, 5DVZ_D, 6AMH_A, 6AMH_B, 6AMH_C, 6AMH_D, 6AMI_A, 6AMI_B, 6AMI_C, 6AMI_D, 6AMC_A, 6AMC_B, 6AMC_C, 6AMC_D, 5VM5_A, 5VM5_B, 5VM5_C, 5VM5_D, 6AM8_A, 6AM8_B, 6AM8_C, 6AM8_D, 6AM9_A, 6AM9_B, 6AM9_C, 6AM9_D, 6AM7_A, 6AM7_B, 6AM7_C, 6AM7_D, 6CUV_A, 6CUV_B, 6CUV_C, 6CUV_D, 6CUT_A, 6CUT_B, 6CUT_C, 6CUT_D, 6CUZ_A, 6CUZ_B, 6CUZ_C, 6CUZ_D, 5EY5_B, 5EY5_D. Additionally, one conserved sodium ion in the X-ray structure was added to the TrpB subunit located close to the active site.

The MD parameters for the A-A intermediate were generated with the antechamber and parmchk2 modules of AMBER20[18] using the 2nd generation of the general amber force-field (GAFF2).[18,19] The A-A intermediate was optimized at the B3LYP/6-31G(d) level of theory including Grimme's dispersion correction with Becke-Johnson Damping (D3-BJ) and the polarizable conductor model (PCM) (diethyl ether, $\varepsilon = 4.2$) as an estimation of the dielectric permittivity in the enzyme active site.[20] The partial charges (RESP model)[21] were set to fit the electrostatic potential generated at the HF/6-31G(d) level of theory. The

charges were calculated according to the Merz-Singh-Kollman[22] scheme using the Gaussian16[23] software package. The protonation states were predicted using PROPKA.[24,25] For TrpA the protonation state of the catalytic residue Glu49/50 was neutral (i.e., GLH49 and GLH50), as is described in the TrpA mechanism. Two protonation states of the TrpB catalytic residue Lys84 were used: the neutral (*i.e.*, LYN84) and the protonated (*i.e.*, LYS84).[26] The enzyme structures were solvated in a pre-equilibrated truncated octahedral box of 10 Å edge distance using the OPC water model and neutralized by the addition of explicit counterions (i.e., Na^+) using the AMBER20 leap module. All MD simulations were performed using a modification of the amber99 force field (ff19SB).[27]

MD simulation details. MD equilibration phase was done following the protocol described by Roe and Brooks with small differences fine-tuned to our systems.[28] The bonds involving hydrogen are constrained by SHAKE algorithm during the non-minimization steps. Long-range electrostatic effects were modeled using the particle mesh-Ewald method.[29] For Lennard–Jones and electrostatic interactions, a 10 Å cut-off was applied. The MD protocol starts with the minimization phase of 1500 steps of the steepest descent method followed by 3500 steps of the conjugate gradient method with a positional restrain (*i.e.*, force constant of 5.0 $kcal \cdot mol^{-1} \cdot Å^{-2}$) to the protein heavy atoms. The following heating phase a temperature increment from 25 K to 300 K during 20 ps of MD simulation time, a Langevin thermostat with a collision frequency of 5 ps^{-1}, and a positional restrain (i.e., force constant of 5.0 $kcal \cdot mol^{-1} \cdot Å^{-2}$) to the protein heavy atoms are performed. A minimization and heating of all atoms in the system is the following step. Starting with two minimization stages of 1000 steps of the steepest descent method followed by 1500 steps of the conjugate gradient method each with a positional restrain (i.e., force constant of 2.0 $kcal \cdot mol^{-1} \cdot Å^{-2}$ in the first minimization and 0.1 $kcal \cdot mol^{-1} \cdot Å^{-2}$ in the second) to the protein heavy atoms. Following, a third minimization phase of 1500 steps of the steepest descent method followed by 3500 steps of the conjugate gradient method without any positional restraint is performed. The system is then heated in accordance with the previously established procedure. Finally, a five-round equilibration phase at the NPT ensemble with a constant pressure of 1 atm is performed. The first four rounds were done with the Berendsen barostat, whereas the fifth one was done with a Monte-Carlo barostat.

For all equilibration rounds, Langevin thermostat with a collision frequency of 1 ps^{-1} was used. A positional restraint to the protein-heavy atoms with a force constant of 1.0 and 0.5 $kcal \cdot mol^{-1} \cdot Å^{-2}$ was applied to the first and second equilibration rounds, respectively. In the third round of 10 ps equilibration, a positional restraint to the backbone-heavy atoms with a force constant of 0.5 $kcal \cdot mol^{-1} \cdot Å^{-2}$ is used. The fourth and fifth equilibration of 10 ps and 1 ns, respectively, were performed without any restraint. The production runs were performed at the NVT ensemble with the Langevin thermostat with a collision frequency of 1 ps^{-1} during 100 ns for the heterocomplex TS (with the protonated and neutral catalytic K84). A total of 8 replicas of equilibration and production runs were performed reaching a total simulation time of 800 ns/system (8 replicas x 100 ns) for TS-LYN84, TS-LYS84. The MD trajectories were analyzed using the Python packages MDTraj,[30] pytraj[31] which is part of the cpptraj package,[31] MDAnalysis,[32] and PyEMMA.[33]

Shortest Path Map (SPM) calculations. The Shortest Path Map (SPM) analysis was performed using the MD simulations of the heterocomplex abba TS. However, the SPM was computed considering the monomer of a and b subunits. For SPM calculation, the MD simulations are used to compute the inter- residue mean distance and correlation matrices. A simplified graph is created using both matrices, in which only the pairs of residues that show a mean distance of less than 6 Å along the MD simulation are connected through a line. The edge connecting both residues is weighted to the Pearson correlation value (d_{ij}=-log $|C_{ij}|$). The residues with more correlated motions will be connected through a shorter line. The generated graph is further simplified to identify the shortest path lengths. Following this strategy, the residues whose lines in the graph are shorter (*i.e.*, with more correlated movements) and thus, play an important role in the conformational dynamics of an enzyme, are detected. Finally, the generated SPM graph is drawn on the 3D structure of the enzyme. More details about our SPM tool can be found in reference.[34]

Root-mean-square fluctuation (RMSF) analysis. Backbone flexibility was evaluated by RMSF analysis using MDAnalysis.[32] For each system, the protein backbone atoms, corresponding to N, Cα, C, and O atoms, were selected to perform the analysis. RMSF was first calculated separately for each trajectory, generating one independent fluctuation profile per system. For TS, the final RMSF values were obtained by residue-wise

averaging of the TS-LYN84 and TS-LYS84 RMSF profiles. Residues of interest were then extracted from the corresponding averaged RMSF profiles.

2.7 Determination of biochemical and biophysical properties of residue positions

The 85 positions that were selected for screening were distributed throughout the whole TS complex. Localization within aL2, aL6 or the COMM domain was determined from the PDB structure 1WDW, in which aL2 encompasses residues aG38–aG48, aL6 spans aS164–aI175, and the COMM domain includes residues bG97–bS185. RMSF (**Table S1**) and SPM (**Table S2**) values were obtained from MD simulations as described above. Positions with side chains exhibiting an RMSF value greater than or equal to 0.7 were categorized as 'more flexible', while those with values below 0.7 were considered 'more rigid'. Positions within 5 Å to the nearest SPM position were categorized as 'near', whereas those with at least 5 Å distance were classified as 'more distant'. Conservation values were obtained from MSAs as described above. All positions, in which the respective residue reached a conservation score exceeding 3 bits in the sequence logos (**Figure S5–S6**) were categorized as 'conserved'. Localization of residue positions within α helices, β strands, or loops was again determined from the PDB structure 1WDW. Furthermore, residues were categorized as large or small, as well as hydrophobic or hydrophilic, based on common amino acid classification.

2.8 Subcloning and site-directed mutagenesis

The genes encoding for TrpA (Uniprot-ID: Q8U094) and TrpB (Uniprot-ID: Q8U093) were codon-optimized for expression in *E. coli* and synthesized by ThermoFisher Scientific (GeneArt Strings and DNA fragments). The sequences contained BsaI restriction for further subcloning into pET21a_BsaI using the Golden Gate Cloning protocol.[35] Amber suppression codons (TAG) were introduced using site-directed mutagenesis based on a protocol from Finnzymes (ThermoFisher Scientific) followed by phosphorylation-ligation. Sequences of the resulting pET21a_TrpA and pET21a_TrpB as well as their TAG containing variants were verified by DNA sequencing (MicroSynth AG).

2.9 Midi-scale production of TrpA and TrpB variants

TrpA and TrpB variants were produced via heterologous gene expression. For each variant, *E. coli* BL21 Gold (DE3) cells (Agilent Technologies, #230132) containing pEVOL_AzoF-RS[36] were transformed with a plasmid encoding for the respective gene. A 50 mL LB_{Cm+Amp} culture, supplemented with 30 µg/mL chloramphenicol and 150 µg/mL ampicillin, was inoculated and grown at 37 °C overnight. The next day, 50 mL TB_{Cm+Amp} (+20 µM PLP for TrpB variants) were inoculated to an OD_{600} of 0.1 and incubated at 37 °C until an OD_{600} >1.2 was reached. Gene expression was induced by addition of 0.4 mM AzoF, 0.02 % L-arabinose, and 0.5 mM IPTG before the culture was incubated at 30 °C overnight. The cells were harvested by centrifugation (4000 rpm, 45 min, 20 °C) and subsequently resuspended in 5 mL 50 mM KP pH 7.5, 100 mM KCl, 10 mM imidazole. Cells were lysed by sonication (2 min, 30%, 2 s pulse, 2 pause), followed by a heat step of 15 min at 65 °C and another centrifugation step to remove *E. coli* proteins. The lysate was subjected to immobilized metal ion affinity chromatography using HisSpinTrap™ columns (GE Healthcare) following the protocol provided by the manufacturer. Elution was performed twice with 250 µL 50 mM KP pH 7.5, 100 mM KCl, 750 mM imidazole. The buffer of the eluates was exchanged to 50 mM KP pH 7.5, 100 mM KCl using NAP-5 columns (GE Healthcare) according to the manufacturer's instructions. Concentration of proteins was determined via Bradford before they were dropped into liquid nitrogen and frozen at −70 °C. The respective wild type proteins of TrpA and TrpB were expressed from pET21a_TrpA and pET21a_TrpB following the same protocol but without addition of AzoF. Purity of the proteins was assessed via SPS-PAGE.

2.10 Large scale production of TrpA and TrpB variants

Therefore, *E. coli* BL21 Gold (DE3) cells (Agilent Technologies, #230132) containing pEVOL_AzoF-RS were transformed with the respective plasmid. A 100 mL LB_{Cm+Amp} culture, supplemented with 30 µg/mL chloramphenicol and 150 µg/mL ampicillin, was inoculated with the same BL21 gold DE3 cultures containing pEVOL_AzoF-RS and the respective plasmid for each TrpA or TrpB variant. After growth at 37 °C overnight, the culture was used to inoculate 600 mL TB_{Cm+Amp} (+20 µM PLP for TrpB variants) to an OD_{600} of 0.1, which was them incubated at 37 °C until an OD_{600} >1.2 was reached. Gene expression was induced by addition of 0.4 mM AzoF, 0.02 % L-arabinose, and 0.5 mM IPTG before the culture was incubated at 30 °C overnight. The cells were harvested by

centrifugation (4000 rpm, 30 min, 20 °C) and subsequently resuspended in 50 mL 50 mM KP pH 7.5, 100 mM KCl, 10 mM imidazole. Cells were lysed by sonication (2 min, 60%, 2 s pulse, 2 pause), followed by a heat step of 15 min at 65 °C and another centrifugation step. The lysate was subjected to immobilized metal ion affinity chromatography (ÄKTA Pure, Cytiva) using a HisTrap FF Crude column (5 mL, GE Healthcare) and elution was performed with a linear gradient of imidazole (10 mM to 750 mM). Further purification and buffer exchange to 50 mM KP pH 7.5, 100 mM KCl was realized using preparative size-exclusion chromatography (ÄKTA pure, Cytiva; with Superdex 200 HiLoad 26/600 column, GE Healthcare) at 25 °C. The proteins were analyzed by SDS-PAGE and concentration was determined via Bradford before they were dropped into liquid nitrogen and frozen at −70 °C.

Wt-TrpA and wt-TrpB were expressed by transforming BL21 gold DE3 cells with either pET21a_TrpA or pET21a_TrpB, which were then used to inoculate a 250 mL LB_{Amp} culture. After cell growth at 37 °C overnight, 4x 1 L LB_{Amp} (+20 µM PLP for TrpB-wt) were inoculated to an OD_{600} of 0.1 and incubated at 37 °C until an OD_{600} of 0.6 was reached. Gene expression was induced by addition of 0.5 mM IPTG before the culture was incubated at 30 °C overnight. Harvesting of the cells and protein purification were conducted as described above.

2.11 Circular dichroism spectroscopy

Circular dichroism spectra in the far-UV range of 195–250 nm were recorded in a Jasco J-815 spectrophotometer with five accumulations and a scan speed of 50 nm/min. Spectra of 9 µM wt-TrpA, 11 µM wt-TrpB, 12 µM aF41AzoF, 11 µM aS42AzoF, 8 µM aY56AzoF, 10 µM aE174AzoF, 11 µM bL146AzoF, and 11 µM bE167AzoF were measured in 50 mM KP pH 7.5, 100 mM KCl in a 0.2 mm cuvette at 25 °C. The curves were smoothed using the Savitzky-Golay[37] method and data were normalized to obtain the mean residue ellipticity as described in ref[38].

2.12 UV/Vis spectroscopy

UV/Vis absorbance spectra of the TrpA and TrpB variants were recorded in a Jasco V650 Spectrometer in the range of 250–650 nm in a 1 cm quartz cuvette at 25 °C unless otherwise stated. Measurements were performed in 50 mM KP pH 7.5, 100 mM KCl and

concentrations are given in the respective figures. Samples were measured in TEQ, PSS^{365}, and PSS^{420}. Concentrations are given in the respective figure captions.

To assess the thermal stability of the AzoF *Z* isomer at higher temperatures, bY10AzoF (40 µM) was irradiated to establish PSS^{365} and incubated at either 50 °C or 65 °C, while spectra were recorded over time (**Figure S8**).

2.13 Estimation of isomer ratios from UV/Vis spectra

The isomer distributions of AzoF variants in TEQ, PSS^{365}, and PSS^{420} were estimated from UV/Vis spectra as reported previously.[5,6] The baseline-corrected spectra in the thermal equilibrium, as well as in the respective PSS were plotted in Origin 2022 (OriginLab). Initially, the fraction of *E* isomer present in the TEQ sample was assumed to be 100 %. Then, the spectrum of 100 % *Z* isomer was simulated based on the PSS with the highest concentration of *Z* isomer by estimating the residual fraction of *E* in this PSS using Equation 1.

$$A_Z = \frac{A_{PSS} - A_E \times f}{1 - f}$$

Equation 1

Where A_Z is the absorbance of the 100 % *Z* isomer, A_E is the absorbance of the 100% *E* isomer, A_{PSS} is the absorbance at the photostationary state, and *f* is the fraction of *E* isomer present in the PSS. The fraction *f* was manually varied to reach a point for A_Z at which the characteristic π-π* absorbance signal approximates zero under the premise that the absorbance remained positive at all wavelengths (100 % *Z* spectrum). The other PSS ratios were assigned by interpolating between the *E* and *Z* isomer spectra using Equation 2.

$$f_{PSS} = \frac{A_{PSS} - A_Z}{A_E - A_Z}$$

Equation 2

Where f_{PSS} is the fraction of *E* isomer present in the PSS and A_Z, A_E, and A_{PSS} are the absorbances at the π-π* *E* isomer absorbance maximum.

2.14 Real-time photocontrol of the TrpA reaction

TrpA activity was measured by monitoring the cleavage of IGP to yield indole and GAP. The generated GAP was oxidized to 1-arseno-3-phosphoglycerate (1-As-3-PG) in a GAPDH-dependent reaction (**Scheme S2**).[39] Thereby, the coupled reduction of NAD^+ to $NADH/H^+$ was monitored by the increase in absorbance at 340 nm [$\Delta\varepsilon_{340}(NADH/H^+–NAD^+) = 6220\ M^{-1}cm^{-1}$].The reaction is irreversible as 1-As-3-PG is rapidly hydrolyzed to arsenate and 3-phospho-glycerate (3-PG).

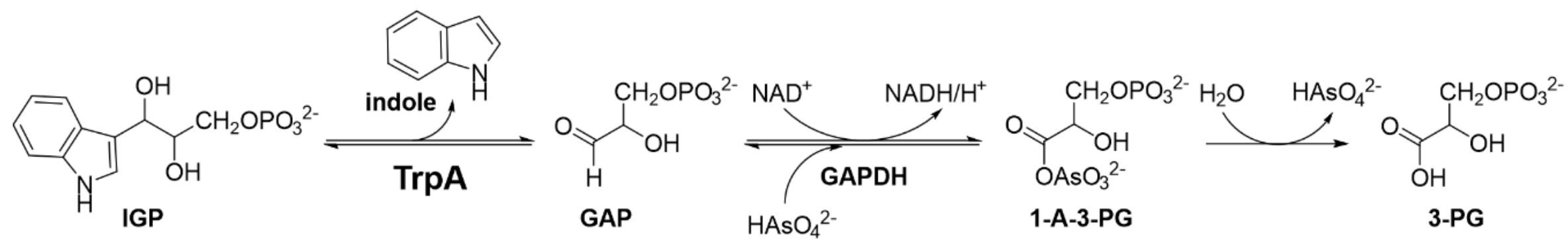

Scheme S2. Reaction assay for the determination of TrpA activity.

Measurements were performed in a JASCO V-650 UV/Vis spectrophotometer at 50 °C unless stated otherwise. Reaction conditions included 80 µM PLP, 6 mM NAD^+, 20 mM Na_3AsO_4, 5 µM GAPDH, and saturating concentrations of IGP (>250 µM) in 50 mM KP pH 7.5, 100 mM KCl at 50 °C. The reaction was started by the addition of either wt-TrpA or TrpA variants in complex with wt-TrpB. At a ratio of a : b of 1 : 1.5, wt-TrpA was fully saturated with wt-TrpB as determined from activity titration experiments (**Figure S23A**). The duration of the quasi-linear steady-state was determined from ref[8] to optimize and define the length of measurement as well as the time points of irradiation. Hence, after recording the activity in TEQ for 3 min, the sample was removed from the spectrophotometer and exposed to light of 365 nm for 2 s, followed by a 3 min measurement reflecting the activity in PSS^{365}. Subsequently, the sample was irradiated at 420 nm for 7 s before a final measurement of another 3 min, representing the activity in PSS^{420}. The activities v/E_0 (s^{-1}) in TEQ, PSS^{365} and PSS^{420} were determined from the respective slopes using the Lambert Beer Equation representative for the reaction rate v ($\mu M\ s^{-1}$), followed by normalization with the initial enzyme concentration E_0. Thereby, the initial segment of the slope was fitted for TEQ, while the second halfs of the second and third segments were fitted for PSS^{365} and PSS^{420}, respectively. In these cases, the first half of each segment showed slight perturbations in the equilibrium of the assay mixture, presumably due to damage of PLP and $NADH/H^+$, which subsided in the second half

(**Figure S24**). The LRF was calculated from the ratio of activities ($\frac{v_1}{v_2}$ with $v_1 > v_2$) between TEQ and PSS^{365} [LRF(TEQ→PSS^{365})] or PSS^{365} and PSS^{420} [LRF(PSS^{365}→PSS^{420})].

Measurements of the selected variants were performed in triplicates (1 µM TrpA variant, 1.5 µM wt-TrpB) and the mean activity v/E_0 ± S.D. as well as the mean LRF ± S.D. was determined from the activities and LRFs of the respective single measurements. For the representation of direct photocontrol experiments of the TrpA reaction, the drop in absorbance after 365 nm illumination was corrected (**Figure S24**).

2.15 Real-time photocontrol of the TrpB reaction

TrpB activity was measured in a direct spectrophotometric assay by monitoring the turnover of indole to L-tryptophan at 290 nm [$\Delta\varepsilon_{290}$(indole–Trp) = 1890 $mM^{-1}cm^{-1}$].[40] Reaction conditions included 40 mM L-serine, 250 µM indole, and 40 µM PLP in 50 mM KP pH 7.5, 100 mM KCl at 50 °C. The reaction was started by the addition of either wt-TrpB or TrpB variants in complex with wt-TrpA. At a ratio of a : b of 1 : 1.5, wt-TrpB was fully saturated with wt-TrpA as determined from activity titration experiments (**Figure S23B**). Irradiation, calculation of the activities v/E_0 (s^{-1}) in TEQ, in PSS^{365} and PSS^{420}, as well as determination of LRFs were conducted as described for the TrpA reaction. Similarly, for PSS^{365} and PSS^{420}, the second half of the segment was fitted since the initial portion was sometimes characterized by perturbations in the PLP equilibrium.

Measurements of the top candidates were performed in triplicates (1 µM TrpB-AzoF variant + 1.5 µM TrpA-wt) and the mean activity v/E_0 ± S.D. as well as the mean LRF ± S.D. was determined from the activities and LRFs of the respective single measurements.

2.16 Photocontrol of the TS reaction

TS activity was measured in a real-time photocontrol experiment in technical triplicates. The assay (10 mM L-serine, 500 µM IGP, and 40 µM PLP in 50 mM KP pH 7.5, 100 mM CsCl) was prepared in a 1.5 mL reaction vessel (1.2 mL reaction volume) and pre-incubated in a shaker at 50 °C and 300 rpm for 15 min. The reaction was started by the addition of either TrpA or TrpB variants in TEQ together with their respective wt partner (a : b was 1 : 1.5, **Figure S23**) and the samples were further incubated in a heat-block at 50 °C and 300 rpm. Aliquots representing the TEQ activity were taken at 0, 3, 6, and 9 min

and the reaction was stopped by removing the enzyme through a PES filter membrane (MWCO: 10 kDa, VWR; 13000 rpm, 2 min, 25 °C). Right after taking the 9 min sample, irradiation was conducted at 365 nm for 2 s by directly placing the LED above the reaction vessel and aliquots were taken at 12, 15, 18, and 21 min, representing the PSS^{365} activity. Subsequently, the 420 nm LED was placed above the reaction vessel and irradiation was conducted for 7 s before samples were taken at 24, 27, 30, and 33 min, representing the activity in PSS^{420}. UV/Vis measurements similar to **Figure S8** confirmed that the applied irradiation times were sufficient to completely establish the respective PSS (**Figure S19D,E**) within the employed setup.

The aliquots were subjected to HPLC using a reverse phase Zorbax Eclipse XDB-C18 column (Agilent Technologies, 80 Å, 5 µm, 4.6 x 150 mm) connected to an Agilent 1200 system. Mobile phase A consisted of 0.1% TFA in H_2O, mobile phase B of 0.1% TFA in acetonitrile. The isomers were separated at 25 °C with a flow rate of 1 mL/min with an isocratic elution at 5% B from 0–5 minutes, followed by a linear gradient from 5–95% B from 6–20 minutes. Elution of IGP and tryptophan was monitored at 280 nm and respective peaks were integrated using the integration tool embedded in the ChemStation software (Agilent Technologies, B.04.01). Decomposition of IGP and tryptophan over time at the given conditions was excluded by incubating substance-only samples at 50 °C for 60 min (**Figure S19A,B**). The concentration of converted tryptophan was determined using a standard curve in the range of 0–500 µM (**Figure S19A**).

The concentration of converted tryptophan was plotted against the time and TS activities v/E_0 ± S.E. (s^{-1}) in TEQ, PSS^{365}, and PSS^{420} were deduced from the respective slopes using a global linear fit. LRF(TEQ→PSS^{365}) and LRF(PSS^{365}→PSS^{420}) were calculated from the ratio of activities between TEQ and PSS^{365} or PSS^{365} and PSS^{420} ($\frac{v_1}{v_2}$ with $v_1 > v_2$), respectively, employing quadratic error propagation.

2.17 Classification of TrpA and TrpB variants

To classify the tested TrpA and TrpB variants with respect to their LRF, reversibility, and catalytic activity, a scoring system was applied (**Figure S16**). LRF(TEQ→PSS^{365}) and LRF(PSS^{365}→PSS^{420}) were derived from the respective activity assays (TrpA reaction for TrpA variants and TrpB reaction for TrpB variants), with the higher LRF used for subsequent classification. The LRF range, spanning from 1.0 (indicating no change in

activity upon illumination) to the highest LRF observed for a TrpA or a TrpB variant, respectively, was divided into three categories. For TrpA variants, LRF values > 2.5 were assigned a score of 3, values of 1.7–2.5 a score of 2, and values < 1.7 a score of 1. For TrpB variants, LRFs > 1.6 received a score of 3, those between 1.3 and 1.6 a score of 2, and values < 1.3 a score of 1.

For variants with an LRF greater than 1.3, reversibility of photocontrol was determined using **Equation 3**.

$$\frac{(1 - \mathrm{LRF}^{\mathrm{a.i.}/365})}{(1 - \mathrm{LRF}^{365/420})}$$

Equation 3

These variants were grouped into three categories based on the percentage of reversible recovery. Variants showing > 66% reversibility received a score of 3, those with 33–66% a score of 2, and those with < 33% a score of 1.

In addition, TrpA or TrpB activity was also classified. Thereby, the highest activity of a variant, regardless of whether it occurred in TEQ, PSS^{365}, or PSS^{420}, was taken into account. For TrpA variants, activities > 2.7 min^{-1} received a score of 3, those between 1.3 and 2.7 min^{-1} a score of 2, and those < 1.3 min^{-1} a score of 1. For TrpB variants, scores of 3, 2, and 1 were assigned to activities > 20 min^{-1}, between 10 and 20 min^{-1}, and < 10 min^{-1}, respectively.

Finally, the scores obtained for LRF, reversibility and activity were multiplied (**Table S4–5**). TrpA variants with a final score of ≥ 9 (aF41AzoF, aS42AzoF, aY56AzoF, and aE174AzoF) and TrpB variants with a final score of ≥ 4 (bA149AzoF and bE167AzoF) were selected for further evaluation.

References

[1] M. Dong, A. Babalhavaeji, S. Samanta, A. A. Beharry and G. A. Woolley, "Red-Shifting Azobenzene Photoswitches for in Vivo Use," *Accounts of chemical research* 48, no. 10 (2015): 2662. https://doi.org/10.1021/acs.accounts.5b00270.

[2] D. Bléger and S. Hecht, "Visible-Light-Activated Molecular Switches," *Angewandte Chemie (International ed. in English)* 54, no. 39 (2015): 11338. https://doi.org/10.1002/anie.201500628.

[3] M. Gao, D. Kwaria, Y. Norikane and Y. Yue, "Visible-light-switchable azobenzenes: Molecular design, supramolecular systems, and applications," *Natural Sciences* 3, no. 1 (2023): e220020. https://doi.org/10.1002/ntls.20220020.

[4] S. A. Ahmed, P. McPhie and E. W. Miles, "A thermally induced reversible conformational transition of the tryptophan synthase beta2 subunit probed by the spectroscopic properties of pyridoxal phosphate and by enzymatic activity," *Journal of Biological Chemistry* 271, no. 15 (1996): 8612. https://doi.org/10.1074/jbc.271.15.8612.

[5] J. Calbo, C. E. Weston, A. J. P. White, H. S. Rzepa, J. Contreras-García and M. J. Fuchter, "Tuning Azoheteroarene Photoswitch Performance through Heteroaryl Design," *Journal of the American Chemical Society* 139, no. 3 (2017): 1261. https://doi.org/10.1021/jacs.6b11626.

[6] C. Hiefinger, M. Marcon and V. Pape et al., "Expanding the Repertoire of Photoswitchable Unnatural Amino Acids for Enzyme Engineering," *Angewandte Chemie (International ed. in English),* no. 64 (2025): 38:e202508562. https://doi.org/10.1101/2025.04.17.649278.

[7] M. Wieland, J. Luizaga and C. Duran et al., "Reversible Substrate-Specific Photocontrol of the Chemotherapeutic Asparaginase(-Glutaminase) from Escherichia coli," *ACS catalysis* 15, no. 10 (2025): 8462. https://doi.org/10.1021/acscatal.5c01608.

[8] C. Hiefinger, S. Mandl, M. Wieland and A. Kneuttinger, "Rational design, production and in vitro analysis of photoxenoproteins," *Methods in enzymology* 682 (2023): 247. https://doi.org/10.1016/bs.mie.2022.12.003.

[9] A. Merz, T. Knöchel, J. N. Jansonius and K. Kirschner, "The hyperthermostable indoleglycerol phosphate synthase from Thermotoga maritima is destabilized by mutational disruption of two solvent-exposed salt bridges," *Journal of molecular biology* 288, no. 4 (1999): 753. https://doi.org/10.1006/jmbi.1999.2709.

[10] A. C. Kneuttinger, S. Zwisele and K. Straub et al., "Light-Regulation of Tryptophan Synthase by Combining Protein Design and Enzymology," *International journal of molecular sciences* 20, no. 20 (2019). https://doi.org/10.3390/ijms20205106.

[11] N. Mulder and R. Apweiler, "InterPro and InterProScan: tools for protein sequence classification and comparison," *Methods in molecular biology (Clifton, N.J.)* 396 (2007): 59. https://doi.org/10.1007/978-1-59745-515-2_5.

[12] A. M. Waterhouse, J. B. Procter, D. M. A. Martin, M. Clamp and G. J. Barton, "Jalview Version 2--a multiple sequence alignment editor and analysis workbench," *Bioinformatics (Oxford, England)* 25, no. 9 (2009): 1189. https://doi.org/10.1093/bioinformatics/btp033.

[13] G. E. Crooks, G. Hon, J.-M. Chandonia and S. E. Brenner, "WebLogo: a sequence logo generator," *Genome research* 14, no. 6 (2004): 1188. https://doi.org/10.1101/gr.849004.

[14] J. Jumper, R. Evans and A. Pritzel et al., "Highly accurate protein structure prediction with AlphaFold," *Nature* 596, no. 7873 (2021): 583. https://doi.org/10.1038/s41586-021-03819-2.

[15] M. Ester, H. P. Kriegel, J. Sander and X. Xu, "A Density-Based Algorithm for Discovering Clusters in Large Spatial Databases with Noise," *KDD'96: Proceedings of the Second International Conference on Knowledge Discovery and Data Mining (*1996): 226.

[16] M. Jukič, J. Konc, S. Gobec and D. Janežič, "Identification of Conserved Water Sites in Protein Structures for Drug Design," *Journal of chemical information and modeling* 57, no. 12 (2017): 3094. https://doi.org/10.1021/acs.jcim.7b00443.

[17] F. Pedregosa, G. Varoquaux and A. Gramfort et al., "Scikit-learn: Machine learning in Python," *Journal of Machine Learning Research,* no. 12 (2011): 2825.

[18] Da Case, K. Belfon and I. Y. Ben-Shalom et al., *AMBER20*, 2020.

[19] J. Wang, R. M. Wolf, J. W. Caldwell, P. A. Kollman and D. A. Case, "Development and testing of a general amber force field," *Journal of computational chemistry* 25, no. 9 (2004): 1157. https://doi.org/10.1002/jcc.20035.

[20] C. N. Schutz and A. Warshel, "What are the dielectric "constants" of proteins and how to validate electrostatic models?," *Proteins* 44, no. 4 (2001): 400. https://doi.org/10.1002/prot.1106.

[21] C. I. Bayly, P. Cieplak, W. Cornell and P. A. Kollman, "A well-behaved electrostatic potential based method using charge restraints for deriving atomic charges: the RESP model," *The Journal of Physical Chemistry* 97, no. 40 (1993): 10269. https://doi.org/10.1021/j100142a004.
[22] U. C. Singh and P. A. Kollman, "An approach to computing electrostatic charges for molecules," *Journal of computational chemistry* 5, no. 2 (1984): 129. https://doi.org/10.1002/jcc.540050204.
[23] Frisch, M.J., Trucks, G.W., Schlegel, H.B., Scuseria, G.E., Robb, M.A., Cheeseman, J.R., et al., "Gaussian 16 Rev. C.01.," *Wallingford, CT. (*2016).
[24] M. H. M. Olsson, C. R. Søndergaard, M. Rostkowski and J. H. Jensen, "PROPKA3: Consistent Treatment of Internal and Surface Residues in Empirical pKa Predictions," *Journal of chemical theory and computation* 7, no. 2 (2011): 525. https://doi.org/10.1021/ct100578z.
[25] C. R. Søndergaard, M. H. M. Olsson, M. Rostkowski and J. H. Jensen, "Improved Treatment of Ligands and Coupling Effects in Empirical Calculation and Rationalization of pKa Values," *Journal of chemical theory and computation* 7, no. 7 (2011): 2284. https://doi.org/10.1021/ct200133y.
[26] M. F. Dunn, "Allosteric regulation of substrate channeling and catalysis in the tryptophan synthase bienzyme complex," *Archives of biochemistry and biophysics* 519, no. 2 (2012): 154. https://doi.org/10.1016/j.abb.2012.01.016.
[27] C. Tian, K. Kasavajhala and K. A. A. Belfon et al., "ff19SB: Amino-Acid-Specific Protein Backbone Parameters Trained against Quantum Mechanics Energy Surfaces in Solution," *Journal of chemical theory and computation* 16, no. 1 (2020): 528. https://doi.org/10.1021/acs.jctc.9b00591.
[28] D. R. Roe and T. E. Cheatham, "PTRAJ and CPPTRAJ: Software for Processing and Analysis of Molecular Dynamics Trajectory Data," *Journal of chemical theory and computation* 9, no. 7 (2013): 3084. https://doi.org/10.1021/ct400341p.
[29] T. Darden, D. York and L. Pedersen, "Particle mesh Ewald: An N ·log(N) method for Ewald sums in large systems," *The Journal of Chemical Physics* 98, no. 12 (1993): 10089. https://doi.org/10.1063/1.464397.
[30] R. T. McGibbon, K. A. Beauchamp and M. P. Harrigan et al., "MDTraj: A Modern Open Library for the Analysis of Molecular Dynamics Trajectories," *Biophysical journal* 109, no. 8 (2015): 1528. https://doi.org/10.1016/j.bpj.2015.08.015.
[31] N. Hai, R. D. R., S. Jason and C. D. A., *PYTRAJ v1.0.0.dev1: Interactive data analysis for molecular dynamics simulations*, Zenodo, 2016.
[32] R. Gowers, M. Linke and J. Barnoud et al., "MDAnalysis: A Python Package for the Rapid Analysis of Molecular Dynamics Simulations," in *Proceedings of the 15th Python in Science Conference (*2016): 98.
[33] M. K. Scherer, B. Trendelkamp-Schroer and F. Paul et al., "PyEMMA 2: A Software Package for Estimation, Validation, and Analysis of Markov Models," *Journal of chemical theory and computation* 11, no. 11 (2015): 5525. https://doi.org/10.1021/acs.jctc.5b00743.
[34] S. Osuna, "The challenge of predicting distal active site mutations in computational enzyme design," *WIREs Computational Molecular Science* 11, no. 3 (2021): e1502. https://doi.org/10.1002/wcms.1502.
[35] B. Rohweder, F. Semmelmann, C. Endres and R. Sterner, "Standardized cloning vectors for protein production and generation of large gene libraries in Escherichia coli," *BioTechniques* 64, no. 1 (2018): 24. https://doi.org/10.2144/000114628.
[36] M. Bose, D. Groff, J. Xie, E. Brustad and P. G. Schultz, "The incorporation of a photoisomerizable amino acid into proteins in E. coli," *Journal of the American Chemical Society* 128, no. 2 (2006): 388. https://doi.org/10.1021/ja055467u.
[37] A. Savitzky and M. J. E. Golay, "Smoothing and Differentiation of Data by Simplified Least Squares Procedures," *Analytical chemistry* 36, no. 8 (1964): 1627. https://doi.org/10.1021/ac60214a047.
[38] S. M. Kelly, T. J. Jess and N. C. Price, "How to study proteins by circular dichroism," *Biochimica et biophysica acta* 1751, no. 2 (2005): 119. https://doi.org/10.1016/j.bbapap.2005.06.005.
[39] T. E. Creighton, "A steady-state kinetic investigation of the reaction mechanism of the tryptophan synthetase of Escherichia coli," *European journal of biochemistry* 13, no. 1 (1970): 1. https://doi.org/10.1111/j.1432-1033.1970.tb00892.x.
[40] E. J. Faeder and G. G. Hammes, "Kinetic studies of tryptophan synthetase. Interaction of substrates with the B subunit," *Biochemistry* 9, no. 21 (1970): 4043. https://doi.org/10.1021/bi00823a003.
